# AFLP Approach Reveals Variability in *Phragmites australis*: Implications for Its Die-Back and Evidence for Genotoxic Effects

**DOI:** 10.3389/fpls.2018.00386

**Published:** 2018-03-26

**Authors:** Andrea Coppi, Lorenzo Lastrucci, David Cappelletti, Martina Cerri, Francesco Ferranti, Valentina Ferri, Bruno Foggi, Daniela Gigante, Roberto Venanzoni, Daniele Viciani, Roberta Selvaggi, Lara Reale

**Affiliations:** ^1^Department of Biology, University of Florence, Florence, Italy; ^2^Department of Chemistry, Biology and Biotechnology, University of Perugia, Perugia, Italy; ^3^Department of Agricultural, Food and Environmental Sciences, University of Perugia, Perugia, Italy

**Keywords:** outlier loci, heavy metals, DNA fingerprinting, common reed, wetlands, Central Italy

## Abstract

*Phragmites australis* is a subcosmopolitan species typical of wetlands being studied in Europe for its disappearance from natural stands, a phenomenon called reed die-back syndrome (RDBS). Although it is conjectured that low genetic variability contributes to RDBS, this aspect remains neglected to this day. Using a molecular fingerprinting approach and a sequence analysis of the trnT-trnL/rbcL-psaI regions of cpDNA, this study aimed to compare the genetic structure of stable vs. RDBS-affected *P. australis* stands from five wetlands of central Italy. Beforehand, in order to characterize the health condition of reed populations, the occurrence of the main macromorphological descriptors for RDBS was considered on 40 reed stands. Soil samples were also collected to examine the total content of heavy metals. The current study analyzed cpDNA in 19 samples and AFLP profiles in 381 samples to investigate the genetic structure of *Phragmites* populations. Based on the multinomial-Dirichlet model, an analysis of candidate loci under selective pressure was also performed. The relationships among AFLP data, RDBS descriptors and chemicals were evaluated with the use of Linear Mixed Models. The analysis of the cpDNA shows the occurrence of the haplotypes M (the most widespread), and K here recorded for the first time in Italy. Three new haplotypes were also described. The DNA fingerprinting analysis has produced a total of 322 loci (98% polymorphic) and shows the medium-to-high amount of genetic diversity. The significant genetic differentiation among wetlands (*F*_st_ = 0.337) suggests either low gene flow or small effective population size. Moreover, the low amount of outlier loci (only 5; l.5% of the total), seems to indicate the scarce occurrence of selective pressure upon the reed’s genome. Genetic diversity increased in relationship to the decrease in diameter and of flowering buds of the reed, two of the trends associated with the die-back. The current study rejects the hypothesis that genetic diversity massively contributed to RDBS. Moreover, significant relationships between genetic diversity and the total concentration of some heavy metals (Cr, Cu, and Zn) were highlighted, indicating possible genotoxic effects on *P. australis*. The current study represents a fact-finding background useful for the conservation of common reed.

## Introduction

Common reed ‘die-back’ syndrome (hereafter RDBS) refers to the non-reversible, spontaneous retreat or disappearance of mature stands of the common reed, *Phragmites australis* (Cav.) Steud. (van der Putten, 1997). RDBS appeared, at first, to be limited to areas of central, eastern, and northern Europe (van der Putten, 1994), but its more recent discovery in several Italian wetlands (Fogli et al., 2002; Gigante et al., 2011, 2013, 2014; Lastrucci et al., 2016) has highlighted this ongoing common reed retreat in the Mediterranean basin as well. The syndrome manifests itself with the occurrence of a clear morphological pattern, including a set of traits showing a stunted growth of reed, e.g., short and thin culm, the presence of dead buds, and a high incidence of clumping (Armstrong et al., 1996; van der Putten, 1997; Clevering, 1998; Gigante et al., 2011, 2014). The latter characteristic was shown to be a reliable diagnostic trait for RDBS detection (Lastrucci et al., 2017). Although this phenomenon is generally known and described in the literature, the main processes underpinning RDBS remain largely unresolved. Among the first factors hypothesized as possible causes of reed retreat in Europe, the main ones were land reclamation, impact of recreational activities, mechanical damage, livestock grazing, bad water/sediment quality, and water regulation (Ostendorp, 1989). Specific relationships between RDBS and several ecological features, such as exposure to permanent flooding or chemicals in the local sediments or waters, were found for Italian wetlands (Gigante et al., 2011, 2014; Lastrucci et al., 2016).

Among the putative drivers of RDBS, a possible role of genetic diversity was also hypothesized (van der Putten, 1997), with some pilot studies finding low genetic variability within those *P. australis* populations affected by die-back (Neuhaus et al., 1993). This consideration and work originated in the evidence for clonal expansion predominating over plant establishment from seed for reed beds during an advanced state of colonization, which led to the hypothesis of a reduced genetic variation within such populations (van der Putten, 1997). These common reed stands may, therefore, be unable to adapt to rapid changes in their site conditions. It seems reasonable to ask, then, whether low levels of genetic diversity occur in common reed stands showing signs of decline or undergoing RDBS. However, van der Putten (1997) concluded that the relationship between genetic variation and RDBS is likely more complex than expected. Indeed, the first local molecular survey revealed that the affected reed populations might be highly variable with respect to their clonal structure (van der Putten, 1997). Given these first considerations, studying the genetic diversity that characterizes the vigorous and suffering common reed stands underscores the first hypothesis of the present study, based on which, low genetic diversity potentially characterize RDBS affected *P. australis* stands. Further, by adopting the DNA fingerprinting approach, two additional hypotheses were formulated.

According to the second hypothesis, it was assumed that variation in the environmental factors that potentially induce RDBS might exert a selective pressure upon the common reed’s genome. Both permanent submersion and water depth were recently linked to the occurrence of RDBS-affected stands (Lastrucci et al., 2017). In particular, relationships between the high incidence of clumping and deep permanent submersion of the stand were first suggested (Armstrong et al., 1996; van der Putten, 1997; Gigante et al., 2011, 2014) and then clearly demonstrated by Lastrucci et al. (2017). Based on the idea that the morphological pattern characterizing the declining stands might give rise to a genetic drift in the RDBS-affected populations it was hypothesized that finding evidence of outlier loci (those beyond the expected distribution of genetic pattern with an unusual frame of higher differentiation) could be used to help in explaining the levels of genetic variation among the populations (Beaumont and Nichols, 1996; Beaumont and Balding, 2004). Building on this, relationships between such outlier loci and the diagnostic traits of RDBS could serve as a proxy for the health status of these populations.

Finally, in the third hypothesis it was suggested that a high concentration of chemicals in the rhizosphere can induce random variations in the genomes of plants (Labra et al., 2003). If so, this could plausibly increase diversity at intra-population level while reducing population differentiation (Mengoni et al., 2006). In particular, it is well known that heavy metal accumulation in aquatic macrophytes may be affected by biotic and/or abiotic factors, such as anatomical features, the age of the individuals (e.g., generation time), temperature and pH, as well as the total concentration of elements in the water and sediment (Lin and Zhang, 1990). Several studies noticed that *P. australis* reflect the cumulative effect of environmental pollution, and the concentration of some elements (e.g., Cd, Cr, and Ni) could exceed the toxicity threshold in the aerial portion of this species (Bragato et al., 2006; Bonanno and Lo Giudice, 2010). Moreover, recent studies have reported evidence on the role of As, Cd, Cr, Cu, and Ni in DNA damage (Gjorgieva et al., 2013; Kim et al., 2015).

The current study had the objectives of testing the three hypotheses presented above: (i) To compare the genetic structure of vigorous stands of *P. australis* with those already verified as having RDBS; however, before analyzing their genetic structure, a preliminary survey of the cpDNA diversity was required to characterize and examine the haplotypes in the *P. australis* populations; (ii) To evaluate the frequencies of outlier loci and their relation to demographic and morphological traits indicators of RDBS; and (iii) To evaluate the possible genotoxic effect on the populations due to heavy metals detected in the rhizosphere.

## Materials and Methods

### Study Area, Sampling Design, and Macro-Morphological Plant Traits

**FIGURE 1:**
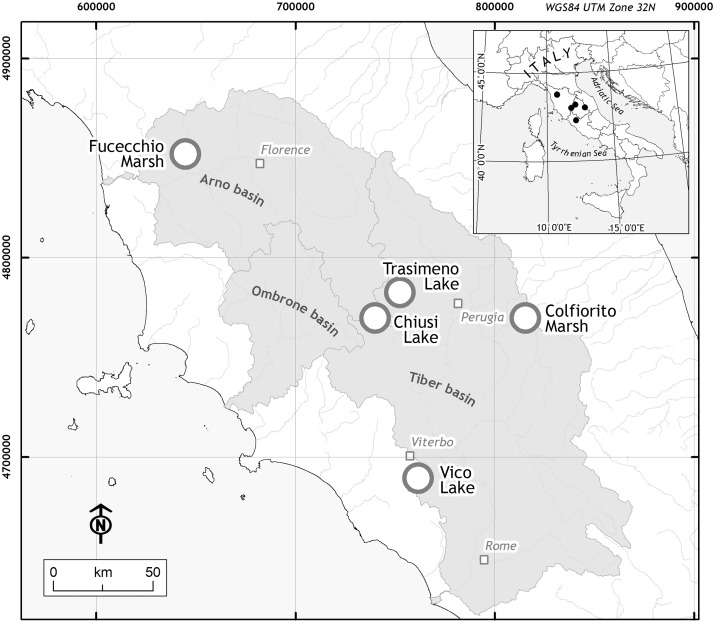
Locations of the study area. The five sampling sites (white dot) were located in three Italian Regions: Tuscany for Fucecchio Marsh (‘*Fu*’) and Chiusi Lake (‘*Ch*’); Umbria for Colfiorito Marsh (‘*Co*’) and Trasimeno Lake (‘*Tr*’); Latium for Vico Lake (‘*Vi*’).

The study area included five wetlands of central Italy (**Figure 1**): Fucecchio Marshland, at Le Morette (north-western Tuscany, henceforth ‘*Fu*’; 43° 48′ 30.38′′ N, 10° 48′ 20.14′′ E); Chiusi lake (south-eastern Tuscany, ‘*Ch*’; 43° 03′ 22.11′′ N, 11° 57′ 55.79′′ E); Trasimeno lake (north-western Umbria, ‘*Tr*’; 43° 08′ 05.5′′ N 12°06′ 04.6′′ E); Colfiorito marsh (eastern Umbria, ‘*Co*’; 43° 01′ 23′′ N, 12° 52′36′′ E) and Vico lake (north-western Lazio, ‘*Vi*’; 42° 18′ 58.40′′ N, 12° 10′ 5.89′′ E). The five sites differ in their pedo-morphological characteristics, passing from lowland marshlands (‘*Fu*’) or lakes (‘*Ch*’ and ‘*Tr*’) to the Apennine mountain marshland (‘*Co*’) or a volcanic basin (‘*Vi*’), and surface areas, with the smallest site (‘*Fu*’) covering 1.02 km^2^ to the largest wetland (‘*Tr*’) (Trasimeno lake is among the largest in Italy with an area of 121.5 km^2^).

The five areas had been formerly investigated in order to understand the RDBS process and diagnostic traits, by way of, stratified random sampling including two ecologically distinct conditions: (1) ‘flooded stands,’ submerged by water also in the driest season (August–September) and (2) ‘emerged stands,’ more or less long-flooded during the year, but not so during the driest season (for more detailed information about the methodology, see Lastrucci et al., 2017). These two distinct ecological conditions were based on the later demonstrated assumption that permanent submersion is the main driver of reed decline (Gigante et al., 2011, 2014; Lastrucci et al., 2016, 2017). The same plots sampled by Lastrucci et al. (2017) were used for the sampling design of the present study, thus benefiting from their attribution to a status of being or not-being affected by the RDBS, as resulting in the mentioned study (for more details see Lastrucci et al., 2017). At each site, eight 1-m^2^ plots (four flooded and four emerged) were randomly selected, for a total of 40 plots sampled. Macromorphological traits accounting for RDBS (i.e., culm diameter and height, dead apical bud percentage, culm flowering percentage, clumping percentage, and culm density) were available according to the methods of Gigante et al. (2011, 2014) and Lastrucci et al. (2016, 2017). Additionally, for each plot, sediment samples were collected with a core driller at a depth of ca. 30 cm; each sediment sample was transferred to a glass bottle and stored in a fridge box.

For the molecular analysis, each selected plot was considered as the center of a circle with a radius of 10 m in which foliar tissue samples were randomly collected from at least 10 individuals spaced about 5 m apart. Sampling was carried out at the end of the vegetative season, corresponding to the reaching of the maximum standing crop of *P. australis* (i.e., end of August 2014).

### DNA Isolation and Molecular Approaches

Each leaf tissue sample was first dried on silica-gel and ground in a mortar with sterile sand. The DNA was extracted by using the 2x cetyltrimethylammonium bromide (CTAB) protocol (Doyle and Doyle, 1990). The quality and quantity of the extracted DNA were checked by a spectrometric survey that used a Bio-Photometer (Eppendorf).

### Haplotype Analysis

Amplification of the trnT-trnL and rbcL-psaI intergenic spacer regions of the cpDNA was performed, following the protocol described by Taberlet et al. (1991) and Saltonstall (2001, 2002), for 2–5 individuals per lake site. Automated DNA sequencing was performed directly from the purified PCR products, by using the BigDye Terminator v2 chemistry, and a sequencer (ABI310; PE-Applied Biosystems, Norwalk, CT, United States). To reduce any artifacts, all the samples were amplified and sequenced twice, with both forward and reverse primers. The original sequences of *P. australis* were edited in BioEdit v7.0 (Hall, 1999) and they were checked for orthology via comparisons with 34 accessions retrieved from the United States National Center for Biotechnology Information (NCBI) database. A single dataset that consisted of the concatenated sequences of the intergenic spacer regions (trnT-trnL+rbcL-psaI) underwent fitting and analysis. The reconstruction of the *P. australis* haplotype sequences followed the code reported by Saltonstall (2001, 2002), and implemented more recently by Hauber et al. (2011) and Lambertini et al. (2012). Multiple alignments were performed with MAFFT v5, with G-INS-I used as the iterative refinement method for the multi-alignment (Katoh and Standley, 2013). The networks of the haplotypes were constructed in the R environment, based on the “pegas” package (Paradis, 2010). Estimates of the genetic distances were implemented within each group of haplotypes, and between all groups of haplotypes (**Table 1** and Supplementary Table S1). The haplotype AA (HapAA) from North America was then removed from the analysis because of missing data in the rbcL-psaI region (position 1385–1659 of the alignment; see the alignment in Supplementary Table S2). The analyses were conducted by using the Kimura two-parameter model (Kimura, 1980) and the MEGA6.06 software (Tamura et al., 2013). The analyses involved 19 samples of *P. australis* from Central Italy (see **Table 1**), 32 *Phragmites* s.l. sequences (see Supplementary Table S1), plus two samples that were selected as outgroups: *Hakonechloa macra* (NC 025235) and *Monachather paradoxus* (KF169836-KF169834). A total of 53 sequences were grouped on the basis of a previous parsimony network analysis (Saltonstall, 2002), taking into consideration the taxonomy and geographic provenance of the haplotypes, as recommended by Saltonstall (2002) and Lambertini et al. (2012). The number of base substitutions per site was calculated by averaging over all the sequence pairs within each group, and the standard error estimate(s) were obtained by using a bootstrap procedure (*n* = 1000 replicates; Efron, 1979).

**Table 1:** List of sampling sites examined for both AFLP and trnL-trnF/rbcl-psaI analyses with: accession codes; main geographic distributions; grouping affiliation codes for analysis of genetic distance (Group); GenBank accessions and haplotype classification.

ID	Distribution	Group	trnT-trnL	rbcl-psaI	Hap.
Ch01	Chiusi lake, Italy	*P. australis* s.l. group	KY511383	KY511402	M
Ch02	Chiusi lake, Italy	*P. australis* s.l. group	KY511387	KY511404	K
Ch03	Chiusi lake, Italy				
Ch04	Chiusi lake, Italy	*P. australis* s.l. group	KY511378	KY511406	M
Ch05	Chiusi lake, Italy				
Ch06	Chiusi lake, Italy	*P. australis* s.l. group	KY511373	KY511405	CHTR
Ch11	Chiusi lake, Italy				
Ch12	Chiusi lake, Italy				
Co01	Colfiorito marsh, Italy	*P. australis* s.l. group	KY511384	KY511397	CO
Co02	Colfiorito marsh, Italy				
Co03	Colfiorito marsh, Italy				
Co04	Colfiorito marsh, Italy	*P. australis* s.l. group	KY511384	KY511397	CO
Co09	Colfiorito marsh, Italy				
Co10	Colfiorito marsh, Italy				
Co11	Colfiorito marsh, Italy	*P. australis* s.l. group	KY511385	KY511391	CO
Co12	Colfiorito marsh, Italy				
Fu01	Fucecchio marsh, Italy	*P. australis* s.l. group	KY511382	KY511393	M
Fu02	Fucecchio marsh, Italy	*P. australis* s.l. group	KY511375	KY511395	M
Fu04	Fucecchio marsh, Italy				
Fu06	Fucecchio marsh, Italy				
Fu09	Fucecchio marsh, Italy				
Fu10	Fucecchio marsh, Italy				
Fu11	Fucecchio marsh, Italy	*P. australis* s.l. group	KY511376	KY511396	M
Fu12	Fucecchio marsh, Italy	*P. australis* s.l. group	KY511376	KY511396	M
Tr01	Trasimeno lake, Italy	*P. australis* s.l. group	KY511380	KY511398	M
Tr02	Trasimeno lake, Italy				
Tr03	Trasimeno lake, Italy	*P. australis* s.l. group	KY511377	KY511399	M
Tr04	Trasimeno lake, Italy				
Tr09	Trasimeno lake, Italy				
Tr10	Trasimeno lake, Italy	*P. australis* s.l. group	KY511377	KY511399	M
Tr11	Trasimeno lake, Italy	*P. australis* s.l. group	KY511372	KY511401	CHTR
Tr12	Trasimeno lake, Italy				
Vi01	Vico lake, Italy	*P. australis* s.l. group	KY511390	KY511408	VI
Vi03	Vico lake, Italy	*P. australis* s.l. group	KY511388	KY511407	VI
Vi04	Vico lake, Italy				
Vi06	Vico lake, Italy				
Vi09	Vico lake, Italy				
Vi10	Vico lake, Italy				
Vi11	Vico lake, Italy				
Vi16	Vico lake, Italy	*P. australis* s.l. group	KY511389	KY511409	VI

### Amplified Fragment Length Polymorphism (AFLP) Protocol

The AFLP analysis followed standard procedure, but with minor modifications of previous studies using molecular tools (see Coppi et al., 2014 and references therein). Twelve primer-pair combinations (Supplementary Table S3) were tested on three individuals from each examined wetland to screen the combination that produces the most informative, readable, and repeatable profiles. Two combinations of primers were selected for the final analysis: hex_EcoRI-CTA/MseI-ATG and fam_EcoRI-TAC/MseI-ATG.

Analysis of the AFLP profiles obtained by capillary electrophoresis was performed with GeneMarker v1.5 (SoftGenetics LLC, State College, PA, United States). A cut-off value, fixed at 5% of the maximum profile showed in the chromatograms, was decided upon after the analysis of replicate samples, taking into account only those bands present in all the replicates.

### Outlier Detection

To detect outliers it was used BayeScan v2.01. This software is used primarily for highly polymorphic dominant markers, such as AFLP; it allows the estimation of the posterior probability of a given locus under selection (Foll and Gaggiotti, 2008; Yang et al., 2016). The Bayesian method, here adopted for the detection of outlier loci, assumes that the locus frequencies within a population follow a multivariate β-distribution (Rannala, 1996; Rannala and Hartigan, 1996; Burr, 2000) as a function of the multilocus Fixation Index value and the average of locus frequencies of each locus between populations. With the estimation of the *F*_st_ coefficients, as done here by the Bayesian method, the AFLP loci under selection could be quantified (Foll and Gaggiotti, 2008). A limit value for detecting the loci under selection was evaluated following the BayeScan manual: values of log (PO) > 0.5 to 1 were substantial; log (PO) > 1 to 1.5 were strong; log (PO) > 1.5 to 2 were very strong, and log (PO) > 2.0 to infinite was considered as decisive in the loci selection. The BayeScan analysis was carried out following Yang et al. (2016). The number of pilot runs was kept at 20, with a length of 10 000 iterations each one.

### Genetic Variation at Intra- and Inter-Population Level

Within-population average genetic diversity, hereafter AGD, was expressed as the computed probability that two randomly chosen homologous sites are different (Nei, 1987) by using the program Arlequin v2.000 (Schneider et al., 2000). The AGD values were calculated firstly for the total of the AFLP loci here detected (AGD_all), and then separately for the outlier and neutral loci (AGD_out and AGD_neut, respectively). The latter were obtained after the exclusion of outlier loci from the total ones analyzed. The differences in AGD among the populations were studied using analysis of variance (ANOVA); the normality of the residuals was tested with the Shapiro–Wilk normality test. After the ANOVA, a *post hoc* Tukey test determined on a pairwise basis which among the five sites were statistically different from each other. The Shapiro–Wilk and Tukey tests, and the ANOVA, were performed using the R software platform v3.2.3 (R Core Team, 2017).

Analysis of molecular variance (AMOVA, Excoffier et al., 1992), implemented in Arlequin v2.000 (Schneider et al., 2000), was used to analyze the partitioning of total genetic variation at three different hierarchical levels: within populations, among populations, and among hypothetical groups of populations. Statistical support for the different hypothetical groupings of populations, based on geographical distribution and ecology (emerged vs. flooded), was tested in terms of the variance components and the percentage of explained variation. Genetic distances between populations were estimated by computing a matrix of Slatkin’s linearized pairwise *F*_st_ values (Slatkin, 1995); this was then used to generate a neighbor-joining (Saitou and Nei, 1987) dendrogram with the software Mega 6 (Tamura et al., 2013). The number of migrants per generations (*N*_m_) was then calculated directly from the measure of population differentiation (*F*_st_) as: *N*_m_ = (1-*F*_st_)/4*F*_st_ (Slatkin, 1987). As suggested by Whitlock and McCauley (1999), the assumptions underlying the *N*_m_ model are likely to be violated by most of the natural populations. Nevertheless, the *N*_m_ model is still widely used. Indeed, following Nielsen (2005) and Holsinger and Weir (2009), directional selection acting differentially among populations, tend to increase the *F*_st_ values compared to the prediction of Wright’s formula, whereas balancing selection acts to decrease *F*_st_. Based on these assumptions, the Nm formula still makes sense and can be used as a proxy for the action of selection.

### Heavy Metals Pollutants Analysis

The concentrations of heavy metal pollutants were determined for Cd, Pb, Zn, Cr, Ni, Cu. These heavy metals were considered due to their high environmental significance (Farkas et al., 2007 and references therein), and because analyzed also in the previous studies about the reed bed health status in Central Italy (Gigante et al., 2014; Lastrucci et al., 2016). The total concentration of heavy metals were determined for all the sediment samples by Inductively Coupled Plasma Optical Emission Spectroscopy (ICP-OES, Ultima 2 HORIBA Scientific). The spectrometer was equipped with an ultrasonic nebulizer (U-5000AT, CETAC Technologies) and operated with a flow rate of 1 L min^-1^ and a pressure of 2.5 bars, to improve the instrument’s detection limits which ranged from 0.1 to 1.5 mg⋅kg^-1^. Commercially produced (ICP multi-element standard solution IV CertiPUR^®^, VWR Merck Chemicals and Reagents) standard solutions (1000 mg⋅L^-1^) in nitric acid were used to prepare the appropriate elemental calibration standards.

Prior to analysis, an acid digestion was performed on the sediment samples (MATT and APAT, 2005; Goretti et al., 2016). The latter were air-dried, disaggregated by using a mortar and pestle to pass through a 2-mm mesh sieve, then dried at 105°C for 24 h. A total of 8 mL of ultrapure nitric acid (Millipore Suprapur^®^, 65%) and 2 ml of ultrapure solution of hydrogen peroxide (Millipore Suprapur^®^, 30%) were added to 0.200 g from each dried sediment sample and digested in a Mars microwave oven (working at a power of 1000 W). Microwave digestion consisted of two steps: 130°C (200 psi) for 1 min, and 180°C (300 psi) for 10 min. The mixture was cooled, filtered (Whatman Grade No. 42, particle retention 2.5 mm), and diluted with ultrapure water (18.2 MΩ) to 50 ml. The heavy metal concentrations were measured based on two replicates of the overall analytical procedure for each sample of sediment examined. Differences among the chemical sediment composition of the five studied sites have been tested adopting an ANOVA. A Shapiro–Wilk test was performed in order to test the normality of Residuals (Shapiro and Wilk, 1965). After the ANOVA a *Post Hoc* Tukey test was carried out to put in evidence the differences between the five wetlands (Tukey, 1962). In order to obtain the normality of residuals, when necessary, a log-transformation of data was applied.

### Correlations Analyses Between Genomic Data and the Macro-Morphological/ Chemicals Data

For each AGD values (AGD_all, AGD_neut and AGD_out), relationships with heavy metals and the ecological status were analyzed through a linear mixed-effects model considering the genetic diversity as response variable, the metals and the status (flooded/emerged) as main factors (also considering the interaction terms among each metal and the status) and the sites as random factor. The use of mixed models and random effects allowed to take in account the spatial correlation among the observation from the same site and obtain more general and robust results (Bolker et al., 2009). The normality of the residuals was tested by a Shapiro–Wilk test and a type III ANOVA was performed to assess the significance of the terms and the interactions between the main factors. The calculation of partial *R* squared for linear mixed models was performed with the package “r2glmm” (Jaeger, 2017). According to Lastrucci et al. (2017), both the site and the status have an important role in the analysis of the trends of macromorphological traits. For each macromorphological trait, relationships with the three AGD indices were analyzed with a linear mixed-effects model considering the macromorphological trait as response variable, the genetic diversity index as main factor and the status and the sites as random factors. The normality of the residuals was tested by a Shapiro–Wilk test and a type III ANOVA table to assess the significance of the terms was performed. In order to obtain the normality of residuals, when necessary, a log-transformation of data was applied.

All the analyses were performed in the R environment (R Core Team, 2017); the linear mixed-effects model was carried out with the package “lmerTest” (Kuznetsova et al., 2017) and the results were displayed through the package “effects” (Fox, 2003).

## Results

### trnT-trnL/rbcL-psaI Regions Analysis

**FIGURE 2:**
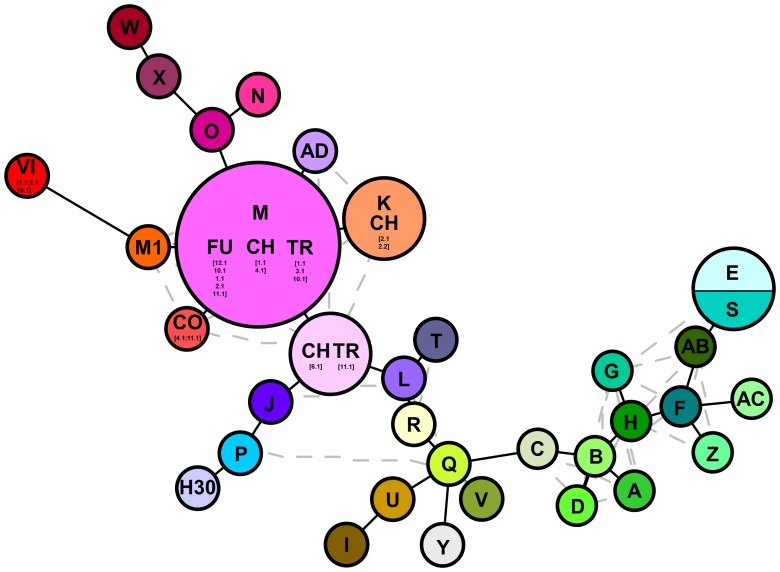
Haplotype networks analysis based on cpDNA markers (trnT-trnL/rbcL-psaI). Letters denote haplotypes as reported in Supplementary Table S1; gray lines represent alternative connection separating the different haplotypes. Numbers depict the samples analyzed in this study as reported in Supplementary Table S2.

The combined trnT-trnL/rbcL-psaI region was 1986 base pairs long. There were 1125 conserved sites, 861 variable sites, 481 parsimony informative sites, and 380 singletons. The mean Kimura two-parameter genetic distances between the outgroup and ingroup accessions ranged from a minimum of 0.037 to a maximum of 0.040. The number of base substitutions per site, from averaging over all the sequence pairs within each group, is shown in **Table 2**; it ranged from 0.001 for the “North America native group” and “*P. japonicus* related group,” to 0.002 for the “*P. australis* s.l. group” and “HapI/U group.” The HapI/U groups were well differentiated, with an equal genetic distance between the groups (0.005). In terms of their genetic distance, the North American lineage was equally distant from all the other groups (0.005). The haplotype network analysis (**Figure 2**) showed that the haplotype M was observed in all the accessions from ‘*Fu*’ and partially for ‘*Ch*’ and ‘*Tr.*’ In addition to haplotype M, the common reed from ‘*Ch*’ hosted accessions with sequences similar to haplotype K (CH2.1 and CH2.2). The multi-alignment of sequences showed that HapK and the accessions CH2.1 and CH2.2 were characterized by a small nucleotide subunit (32 bp long), which was repeated twice, from position 182 to 278 of region trnT-trnL (Supplementary Table S2). The samples CH6.1 and TR11.1 constituted a new haplotype (CHTR), similar to haplotypes M, from which it differed by the different organization of the region trnT-trnL from position 182 to 278 of the alignment (Supplementary Table S2). All the accessions from ‘*Co*’ represent a second new haplotype for Italy (CO), that differed from the haplotype M only by a single insertion at position 855 of the alignment (Supplementary Table S2). Finally, all the accessions from Vico represent the third new haplotype (VI) for central Italy. Al samples of Vico Lake result in the most divergent in the whole analyzed Italian samples, and show a higher percentage of variable sites with respect to the ingroup. M1 is the most similar haplotype to VI, with the latter sharing the same simple sequence repeats of G(A)8, from position 615 to position 626 of the alignment (Supplementary Table S2).

**Table 2:** Estimates of average evolutionary divergence over sequence pairs between groups (lower left matrix) and within groups (AED).

Group						AED
*P. australis* s.l. group	0.000					0.002
HapI/U group	0.005	0.000				0.002
N America native group	0.005	0.005	0.000			0.001
*P. japonicus* related group	0.002	0.005	0.005	0.000		0.001
Outgroup	0.040	0.037	0.040	0.039	0.000	0.052

*Grouping affiliation codes (Group) as in **Table 1** and Supplementary Table S1. The taxa used as outgroup were reported in the main text.*

### Amplified Fragment Length Polymorphisms Analysis

The AFLP analysis used 381 samples. The selected combinations of primers produced a total of 322 loci (98% polymorphic), 148 for the combination hex_EcoRI-CTA/MseI-ATG and 174 for that of fam_EcoRI-TAC/MseI-ATG. The percentage of polymorphic loci (PPL) ranged from a maximum of 66.9 (Fu09) down to a minimum of 28.8 (Tr01). Within each population, the average percentage of polymorphic loci varied from 52.3 for ‘*Ch*’ to 36.8 for ‘*Vi.*’ No clonal individuals were detected.

### Outlier Loci Detection and Analysis of Within-Population Gene Diversity

**FIGURE 3:**
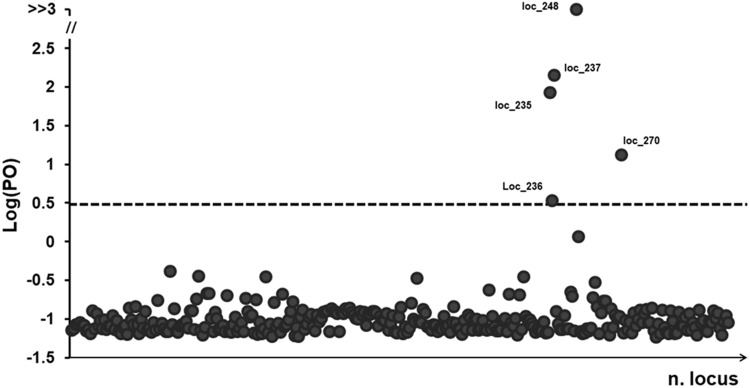
BayeScan plots of 322 (AFLP) loci in 381 samples of *P. australis*. The line is the threshold [Log (PO) = 0.5] used for identifying outlier loci. Dots that fall over to the threshold line are identified as outlier loci and the dimension in base pairs (bp) was reported.

The BayeScan analysis identified five outlier loci that had a posterior probability greater than 0.76 (at a threshold of log10 PO > 0.5; **Figure 3**), representing just 1.5% of the all analyzed loci. The highest level of high-differentiated loci found was for ‘*Ch*’ whereas the lowest was for ‘*Co.*’ Three stands apparently lacked any outlier loci (i.e., Co04, Fu10, and Tr01).

### Hierarchical Structure of Genetic Variation

**FIGURE 4:**
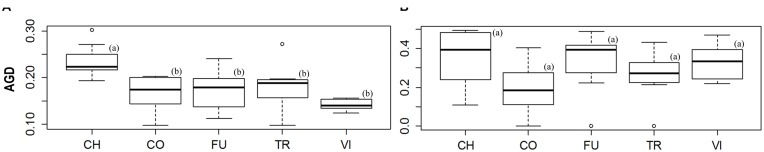
Boxplot diagrams used to illustrate the differences across sites in the levels of average gene diversity over loci (AGD). Significant differences (*P*-value < 0.05) between sites are highlighted by different letters in brackets. The diagrams **(A,B)** represent the differences of genetic diversity across sites for all and outlier loci, respectively.

The levels of AGD_all were similar among all populations (mean = 0.179). However, the population from ‘*Ch*’ had the highest levels of AGD_all and AGD_neut, differing significantly from the other four sites (**Figure 4A**). No significant differences were found among the five wetlands for the values of AGD_out (**Figure 4B**). The lowest AGD value was observed for ‘*Vi*’ for the all loci, whereas the ‘*Co*’ showed the AGD lowest value for the outlier loci (**Figure 4B** and Supplementary Table S4). No correlations were found among the AGD_all, AGD_neut and AGD_out indices and the two ecological status indices (emerged; flooded).

**FIGURE 5:**
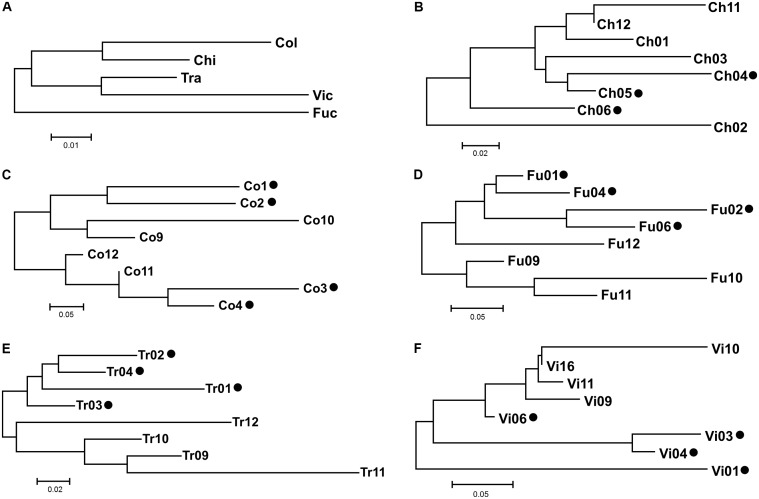
Neighbor-Joining dendrograms based on Slatkin’s linearized *F*_st_ matrix. The codes of sampling sites were reported as in **Table 1**. Scale bar indicates Slatkin’s linearized *F*_st_ value of genetic distance. **(A)** The genetic distance among the five population surveyed. The dendrograms from **(B–F)** indicate the genetic distance among the sampling sites within a population. Black dots indicate the flooded status of the sampling sites.

The AMOVA showed that the greatest amount of variation occurred at the intra-sampling site level (66.28%), rather than among the sampling sites (33.72%; **Table 3**). Among all the hypothetical groupings, the partition of the sites based on their provenance with Ch2 and Ch6 + Tr11 isolated from each other, accounted for the highest percentage of among-group variation (9.51%). This indicates that each site was a plausible genetic center of propagation and that Ch2, Ch6, Tr11 were different haplotypes within the respective populations (**Table 4**). The genetic differentiation among the five populations was relatively high (*F*_st_ = 0.337) and the estimated effective number of migrants was less than one (*N*_m_ = 0.497), suggesting restricted gene flow or low effective population size. Instead, the genetic differentiation among groups within populations, decreased significantly (*F*_st_ = 0.095), and the *N*_m_ was 2.371, suggesting increased gene flow within each population, and is expected to retain genetic connectivity (Whitlock and McCauley, 1999). According to the neighbor-joining dendrogram based on the genetic distance among populations, the ‘*Fu*’ population was the most isolated from the other four sites, with the latter divided into two clades, one formed by ‘*Ch*’ and ‘*Co*’ and the other formed by ‘*Tr*’ and ‘*Vi*’ (**Figure 5A**). Each lake had different levels of genetic organization: ‘*Fu*,’ ‘*Tr*,’ and ‘*Vi*’s distinguished the flooded and emerged stands (**Figures 5D–F**). By contrast, the flooded stands from the ‘*Co*’ separated into two different terminal clades and were merged with the emerged accessions (**Figure 5C**). The samples Ch02 and Ch06 were the most divergent among the accessions of ‘*Ch.*’ Two emerged samples, Ch11 and Ch12, were clustered with the Ch01, whereas the remaining flooded samples of Ch04 and Ch05 were clustered with Ch03 (**Figure 5B**). Statistical support for the genetic distances are reported in Supplementary Table S5.

**Table 3:** Partition of genetic variance.

Source of variation	*df*	Sum of squares	Variance components	Percentage of variation	*P*-values
Among populations	39	5874.139	14.57659	33.72	<0.0001
Within populations	311	8910.465	28.65101	66.28	<0.0001
Total	350	14784.604	43.22760	100	

*AMOVA was performed at two hierarchical levels testing the differentiation among 381 P. australis samples from five populations. The table shows: degrees of freedom (df), sum of squared deviations, variance component estimates, percentages of total variance contributed by each component, and the probability of obtaining a more extreme component estimate by chance alone (p). P-values were estimated with 1023 permutations*.

**Table 4:** Partition of genetic variance among groups of populations.

Grouping	*df*	Sum of squares	Variance components	*F*_CT_	Percentage of variation	*P*-values
Among wetlands						
(*Ch*)-(*Co*)-(*Fu*)-(*Tr*)-(*Vi*)	4	1463.394	3.41121	0.07900	7.90	<0.0001
Among haplotypes						
(*Ch*+*Co*+*Fu*+*Tr*)-(*Vi*)-(Ch2)-(Ch6+Tr11)	3	704.742	1.96648	0.04504	4.50	<0.0100
Among wetlands plus haplotypes						
(*Ch*)-(*Co*)-(*Fu*)-(*Tr*)-(*Vi*)-(Ch2)-(Ch6+Tr11)	6	1879.201	4.11262	0.09514	9.51	<0.0001

*AMOVA was performed on diverse hypothetical subdivision in groups of populations. Data show the degrees of freedom (df), the sum of squared deviations, the variance component estimates, the percentage of total variance contributed by the among-group level and P-values, estimated computing 1000 permutations*.

### Elemental Characterization

The sediments of the 40 sampling plots were examined for their heavy metal contents. The trace metals Cu, Zn, Pb, Cr, Ni, and Cd, were determined by ICP-AES (summaries of this dataset are reported in **Table 5**). The concentration levels showed in the present study, were compatible with the common ranges of heavy metals in Italian soils (Cicchella et al., 2015), despite some evident differences. Specifically, Chiusi Lake and Fucecchio Marsh showed the highest values for Zn, Cr, Cu, and Ni, often differing significantly from the other sites. Colfiorito Marsh and Vico Lake often showed the lowest values of concentration for several of the analyzed elements.

**Table 5:** Concentration for trace elements (mg/kg) and standard deviation in the wetland sediments analyzed in the present work.

	*Ch*	*Co*	*Fu*	*Tr*	*Vi*	Cons I	Cons II
Cd	6.861 ± 2.334^a^	4.084 ± 1.571^a^	7.142 ± 2.671^a^	4.081 ± 0.691^a^	4.820 ± 3.221^a^	0.6	25
Pb^∗^	29.078 ± 14.936^ab^	17.805 ± 12.935^ab^	63.749 ± 47.896^b^	18.209 ± 16.131^a^	32.483 ± 12.622^ab^	25	118
Zn	121.735 ± 15.049^bc^	79.302 ± 23.534^ab^	147.746 ± 53.535^c^	62.973 ± 35.148^a^	57.755 ± 13.847^a^	146	800
Cr	140.640 ± 28.778^c^	33.988 ± 15.469^ab^	139.711 ± 24.879^c^	62.507 ± 31.425^b^	14.096 ± 7.343^a^	26	60
Ni	115.694 ± 20.743^b^	41.754 ± 20.277^a^	98.486 ± 18.024^b^	49.046 ± 28.686^a^	20.422 ± 5.776^a^	11	32
Cu	48.057 ± 16.520^c^	23.183 ± 6.696^a^	43.823 ± 15.715^bc^	25.507 ± 19.302^ab^	20.960 ± 7.509^a^	11	60

*Dataset is divided into the five main sites (Ch, Chiusi; Vi, Vico; Tr, Trasimeno; Co, Colfiorito, Fu, Fucecchio). ^∗^Post hoc results are referred to the log transformed data. Cd, Pb, Zn, Cr, Ni, and Cu values are expressed in mg/Kg. Consensus values (Cons I and II) from de Deckere et al. (2011). The different letters (a, b, c) indicate statistically significant differences between groups after ANOVA and post hoc Tukey test.*

### Correlation Analyses

The relationships between metals and components of genetic diversity were statistically assessed. Zn and Cu were significantly associated with AGD_all (*p* = 0.0232 and 0.0086, respectively). For Zn a strong interaction with the ecological status was found (*p* = 0.0063). The same relationships were observed between AGD_neut vs. Zn and AGD_neut vs. Cu (*p* = 0.0239 and 0.0078, respectively). Also in this case, for Zn a strong interaction with the ecological status was found (*p* = 0.0057).

**FIGURE 6:**
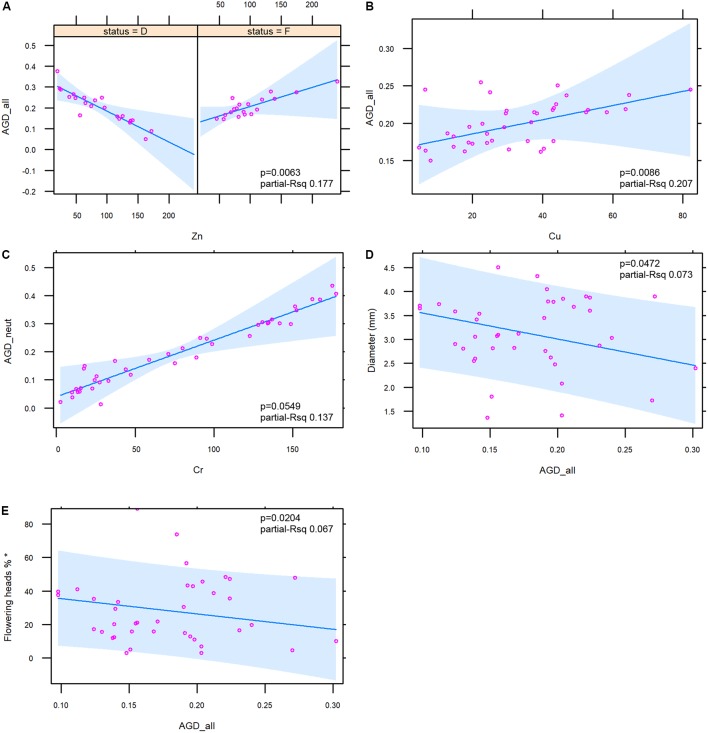
Diagrams illustrating the relationships between the genetic diversity indices and sediment metals (6 **A–C**) and macromorphological traits (6 **D,E**). For Zn, the interaction with the ecological status (D = emerged; F = flooded stands) is shown. In each figure the ANOVA *p*-value and the partial *R* squared of the presented mixed models are shown. Variable marked with ^∗^ **(E)** was log-transformed in order to obtain the normality of residuals.

No significant relationships between heavy metal concentration and AGD_out was found. The trends of the relationships between heavy metals and the two indices of genetic diversity AGD_all/AGD_neut were complex. An overall negative relationship was found between Zn and the two indices even if this metal showed a different trend according to the ecological status, with a strong negative relationship with the emerged status and a slight positive relationship with the flooded status (**Figure 6A**; figure for AGD_neut not shown). An overall positive relationship was observed between Cu and the two indices (**Figure 6B**; figure for AGD_neut not shown), and a positive relationship was observed between Cr and AGD_neut (**Figure 6C**).

As for the relationships between macromorphological traits and the genetic diversity indices, the analysis showed negative relationships between AGD_all (**Figures 6D,E**) and AGD_neut (figure not shown) with culm diameter (*p* = 0.0472 and *p* = 0.0421) and flowering percentage (*p* = 0.0204 and *p* = 0.0172). For the latter trait the log transformation was applied in order to obtain the normality of residuals. No relationships were observed between macromorphological traits and the AGD_out.

## Discussion

The present study provides the first estimates for an analysis of genetic structure of natural common reed populations as characterized by their differing health status. Much like the other Italian populations of *P. australis* (Lambertini et al., 2006, 2008), all the investigated stands were highly polyclonal and showed moderate to high levels of polymorphic loci, and medium-high levels of intra- and inter-population genetic diversity (Supplementary Table S4). This result was relatively unexpected, especially considering the marginal role that dispersed seeds and seedlings apparently have on the short-range spread of common reed, as compared to its spread via vegetative propagation (Silvertown, 2008). Indeed, it has not gone unnoticed that seedlings are common only in new open habitats, where their vitality is strongly affected by either the density of macrophyte stands (Buttery et al., 1965) or by the flooding conditions (e.g., submerged seedlings cannot survive more than 4 weeks; Mauchamp and Mésleard, 2001; Eller et al., 2017). A first explanation of the genetic structure characterizing the five analyzed populations, albeit perhaps crude and merely descriptive, comes from the survey of their haplotype composition. Insights from molecular data have generated, over the years, an increasing body of literature that provides a clearer view of the phylogeographic relationships of *P. australis* and allied taxa and their haplotypic variants, at both continental and intercontinental scales (Saltonstall, 2002; Lambertini et al., 2006). In work by Lambertini et al. (2012), both chloroplast and nuclear DNA markers were used to identify four main groups of haplotypes and several cp-microsatellite variants for *P. australis* s.l. Europe and Mediterranean regions share some of these haplotypes and, in Italy, on the basis of the analysis of Po Delta, Sardinia, and Gorgona Island (Tuscan Archipelago) populations, the most represented haplotype is M, with a lot of its variants (Lambertini et al., 2012). In the presently studied areas in Italy, the haplotype M was revealed as the most common; here, it was observed in all the accessions from Fucecchio and Colfiorito marsh and partially so for Trasimeno and Chiusi lake. However, a greater complexity in the haplotype composition of populations is demonstrated. Indeed, in addition to haplotype M, four more haplotypes were observed; one new for Italy (K) and three other (CO, CHTR, and VI), described for the first time in this work. In particular, the haplotype VI was one of the most divergent in the ingroup and ascribable to the variant of the Mediterranean haplotype M1 (Hauber et al., 2011; Lambertini et al., 2012). The M1 haplotype is referred to *P. australis* subsp. *altissimus* (Benth.) Clayton [=*P. australis* susp. *chrysanthus* (Mabille) Soják]; this taxon is rather rare in Italy, reportedly at present only in some northern regions (Conti et al., 2005; Acta Plantarum, 2016) and Sardinia (Arrigoni, 2015). The observed diversity in the trnT-trnL and rbcL-psaI regions, was corroborated by the results based on the DNA fingerprinting, which show the medium-to-high amount of genetic diversity in the natural populations of *P. australis*, with respect to their clonal structure. Also from the neighbor-joining analysis (**Figure 5**), the occurrence of diverse haplotypes of common reed from Chiusi and Trasimeno Lakes, may be deducted. Indeed the sampling sites CH2 (attributable to the haplotype K) and CH6 (attributable to the haplotype CHTR) for ‘*Ch,’* and the sampling site TR11 (ascribable to the haplotype CHTR) for ‘*Tr*’ diverged from each other within each population. Moreover, ‘*Ch’* hosted three different haplotypes (M, K, and CHTR), and had the highest mean level of genetic diversity (**Figures 4A,B**). However, despite the remarkable variability observed in terms of haplotype distribution, the AMOVA shows that only the 9.51% of the total variation among populations (33.7%) could be explained through the subdivision by geographical location and the haplotypes occurrence (**Table 4**).

### Source of Genetic Variation and RDBS Relations

The observed levels of genetic diversity could be explored and discussed in two ways. On the one hand, the high levels of genetic diversity in common reed populations may arise from repeated introductions of new germplasm (Kettenring et al., 2011). In this view, migratory routes of waterfowl, such as ducks, might represent a key factor in the long-distance dispersal of this taxon (Brochet et al., 2009). On the other hand, once stabilized, these populations might have been subjected to variable environmental and stress conditions that might have promoted outbreeding at a local scale and within-population genetic differentiation (Gigante et al., 2011, 2014; Eller et al., 2017). Indeed, the observed level of gene flow seems sufficient to retain genetic connectivity among groups of individuals within each population. It is widely accepted that indirect estimation of gene flow from *F*_st_ should be carefully interpreted. Nevertheless, Whitlock and McCauley (1999) stated that *F*_st_ may give reasonable estimates of *N*_m_ when some factors are controlled, for instance a reduced spatial scale and a large number of loci analyzed. Moreover, one of the approaches to indirectly estimate the rate of migration consists in resorting the *N*_m_ after the exclusion of outlier loci from the total analyzed (Guillot, 2011). The scarce occurrence of outlier loci in this study (only 5 out of 322) rends the *N*_m_ evaluation less prone to misinterpretations. Phenomena such as eutrophication (van der Putten, 1994), mechanical damage or grazing by animals (Ostendorp, 1999), presence of heavy metals in the sediment and/or water combined with water regulation and permanent flooding (Gigante et al., 2014; Lastrucci et al., 2016, 2017), or even phenomena such as allelopathy or phytotoxic effects from the biotic components (Schröder, 1987; Máthé et al., 2009), may have a crucial role to play in the population dynamics of common reed. For instance, as proposed by Kettenring et al. (2011), eutrophication can promote dispersal in *P. australis* by increasing seed production, and seedling abundance and growth: together, they increase the likelihood of recruitment from seeds that reach suitable habitats, which over time should promote gene diversity. Under stressful conditions, particularly those related to permanent flooding exposure, some permanently submerged Italian *P. australis* stands, affected by RDBS, showed a rate of viable seeds higher than the vigorous stands that were only temporarily submerged (Reale et al., 2011). However, this work here revealed that the intra-population genetic diversity was similar between the submerged and emerged stands. Although the ecological status of *P. australis* did not seem to influence its levels of within-populations genetic diversity, differences of the AFLP profiles were detected in terms of genetic distance. The neighbor-joining analysis shows the partial separation between the submerged and emerged plots (**Figure 5**), thus indicating a similar per-lake fingerprinting profile, likely related to the local wetland ecology of the site. These results are in line with the observations of Engloner et al. (2010) for some common reed stands in Hungary. The current study attribute the decreased number of genotypes related to water depth, to a genotype-specific tolerance for the flooding condition. These outcomes are noteworthy because of the recent findings that indicate a well-supported relationship between reed die-back and flooded conditions (Gigante et al., 2011, 2014; Lastrucci et al., 2016, 2017). Thus, understanding the variation in the AFLP profile, in particular for the DNA loci outside the range of the expected distribution compared with that of neutral loci within a sample, provides one way to identify genomic regions possibly associated with convergent or divergent selection (Yang et al., 2016). In this manner, the analysis for the outlier loci may represent a marker for *P. australis* adaptation to changes in local environmental factors and, in this present study, it could be considered as proxy indicators of the selective pressure in populations under RDBS. In the current study, the low occurrence of outliers (1.5% of the total) compared to neutral loci, suggests there is negligible evidence for divergent selection. In addition, there was no find any significant correlations between the frequency of outlier loci and ecology or macro-morphological indicators of RDBS. In support of the low effect of genetic drift among the populations, the AMOVA showed that most of the genetic variation occurs within the populations (66.28%) rather than between populations. The observed levels of molecular variance are typical for perennial, long-lived, outcrossing plant (Nybom and Bartish, 2000), and consistent with other studies of the phylogeographic relationships of local common reed stands (Lambertini et al., 2008).

Concerning the relationships among RDBS typical traits and genetic diversity, the results of this work definitely show that the decline syndrome of common reed is neither statistically related to low genetic diversity, nor to specific haplotype compositions. In addition the genetic diversity was inversely related to the decrease of diameter and flowering heads of common reed (**Figures 6D,E**), two of the trends associated with the die-back syndrome (Lastrucci et al., 2017). Hence, the level of genetic diversity seems be locally affected by biotic and/or abiotic stress factors and the present study points the attention to a possible key role played by the heavy metals and their concentrations in the rhizosphere. The contamination levels of freshwater sediment were discussed in the framework of the available sediment quality guidelines (SQG) following de Deckere et al. (2011). The current study proposed two consensus values (**Table 5**) as short and long-term objectives for sediment remediation policies based on Sediment Effect Concentrations (SECs). The consensus I values were close to the “non-effect” of SQG and correspond to the averages between Lowest Effect Levels and Threshold Effect Level (de Deckere et al., 2011). Whereas the consensus II values represent the average of Severe Effect Levels and Probable Effect Levels (de Deckere et al., 2011), therefore describing a situation of probable toxic effects. The five wetlands considered in the present work show heavy metals concentrations that range from relatively natural or unpolluted (‘*Co*’ and ‘*Vi*’) to moderately (anthropogenic) polluted (‘*Tr*,’ ‘*Ch*,’ and ‘*Fu*’). In particular the mean values for Cr and Ni were above the consensus II value for the ‘*Ch*,’ ‘*Tr*,’ ‘*Co*,’ and ‘*Fu*’ sites. The mean values for Cd and Cu were in the range between the consensus I and II value to all the sites. The mean values for Zn and Pb were below the consensus I value for ‘*Ch*,’ ‘*Vi*,’ ‘*Tr*,’ and ‘*Co.*’ Considering the overall mean concentrations of the heavy metals the ‘*Fu*’ wetland was the most polluted followed by ‘*Ch*,’ ‘*Tr*,’ ‘*Co*,’ and ‘*Vi.*’ Many *in vivo* and *in vitro* studies demonstrated that a high concentration of chemicals could produce the phenomena of DNA random-breaking, mutations in the target sequences of the restriction enzymes (Labra et al., 2003) and oxidative DNA base damage (Beyersmann and Hartwig, 2008; Ghiani et al., 2014), which then contributes to the cryptic intra-population variability of plants (Słomka et al., 2011). Indeed, the current study showed a statistically supported correlation between the total concentration of metals (Zn, Cr, and Cu) in the rhizosphere and the average gene diversity of both overall and neutral loci of *P. australis*. Previous studies have shown that the concentration of elements (e.g., Cd, Hg, Pb, Zn, Cr, Ni, and Cu) in the common reed plant can increase with that of the soil or substrate (Bonanno and Lo Giudice, 2010; Marchand et al., 2010). In particular, the roots and rhizomes of common reed can accumulate heavy metals because of the large air spaces in the cortex parenchyma (Sawidis et al., 1995). In addition, Lin and Zhang (1990) indicated that some other biotic factors affect metal accumulation in the plants, such as their age and generation time. Considering that *P. australis* is a perennial macrophyte, one capable of vegetative propagation and re-sprouting annually from its rhizomes, it is thus conceivable to hypothesize that an increased genotoxic effect for each new generation occurs.

## Conclusion

The current study presents evidence for the occurrence of five different common reed haplotypes in central Italy (M, K, CO, VI, and CHTR). It must be pointed out that these all five haplotypes were found both in vigorous and in RDBS affected stands, indicating that this syndrome does not seem related with a specific haplotype composition of the population. In addition to the complex distribution of haplotypes, moderate to high levels of genetic diversity were observed. The first hypothesis that low genetic diversity contributes to reed die-back is therefore not supported. On the contrary, the genetic diversity is positively related with morphological trends that typify RDBS. The scarce occurrence of outlier loci and the absence of correlations between frequency of outlier loci and macro-morphological indicators of RDBS suggest no evidence for a selective pressure in populations under die-back, rejecting thus the second hypothesis. The internal genetic diversity of *P. australis* populations, instead seemed more closely related to the concentration of some metals in the sediments/soil, confirming the third hypothesis proposed in the current study. The genotoxic effects due to heavy metals pollution should be further tested in *P. australis*, and future experiments should be performed taking into consideration the effects over time.

## Author Contributions

AC, LL, and LR designed the work. AC, LL, MC, VF, DG, and LR collected the samples. AC, LL, RS, and DC acquired and analyzed the data. AC drafted the manuscript. LL, DC, MC, FF, BF, DG, RV, DV, RS, and LR critically revised the article. All the authors approved the version of the manuscript to be published.

## Conflict of Interest Statement

The authors declare that the research was conducted in the absence of any commercial or financial relationships that could be construed as a potential conflict of interest.
