# Supplementary material for: AFLP Approach Reveals Variability in Phragmites australis: Implications for Its Die-Back and Evidence for Genotoxic Effects
Source: Front Plant Sci. 2018 Mar 26;9:386. doi: 10.3389/fpls.2018.00386 (PMC5879127; doi:10.3389/fpls.2018.00386)
Supplement: Supplementary file 1 [file Table_1.docx]

| **ID** | **Haplotype** | **Distribution** | **Group** | **trnT-trnL** | **rbcl-psaI** |
| --- | --- | --- | --- | --- | --- |
| HapA | A | Northern America | N America native group | AF457397 | AY016332 |
| HapAA | AA | Northern America | N America native group | AF457395 | AF457392 |
| HapAB | AB | Northern America | N America native group | AY714215 | AY016333 |
| HapAC | AC | Northern America | N America native group | AY714216 | AY016333 |
| HapAD | AD | Europe, Gulf Coast | P. australis s.l. group | JF271679 | AY016335 |
| HapB | B | Northern America | N America native group | AF457398 | AY016332 |
| HapC | C | Northern America | N America native group | AF457399 | AY016332 |
| HapD | D | Northern America | N America native group | AF457402 | AY016332 |
| HapE | E | Northern America | N America native group | AY016325 | AY016333 |
| HapF | F | Northern America | N America native group | AF457395 | AY016333 |
| HapG | G | Northern America | N America native group | AF457396 | AY016333 |
| HapH | H | Northern America | N America native group | AF457398 | AY016333 |
| HapH30 | H30 | Easthern Asia | P. australis s.l. group | JF503245 | AF457382 |
| HapI | I | Northern and Southern America, Asia, Australia | HapI/U group | AY016328 | AY016334 |
| HapJ | J | Asia, Australia | P. australis s.l. group | AY016324 | AY016335 |
| HapK | K | Europe, Asia, Africa | P. australis s.l. group | AY016326 | AY016335 |
| HapL | L | Europe, Asia, Australia | P. australis s.l. group | AY016328 | AY016335 |
| HapM | M | Europe, Asia, Africa, Northern and Southern America, New Zeland | P. australis s.l. group | AY016327 | AY016335 |
| HapM1 | M1 | Mediterranean Region, Mississippi Delta | P. australis s.l. group | JF271678 | AY016335 |
| HapN | N | France | P. australis s.l. group | AF457393 | AY016335 |
| HapO | O | Europe, Asia, Australia | P. australis s.l. group | AF457394 | AY016335 |
| HapP | P | Eastern Russia, Eastern Asia, Australia | P. australis s.l. group | AY016324 | AF457382 |
| HapQ | Q | Asia, Australia | P. australis s.l. group | AY016328 | AF457382 |
| HapR | R | Africa | P. australis s.l. group | AY016328 | AF457383 |
| HapS | S | Northern America | N America native group | AY016325 | AF457384 |
| HapT | T | Europe | P. australis s.l. group | AY016328 | AF457385 |
| HapU | U | Asia, Australia | HapI/U group | AY016328 | AF457386 |
| HapV | V | Africa | P. australis s.l. group | AY016328 | AF457387 |
| HapW | W | Northeast Asia | P. japonicus related group | AF457401 | AF457388 |
| HapX | X | Asia | P. japonicus related group | AF457401 | AF457389 |
| HapY | Y | Southern America | P. australis s.l. group | AF457400 | AF457390 |
| HapZ | Z | Northern America | N America native group | AF457395 | AF457391 |

**Supplementary Table S1**. List of examined accessions of Phragmites with accession codes, haplotype classification, main geographic distributions, grouping affiliation codes for analysis of genetic distance (Group), and GenBank accessions.
