# Supplementary material for: AFLP Approach Reveals Variability in Phragmites australis: Implications for Its Die-Back and Evidence for Genotoxic Effects
Source: Front Plant Sci. 2018 Mar 26;9:386. doi: 10.3389/fpls.2018.00386 (PMC5879127; doi:10.3389/fpls.2018.00386)
Supplement: Supplementary file 2 [file Table_2.pdf]

Supplementary Table S2: Multiple alignments for 53 concatenated sequences of the intergenic spacer regions trnT-trnL+rbcL-psaI. The codes of sampling sites were reported as in Tab. 1.

```
                    ....|....| ....|....| ....|....| ....|....| ....|....| ....|....| ....|....| ....|....| ....|....| ....|....|
                        5          15         25         35         45         55         65         75         85         95

HapM                CTAGTTCGAA ATTTGAAGTT CTACT----- ---------- ---------- ---------- ---------- ---------- ---------- ----------
HapM1               CTAGTTCGAA ATTTGAAGTT CTACT----- ---------- ---------- ---------- ---------- ---------- ---------- ----------
FU12.1              CTAGTTCGAA ATTTGAAGTT CTACT----- ---------- ---------- ---------- ---------- ---------- ---------- ----------
FU10.1              CTAGTTCGAA ATTTGAAGTT CTACT----- ---------- ---------- ---------- ---------- ---------- ---------- ----------
FU1.1               CTAGTTCGAA ATTTGAAGTT CTACT----- ---------- ---------- ---------- ---------- ---------- ---------- ----------
FU2.1               CTAGTTCGAA ATTTGAAGTT CTACT----- ---------- ---------- ---------- ---------- ---------- ---------- ----------
FU11.1              CTAGTTCGAA ATTTGAAGTT CTACT----- ---------- ---------- ---------- ---------- ---------- ---------- ----------
CO4.1               CTAGTTCGAA ATTTGAAGTT CTACT----- ---------- ---------- ---------- ---------- ---------- ---------- ----------
CO11.1              CTAGTTCGAA ATTTGAAGTT CTACT----- ---------- ---------- ---------- ---------- ---------- ---------- ----------
TR1.1               CTAGTTCGAA ATTTGAAGTT CTACT----- ---------- ---------- ---------- ---------- ---------- ---------- ----------
TR3.1               CTAGTTCGAA ATTTGAAGTT CTACT----- ---------- ---------- ---------- ---------- ---------- ---------- ----------
TR10                CTAGTTCGAA ATTTGAAGTT CTACT----- ---------- ---------- ---------- ---------- ---------- ---------- ----------
TR11.1              CTAGTTCGAA ATTTGAAGTT CTACT----- ---------- ---------- ---------- ---------- ---------- ---------- ----------
Vi16.1              CTAGTTCGAA ATTTGAAGTT CTACT----- ---------- ---------- ---------- ---------- ---------- ---------- ----------
Vi1.1               CTAGTTCGAA ATTTGAAGTT CTACT----- ---------- ---------- ---------- ---------- ---------- ---------- ----------
Vi3.1               CTAGTTCGAA ATTTGAAGTT CTACT----- ---------- ---------- ---------- ---------- ---------- ---------- ----------
CH1.1               CTAGTTCGAA ATTTGAAGTT CTACT----- ---------- ---------- ---------- ---------- ---------- ---------- ----------
CH4.1               CTAGTTCGAA ATTTGAAGTT CTACT----- ---------- ---------- ---------- ---------- ---------- ---------- ----------
CH6.1               CTAGTTCGAA ATTTGAAGTT CTACT----- ---------- ---------- ---------- ---------- ---------- ---------- ----------
CH2.2               CTAGTTCGAA ATTTGAAGTT CTACT----- ---------- ---------- ---------- ---------- ---------- ---------- ----------
CH2.1               CTAGTTCGAA ATTTGAAGTT CTACT----- ---------- ---------- ---------- ---------- ---------- ---------- ----------
HapAD               CTAGTTCGAA ATTTGAAGTT CTACT----- ---------- ---------- ---------- ---------- ---------- ---------- ----------
HapO                CTAGTTCGAA ATTTGAAGTT CTACT----- ---------- ---------- ---------- ---------- ---------- ---------- ----------
HapN                CTAGTTCGAA ATTTGAAGTT CTACT----- ---------- ---------- ---------- ---------- ---------- ---------- ----------
HapK                CTAGTTCGAA ATTTGAAGTT CTACT----- ---------- ---------- ---------- ---------- ---------- ---------- ----------
HapJ                CTAGTTCGAA ATTTGAAGTT CTACT----- ---------- ---------- ---------- ---------- ---------- ---------- ----------
HapL                CTAGTTCGAA ATTTGAAGTT CTACT----- ---------- ---------- ---------- ---------- ---------- ---------- ----------
HapQ                CTAGTTCGAA ATTTGAAGTT CTACT----- ---------- ---------- ---------- ---------- ---------- ---------- ----------
HapV                CTAGTTCGAA ATTTGAAGTT CTACT----- ---------- ---------- ---------- ---------- ---------- ---------- ----------
HapR                CTAGTTCGAA ATTTGAAGTT CTACT----- ---------- ---------- ---------- ---------- ---------- ---------- ----------
HapP                CTAGTTCGAA ATTTGAAGTT CTACT----- ---------- ---------- ---------- ---------- ---------- ---------- ----------
HapU                CTAGTTCGAA ATTTGAAGTT CTACT----- ---------- ---------- ---------- ---------- ---------- ---------- ----------
HapT                CTAGTTCGAA ATTTGAAGTT CTACT----- ---------- ---------- ---------- ---------- ---------- ---------- ----------
HapAA               CTAGTTCGAA ATTTGAAGTT CTACT----- ---------- ---------- ---------- ---------- ---------- ---------- -TAGAAAATT
HapH30              CTAGTTCGAA ATTTGAAGTT CTACT----- ---------- ---------- ---------- ---------- ---------- ---------- ----------
HapI                CTAGTTCGAA ATTTGAAGTT CTACT----- ---------- ---------- ---------- ---------- ---------- ---------- ----------
HapE                CTAGTTCGAA ATTTGAAGTT CTACT----- ---------- ---------- ---------- ---------- ---------- ---------- ----------
HapAC               CTAGTTCGAA ATTTGAAGTT CTACT----- ---------- ---------- ---------- ---------- ---------- ---------- -TAGAAAATT
HapF                CTAGTTCGAA ATTTGAAGTT CTACT----- ---------- ---------- ---------- ---------- ---------- ---------- -TAGAAAATT
HapH                CTAGTTCGAA ATTTGAAGTT CTACT----- ---------- ---------- ---------- ---------- ---------- ---------- -TAGAAAATT
HapG                CTAGTTCGAA ATTTGAAGTT CTACT----- ---------- ---------- ---------- ---------- ---------- ---------- ----------
HapAB               CTAGTTCGAA ATTTGAAGTT CTACT----- ---------- ---------- ---------- ---------- ---------- ---------- ----------
HapZ                CTAGTTCGAA ATTTGAAGTT CTACT----- ---------- ---------- ---------- ---------- ---------- ---------- -TAGAAAATT
HapD                CTAGTTCGAA ATTTGAAGTT CTACT----- ---------- -------TAG AAAATTAGAA AAAAAAAAAT AGAAAATTAG AAAAAAAAAA ATAGAAAATT
HapA                CTAGTTCGAA ATTTGAAGTT CTACT----- ---------- ---------- ---------- ---------T AGAAAATTAG AAAAAAAAAA ATAGAAAATT
HapB                CTAGTTCGAA ATTTGAAGTT CTACT----- ---------- ---------- ---------- ---------- ---------- ---------- -TAGAAAATT
HapC                CTAGTTCGAA ATTTGAAGTT CTACTTAGAA AATTAGAAAA AAAAAAATAG AAAATTAGAA AAAAAAAAAT AGAAAATTAG AAAAAAAAAA ATAGAAAATT
HapY                CTAGTTCGAA ATTTGAAGTT CTACT----- ---------- ---------- ---------- ---------- ---------- ---------- ----------
HapW                CTAGTTCGAA ATTTGAAGTT CTACT----- ---------- ---------- ---------- ---------- ---------- ---------- ----------
HapX                CTAGTTCGAA ATTTGAAGTT CTACT----- ---------- ---------- ---------- ---------- ---------- ---------- ----------
HapS                CTAGTTCGAA ATTTGAAGTT CTACT----- ---------- ---------- ---------- ---------- ---------- ---------- ----------
HaKonecNC_025235    CTAGTTCGAA ATTTGAAGTT CTACT----- ---------- ---------- ---------- ---------- ---------- ---------- ----------
Monparadox          CTAGTTCGAA ATTTGAAGTT CTACT----- ---------- ---------- ---------- ---------- ---------- ---------- ----------

                    ....|....| ....|....| ....|....| ....|....| ....|....| ....|....| ....|....| ....|....| ....|....| ....|....|
                       105        115        125        135        145        155        165        175        185        195

HapM                ---------- ---------- -TAGAAAAAA AAAA-TACTA GAATTTTAGA AAGATAAAGT TAGCTTGATA TGCTTAACTA A--------- ----------
HapM1               ---------- ---------- -TAGAAAAAA AAAA-TACTA GAATTTTAGA AAGATAAAGT TAGCTTGATA TGCTTAACTA A--------- ----------
FU12.1              ---------- ---------- -TAGAAAAAA AAAA-TACTA GAATTTTAGA AAGATAAAGT TAGCTTGATA TGCTTAACTA A--------- ----------
FU10.1              ---------- ---------- -TAGAAAAAA AAAA-TACTA GAATTTTAGA AAGATAAAGT TAGCTTGATA TGCTTAACTA A--------- ----------
FU1.1               ---------- ---------- -TAGAAAAAA AAAA-TACTA GAATTTTAGA AAGATAAAGT TAGCTTGATA TGCTTAACTA A--------- ----------
FU2.1               ---------- ---------- -TAGAAAAAA AAAA-TACTA GAATTTTAGA AAGATAAAGT TAGCTTGATA TGCTTAACTA A--------- ----------
FU11.1              ---------- ---------- -TAGAAAAAA AAAA-TACTA GAATTTTAGA AAGATAAAGT TAGCTTGATA TGCTTAACTA A--------- ----------
CO4.1               ---------- ---------- -TAGAAAAAA AAAA-TACTA GAATTTTAGA AAGATAAAGT TAGCTTGATA TGCTTAACTA A--------- ----------
CO11.1              ---------- ---------- -TAGAAAAAA AAAA-TACTA GAATTTTAGA AAGATAAAGT TAGCTTGATA TGCTTAACTA A--------- ----------
TR1.1               ---------- ---------- -TAGAAAAAA AAAA-TACTA GAATTTTAGA AAGATAAAGT TAGCTTGATA TGCTTAACTA A--------- ----------
TR3.1               ---------- ---------- -TAGAAAAAA AAAA-TACTA GAATTTTAGA AAGATAAAGT TAGCTTGATA TGCTTAACTA A--------- ----------
TR10.1              ---------- ---------- -TAGAAAAAA AAAA-TACTA GAATTTTAGA AAGATAAAGT TAGCTTGATA TGCTTAACTA A--------- ----------
TR11.1              ---------- ---------- -TAGAAAAAA AAAA-TACTA GAATTTTAGA AAGATAAAGT TAGCTTGATA TGCTTAACTA A--------- ----------
Vi16.1              ---------- ---------- -TAGAAAAAA AAA--TACTA GAATTTTAGA AAGATAAAGT TAGCTTGATA TGCTTAACTA A--------- ----------
VI1.1               ---------- ---------- -TAGAAAAAA AAA--TACTA GAATTTTAGA AAGATAAAGT TAGCTTGATA TGCTTAACTA A--------- ----------
Vi3.1               ---------- ---------- -TAGAAAAAA AAA--TACTA GAATTTTAGA AAGATAAAGT TAGCTTGATA TGCTTAACTA A--------- ----------
CH1.1               ---------- ---------- -TAGAAAAAA AAAA-TACTA GAATTTTAGA AAGATAAAGT TAGCTTGATA TGCTTAACTA A--------- ----------
CH4.1               ---------- ---------- -TAGAAAAAA AAAA-TACTA GAATTTTAGA AAGATAAAGT TAGCTTGATA TGCTTAACTA A--------- ----------
CH6.1               ---------- ---------- -TAGAAAAAA AAAA-TACTA GAATTTTAGA AAGATAAAGT TAGCTTGATA TGCTTAACTA A--------- ----------
CH2.2               ---------- ---------- -TAGAAAAAA AAAA-TACTA GAATTTTAGA AAGATAAAGT TAGCTTGATA TGCTTAACTA AATGATATTC TTAAATAGGA
CH2.1               ---------- ---------- -TAGAAAAAA AAAA-TACTA GAATTTTAGA AAGATAAAGT TAGCTTGATA TGCTTAACTA AATGATATTC TTAAATAGGA
HapAD               ---------- ---TAGAAAA TTAGAAAAAA AAAA-TACTA GAATTTTAGA AAGATAAAGT TAGCTTGATA TGCTTAACTA A--------- ----------
HapO                ---------- ---------- -TAGAAAAAA AAAAATACTA GAATTTTAGA AAGATAAAGT TAGCTTGATA TGCTTAACTA A--------- ----------
HapN                ---------- ---------- -TAGAAAAAA AAAAATACTA GAATTTTAGA AAGATAAAGT TAGCTTGATA TGCTTAACTA A--------- ----------
HapK                ---------- ---------- -TAGAAAAAA AAAA-TACTA GAATTTTAGA AAGATAAAGT TAGCTTGATA TGCTTAACTA AATGATATTC TTAAATAGGA
HapJ                ---------- ---------- -TAGAAAAAA AAAA-TACTA GAATTTTAGA AAGATAAAGT TAGCTTGATA TGCTTAACTA A--------- ----------
HapL                ---------- ---------- -TAGAAAAAA AAAAATACTA GAATTTTAGA AAGATAAAGT TAGCTTGATA TGCTTAACTA A--------- ----------
HapQ                ---------- ---------- -TAGAAAAAA AAAAATACTA GAATTTTAGA AAGATAAAGT TAGCTTGATA TGCTTAACTA A--------- ----------
HapV                ---------- ---------- -TAGAAAAAA AAAAATACTA GAATTTTAGA AAGATAAAGT TAGCTTGATA TGCTTAACTA A--------- ----------
HapR                ---------- ---------- -TAGAAAAAA AAAAATACTA GAATTTTAGA AAGATAAAGT TAGCTTGATA TGCTTAACTA A--------- ----------
HapP                ---------- ---------- -TAGAAAAAA AAAA-TACTA GAATTTTAGA AAGATAAAGT TAGCTTGATA TGCTTAACTA A--------- ----------
HapU                ---------- ---------- -TAGAAAAAA AAAAATACTA GAATTTTAGA AAGATAAAGT TAGCTTGATA TGCTTAACTA A--------- ----------
HapT                ---------- ---------- -TAGAAAAAA AAAAATACTA GAATTTTAGA AAGATAAAGT TAGCTTGATA TGCTTAACTA A--------- ----------
HapAA               AGAAAAAAAA AAATAGAAAA TTAGAAAAAA AAAAATACTA GAATTTTAGA AAGATAAAGT TAGCTTGATA TGCTTAACTA A--------- ----------
HapH30              ---------- ---TAGAAAA TTAGAAAAAA AAAA-TACTA GAATTTTAGA AAGATAAAGT TAGCTTGATA TGCTTAACTA A--------- ----------
HapI                ---------- ---------- -TAGAAAAAA AAAAATACTA GAATTTTAGA AAGATAAAGT TAGCTTGATA TGCTTAACTA A--------- ----------
HapE                ---------- ---TAGAAAA TTAGAAAAAA AAAAATACTA GAATTTTAGA AAGATAAAGT TAGCTTGATA TGCTTAACTA A--------- ----------
HapAC               AGAAAAAAAA AAATAGAAAA TTAGAAAAAA AAAAATACTA GAATTTTAGA AAGATAAAGT TAGCTTGATA TGCTTAACTA A--------- ----------
HapF                AGAAAAAAAA AAATAGAAAA TTAGAAAAAA AAAAATACTA GAATTTTAGA AAGATAAAGT TAGCTTGATA TGCTTAACTA A--------- ----------
HapH                AGAAAAAAAA AAATAGAAAA TTAGAAAAAA AAAAATACTA GAATTTTAGA AAGATAAAGT TAGCTTGATA TGCTTAACTA A--------- ----------
HapG                ---------- ---TAGAAAA TTAGAAAAAA AAAAATACTA GAATTTTAGA AAGATAAAGT TAGCTTGATA TGCTTAACTA A--------- ----------
HapAB               ---------- ---TAGAAAA TTAGAAAAAA AAAAATACTA GAATTTTAGA AAGATAAAGT TAGCTTGATA TGCTTAACTA A--------- ----------
HapZ                AGAAAAAAAA AAATAGAAAA TTAGAAAAAA AAAAATACTA GAATTTTAGA AAGATAAAGT TAGCTTGATA TGCTTAACTA A--------- ----------
HapD                AGAAAAAAAA AAATAGAAAA TTAGAAAAAA AAAAATACTA GAATTTTAGA AAGATAAAGT TAGCTTGATA TGCTTAACTA A--------- ----------
HapA                AGAAAAAAAA AAATAGAAAA TTAGAAAAAA AAAAATACTA GAATTTTAGA AAGATAAAGT TAGCTTGATA TGCTTAACTA A--------- ----------
HapB                AGAAAAAAAA AAATAGAAAA TTAGAAAAAA AAAAATACTA GAATTTTAGA AAGATAAAGT TAGCTTGATA TGCTTAACTA A--------- ----------
HapC                AGAAAAAAAA AAATAGAAAA TTAGAAAAAA AAAAATACTA GAATTTTAGA AAGATAAAGT TAGCTTGATA TGCTTAACTA A--------- ----------
HapY                ---------- ---TAGAAAA TTAGAAAAAA AAAAATACTA GAATTTTAGA AAGATAAAGT TAGCTTGATA TGCTTAACTA A--------- ----------
HapW                ---------- ---------- -TAGAAAAAA AAAAATACTA GAATTTTAGA AAGATAAAGT TAGCTTGATA TGCTTAACTA A--------- ----------
HapX                ---------- ---------- -TAGAAAAAA AAAAATACTA GAATTTTAGA AAGATAAAGT TAGCTTGATA TGCTTAACTA A--------- ----------
HapS                ---------- ---TAGAAAA TTAGAAAAAA AAAAATACTA GAATTTTAGA AAGATAAAGT TAGCTTGATA TGCTTAACTA A--------- ----------
HaKonecNC_025235    ---------- ---------- -TAGAAAAAA AAAA-TACTA GAATTTTAGA AAGATAAAGT TAGCTTGATA TGCTTAACTA A--------- ----------
Monparadoxtrnt      ---------- ---------- -TAGAAAAAC AAAC-T-CTA --ATTTCATA AAGATAAAGT TAGCTTTATA TGCTTAACTA A--------- ----------

                    ....|....| ....|....| ....|....| ....|....| ....|....| ....|....| ....|....| ....|....| ....|....| ....|....|
                       205        215        225        235        245        255        265        275        285        295

HapM                ---------- ----ATGATA TTCTTAAATA GGATTATAGA ATTTATTATG ATATTCTTAA ATAGGATTAT AGAATTTATT GAACTTTCTT TTTATTTCTC
HapM1               ---------- ----ATGATA TTCTTAAATA GGATTATAGA ATTTATTATG ATATTCTTAA ATAGGATTAT AGAATTTATT GAACTTTCTT TTTATTTCTC
FU12.               ---------- ----ATGATA TTCTTAAATA GGATTATAGA ATTTATTATG ATATTCTTAA ATAGGATTAT AGAATTTATT GAACTTTCTT TTTATTTCTC
FU10.               ---------- ----ATGATA TTCTTAAATA GGATTATAGA ATTTATTATG ATATTCTTAA ATAGGATTAT AGAATTTATT GAACTTTCTT TTTATTTCTC
FU1.1               ---------- ----ATGATA TTCTTAAATA GGATTATAGA ATTTATTATG ATATTCTTAA ATAGGATTAT AGAATTTATT GAACTTTCTT TTTATTTCTC
FU2.1               ---------- ----ATGATA TTCTTAAATA GGATTATAGA ATTTATTATG ATATTCTTAA ATAGGATTAT AGAATTTATT GAACTTTCTT TTTATTTCTC
FU11.               ---------- ----ATGATA TTCTTAAATA GGATTATAGA ATTTATTATG ATATTCTTAA ATAGGATTAT AGAATTTATT GAACTTTCTT TTTATTTCTC
CO4.1               ---------- ----ATGATA TTCTTAAATA GGATTATAGA ATTTATTATG ATATTCTTAA ATAGGATTAT AGAATTTATT GAACTTTCTT TTTATTTCTC
CO11.               ---------- ----ATGATA TTCTTAAATA GGATTATAGA ATTTATTATG ATATTCTTAA ATAGGATTAT AGAATTTATT GAACTTTCTT TTTATTTCTC
TR1.1               ---------- ----ATGATA TTCTTAAATA GGATTATAGA ATTTATTATG ATATTCTTAA ATAGGATTAT AGAATTTATT GAACTTTCTT TTTATTTCTC
TR3.1               ---------- ----ATGATA TTCTTAAATA GGATTATAGA ATTTATTATG ATATTCTTAA ATAGGATTAT AGAATTTATT GAACTTTCTT TTTATTTCTC
TR10.1              ---------- ----ATGATA TTCTTAAATA GGATTATAGA ATTTATTATG ATATTCTTAA ATAGGATTAT AGAATTTATT GAACTTTCTT TTTATTTCTC
TR11.1              ---------- ---------- ---------- ---------- -------ATG ATATTCTTAA ATAGGATTAT AGAATTTATT GAACTTTCTT TTTATTTCTC
Vi16.1              ---------- ----ATGATA TTCTTAAATA GGATTATAGA ATTTATTATG ATATTCTTAA ATAGGATTAT AGAATTTATT GAACTTTCTT TTTATTTCTC
VI1.1               ---------- ----ATGATA TTCTTAAATA GGATTATAGA ATTTATTATG ATATTCTTAA ATAGGATTAT AGAATTTATT GAACTTTCTT TTTATTTCTC
Vi3.1               ---------- ----ATGATA TTCTTAAATA GGATTATAGA ATTTATTATG ATATTCTTAA ATAGGATTAT AGAATTTATT GAACTTTCTT TTTATTTCTC
CH1.1               ---------- ----ATGATA TTCTTAAATA GGATTATAGA ATTTATTATG ATATTCTTAA ATAGGATTAT AGAATTTATT GAACTTTCTT TTTATTTCTC
CH4.1               ---------- ----ATGATA TTCTTAAATA GGATTATAGA ATTTATTATG ATATTCTTAA ATAGGATTAT AGAATTTATT GAACTTTCTT TTTATTTCTC
CH6.1               ---------- ---------- ---------- ---------- -------ATG ATATTCTTAA ATAGGATTAT AGAATTTATT GAACTTTCTT TTTATTTCTC
CH2.2               TTATAGAATT TATTATGATA TTCTTAAATA GGATTATAGA ATTTATTATG ATATTCTTAA ATAGGATTAT AGAATTTATT GAACTTTCTT TTTATTTCTC
CH2.1               TTATAGAATT TATTATGATA TTCTTAAATA GGATTATAGA ATTTATTATG ATATTCTTAA ATAGGATTAT AGAATTTATT GAACTTTCTT TTTATTTCTC
HapAD               ---------- ----ATGATA TTCTTAAATA GGATTATAGA ATTTATTATG ATATTCTTAA ATAGGATTAT AGAATTTATT GAACTTTCTT TTTATTTCTC
HapO                ---------- ----ATGATA TTCTTAAATA GGATTATAGA ATTTATTATG ATATTCTTAA ATAGGATTAT AGAATTTATT GAACTTTCTT TTTATTTCTC
HapN                ---------- ----ATGATA TTCTTAAATA GGATTATAGA ATTTATTATG ATATTCTTAA ATAGGATTAT AGAATTTATT GAACTTTCTT TTTATTTCTC
HapK                TTATAGAATT TATTATGATA TTCTTAAATA GGATTATAGA ATTTATTATG ATATTCTTAA ATAGGATTAT AGAATTTATT GAACTTTCTT TTTATTTCTC
HapJ                ---------- ---------- ---------- ---------- -------ATG ATATTCTTAA ATAGGATTAT AGAATTTATT GAACTTTCTT TTTATTTCTC
HapL                ---------- ---------- ---------- ---------- -------ATG ATATTCTTAA ATAGGATTAT AGAATTTATT GAACTTTCTT TTTATTTCTC
HapQ                ---------- ---------- ---------- ---------- -------ATG ATATTCTTAA ATAGGATTAT AGAATTTATT GAACTTTCTT TTTATTTCTC
HapV                ---------- ---------- ---------- ---------- -------ATG ATATTCTTAA ATAGGATTAT AGAATTTATT GAACTTTCTT TTTATTTCTC
HapR                ---------- ---------- ---------- ---------- -------ATG ATATTCTTAA ATAGGATTAT AGAATTTATT GAACTTTCTT TTTATTTCTC
HapP                ---------- ---------- ---------- ---------- -------ATG ATATTCTTAA ATAGGATTAT AGAATTTATT GAACTTTCTT TTTATTTCTC
HapU                ---------- ---------- ---------- ---------- -------ATG ATATTCTTAA ATAGGATTAT AGAATTTATT GAACTTTCTT TTTATTTCTC
HapT                ---------- ---------- ---------- ---------- -------ATG ATATTCTTAA ATAGGATTAT AGAATTTATT GAACTTTCTT TTTATTTCTC
HapAA               ---------- ----ATGATA TTCTTAAATA GGATTATAGA ATTTATTATG ATATTCTTAA ATAGGATTAT AGAATTTATT GAACTTTCTT TTTATTTCTC
HapH30              ---------- ---------- ---------- ---------- -------ATG ATATTCTTAA ATAGGATTAT AGAATTTATT GAACTTTCTT TTTATTTCTC
HapI                ---------- ---------- ---------- ---------- -------ATG ATATTCTTAA ATAGGATTAT AGAATTTATT GAACTTTCTT TTTATTTCTC
HapE                ---------- ---------- ---------- ---------- -------ATG ATATTCTTAA ATAGGATTAT AGAATTTATT GAACTTTCTT TTTATTTCTC
HapAC               ---------- ----ATGATA TTCTTAAATA GGATTATAGA ATTTATTATG ATATTCTTAA ATAGGATTAT AGAATTTATT GAACTTTCTT TTTATTTCTC
HapF                ---------- ----ATGATA TTCTTAAATA GGATTATAGA ATTTATTATG ATATTCTTAA ATAGGATTAT AGAATTTATT GAACTTTCTT TTTATTTCTC
HapH                ---------- ----ATGATA TTCTTAAATA GGATTATAGA ATTTATTATG ATATTCTTAA ATAGGATTAT AGAATTTATT GAACTTTCTT TTTATTTCTC
HapG                ---------- ----ATGATA TTCTTAAATA GGATTATAGA ATTTATTATG ATATTCTTAA ATAGGATTAT AGAATTTATT GAACTTTCTT TTTATTTCTC
HapAB               ---------- ----ATGATA TTCTTAAATA GGATTATAGA ATTTATTATG ATATTCTTAA ATAGGATTAT AGAATTTATT GAACTTTCTT TTTATTTCTC
HapZ                ---------- ----ATGATA TTCTTAAATA GGATTATAGA ATTTATTATG ATATTCTTAA ATAGGATTAT AGAATTTATT GAACTTTCTT TTTATTTCTC
HapD                ---------- ----ATGATA TTCTTAAATA GGATTATAGA ATTTATTATG ATATTCTTAA ATAGGATTAT AGAATTTATT GAACTTTCTT TTTATTTCTC
HapA                ---------- ----ATGATA TTCTTAAATA GGATTATAGA ATTTATTATG ATATTCTTAA ATAGGATTAT AGAATTTATT GAACTTTCTT TTTATTTCTC
HapB                ---------- ----ATGATA TTCTTAAATA GGATTATAGA ATTTATTATG ATATTCTTAA ATAGGATTAT AGAATTTATT GAACTTTCTT TTTATTTCTC
HapC                ---------- ---------- ---------- ---------- -------ATG ATATTCTTAA ATAGGATTAT AGAATTTATT GAACTTTCTT TTTATTTCTC
HapY                ---------- ---------- ---------- ---------- -------ATG ATATTCTTAA ATAGGATTAT AGAATTTATT GAACTTTCTT TTTATTTCTC
HapW                ---------- ----ATGATA TTCTTAAATA GGATTATAGA ATTTATTATG ATATTCTTAA ATAGGATTAT AGAATTTATT GAACTTTCTT TTTATTTCTC
HapX                ---------- ----ATGATA TTCTTAAATA GGATTATAGA ATTTATTATG ATATTCTTAA ATAGGATTAT AGAATTTATT GAACTTTCTT TTTATTTCTC
HapS                ---------- ---------- ---------- ---------- -------ATG ATATTCTTAA ATAGGATTAT AGAATTTATT GAACTTTCTT TTTATTTCTC
HaKonecNC_025235    ---------- ---------- ---------- ---------- -------ATG ATATTCTTAA ATAGGATTAT AGAATTTATT GAACTTTCTT TTTATTTCTC
Monparadoxtrnt      ---------- ---------- ---------- ---------- -------ATG ATATTTTGAA ATAGGATTAT AGAATTAATT GAACTTTCTT TTTATTTCTC
```

```
                    ....|....| ....|....| ....|....| ....|....| ....|....| ....|....| ....|....| ....|....| ....|....| ....|....|
                         305        315        325        335        345        355        365        375        385        395

HapM                TAATTCGCAA ATCCATTTTT CTAATAGAAT CTATTCCAAT TTCTATA--- ---TTAAATT TGATTTCAGA TATTTTCAAT TTGATATGGC TCGGACGAAT
HapM1               TAATTCGCAA ATCCATTTTT CTAATAGAAT CTATTCCAAT TTCTATA--- ---TTAAATT TGATTTCAGA TATTTTCAAT TTGATATGGC TCGGACGAAT
FU12.1              TAATTCGCAA ATCCATTTTT CTAATAGAAT CTATTCCAAT TTCTATA--- ---TTAAATT TGATTTCAGA TATTTTCAAT TTGATATGGC TCGGACGAAT
FU10.1              TAATTCGCAA ATCCATTTTT CTAATAGAAT CTATTCCAAT TTCTATA--- ---TTAAATT TGATTTCAGA TATTTTCAAT TTGATATGGC TCGGACGAAT
FU1.1               TAATTCGCAA ATCCATTTTT CTAATAGAAT CTATTCCAAT TTCTATA--- ---TTAAATT TGATTTCAGA TATTTTCAAT TTGATATGGC TCGGACGAAT
FU2.1               TAATTCGCAA ATCCATTTTT CTAATAGAAT CTATTCCAAT TTCTATA--- ---TTAAATT TGATTTCAGA TATTTTCAAT TTGATATGGC TCGGACGAAT
FU11.1              TAATTCGCAA ATCCATTTTT CTAATAGAAT CTATTCCAAT TTCTATA--- ---TTAAATT TGATTTCAGA TATTTTCAAT TTGATATGGC TCGGACGAAT
CO4.1               TAATTCGCAA ATCCATTTTT CTAATAGAAT CTATTCCAAT TTCTATA--- ---TTAAATT TGATTTCAGA TATTTTCAAT TTGATATGGC TCGGACGAAT
CO11.1              TAATTCGCAA ATCCATTTTT CTAATAGAAT CTATTCCAAT TTCTATA--- ---TTAAATT TGATTTCAGA TATTTTCAAT TTGATATGGC TCGGACGAAT
TR1.1               TAATTCGCAA ATCCATTTTT CTAATAGAAT CTATTCCAAT TTCTATA--- ---TTAAATT TGATTTCAGA TATTTTCAAT TTGATATGGC TCGGACGAAT
TR3.1               TAATTCGCAA ATCCATTTTT CTAATAGAAT CTATTCCAAT TTCTATA--- ---TTAAATT TGATTTCAGA TATTTTCAAT TTGATATGGC TCGGACGAAT
TR10.1              TAATTCGCAA ATCCATTTTT CTAATAGAAT CTATTCCAAT TTCTATA--- ---TTAAATT TGATTTCAGA TATTTTCAAT TTGATATGGC TCGGACGAAT
TR11.1              TAATTCGCAA ATCCATTTTT CTAATAGAAT CTATTCCAAT TTCTATA--- ---TTAAATT TGATTTCAGA TATTTTCAAT TTGATATGGC TCGGACGAAT
Vi16.1              TAATTCGCAA ATCCATTTTT CTAATAGAAT CTATTCCAAT TTCTATA--- ---TTAAATT TGATTTCAGA TATTTTCAAT TTGATATGGC TCGGACGAAT
VI1.1               TAATTCGCAA ATCCATTTTT CTAATAGAAT CTATTCCAAT TTCTATA--- ---TTAAATT TGATTTCAGA TATTTTCAAT TTGATATGGC TCGGACGAAT
Vi3.1               TAATTCGCAA ATCCATTTTT CTAATAGAAT CTATTCCAAT TTCTATA--- ---TTAAATT TGATTTCAGA TATTTTCAAT TTGATATGGC TCGGACGAAT
CH1.1               TAATTCGCAA ATCCATTTTT CTAATAGAAT CTATTCCAAT TTCTATA--- ---TTAAATT TGATTTCAGA TATTTTCAAT TTGATATGGC TCGGACGAAT
CH4.1               TAATTCGCAA ATCCATTTTT CTAATAGAAT CTATTCCAAT TTCTATA--- ---TTAAATT TGATTTCAGA TATTTTCAAT TTGATATGGC TCGGACGAAT
CH6.1               TAATTCGCAA ATCCATTTTT CTAATAGAAT CTATTCCAAT TTCTATA--- ---TTAAATT TGATTTCAGA TATTTTCAAT TTGATATGGC TCGGACGAAT
CH2.2               TAATTCGCAA ATCCATTTTT CTAATAGAAT CTATTCCAAT TTCTATA--- ---TTAAATT TGATTTCAGA TATTTTCAAT TTGATATGGC TCGGACGAAT
CH2.1               TAATTCGCAA ATCCATTTTT CTAATAGAAT CTATTCCAAT TTCTATA--- ---TTAAATT TGATTTCAGA TATTTTCAAT TTGATATGGC TCGGACGAAT
HapAD               TAATTCGCAA ATCCATTTTT CTAATAGAAT CTATTCCAAT TTCTATA--- ---TTAAATT TGATTTCAGA TATTTTCAAT TTGATATGGC TCGGACGAAT
HapO                TAATTCGCAA ATCCATTTTT CTAATAGAAT CTATTCCAAT TTCTATA--- ---TTAAATT TCATTTCAGA TATTTTCAAT TTGATATGGC TCGGACGAAT
HapN                TAATTCGCAA ATCCATTTTT CTAATAGAAT CTATTCCAAT TTCTATA--- ---TTAAATT TGATTTCAGA TATTTTCAAT TTGATATGGC TCGGACGAAT
HapK                TAATTCGCAA ATCCATTTTT CTAATAGAAT CTATTCCAAT TTCTATA--- ---TTAAATT TGATTTCAGA TATTTTCAAT TTGATATGGC TCGGACGAAT
HapJ                TAATTCGCAA ATCCATTTTT CTAATAGAAT CTATTCCAAT TTCTATA--- ---TTAAATT TGATTTCAGA TATTTTCAAT TTGATATGGA TCGGACGAAT
HapL                TAATTCGCAA ATCCATTTTT CTAATAGAAT CTATTCCAAT TTCTATA--- ---TTAAATT TGATTTCAGA TATTTTCAAT TTGATATGGC TCGGACGAAT
HapQ                TAATTCGCAA ATCCATTTTT CTAATAGAAT CTATTCCAAT TTCTATA--- ---TTAAATT TGATTTCAGA TATTTTCAAT TTGATATGGC TCGGACGAAT
HapV                TAATTCGCAA ATCCATTTTT CTAATAGAAT CTATTCCAAT TTCTATA--- ---TTAAATT TGATTTCAGA TATTTTCAAT TTGATATGGC TCGGACGAAT
HapR                TAATTCGCAA ATCCATTTTT CTAATAGAAT CTATTCCAAT TTCTATA--- ---TTAAATT TGATTTCAGA TATTTTCAAT TTGATATGGC TCGGACGAAT
HapP                TAATTCGCAA ATCCATTTTT CTAATAGAAT CTATTCCAAT TTCTATA--- ---TTAAATT TGATTTCAGA TATTTTCAAT TTGATATGGA TCGGACGAAT
HapU                TAATTCGCAA ATCCATTTTT CTAATAGAAT CTATTCCAAT TTCTATA--- ---TTAAATT TGATTTCAGA TATTTTCAAT TTGATATGGC TCGGACGAAT
HapT                TAATTCGCAA ATCCATTTTT CTAATAGAAT CTATTCCAAT TTCTATA--- ---TTAAATT TGATTTCAGA TATTTTCAAT TTGATATGGC TCGGACGAAT
HapAA               TAATTCGCAA ATCCATTTTT CTAATAGAAT CTATTCCAAT TTCTATA--- ---TTAAATT TGATTTCAGA TATTTTCAAT TTGATATGGC TCGGACGAAT
HapH30              TAATTCGCAA ATCCATTTTT CTAATAGAAT CTATTCCAAT TTCTATA--- ---TTAAATT TGATTTCAGA TATTTTCAAT TTGATATGGA TCGGACGAAT
HapI                TAATTCGCAA ATCCATTTTT CTAATAGAAT CTATTCCAAT TTCTATA--- ---TTAAATT TGATTTCAGA TATTTTCAAT TTGATATGGC TCGGACGAAT
HapE                TAATTCGCAA ATCCATTTTT CTAATAGAAT CTATTCCAAT TTCTATA--- ---TTAAATT TGATTTCAGA TATTTTCAAT TTGATATGGC TCGGACGAAT
HapAC               TAATTCGCAA ATCCATTTTT CTAATAGAAT CTATTCCAAT TTCTATA--- ---TTAAATT TGATTTCAGA TATTTTCAAT TTGATATGGC TCGGACGAAT
HapF                TAATTCGCAA ATCCATTTTT CTAATAGAAT CTATTCCAAT TTCTATA--- ---TTAAATT TGATTTCAGA TATTTTCAAT TTGATATGGC TCGGACGAAT
HapH                TAATTCGCAA ATCCATTTTT CTAATAGAAT CTATTCCAAT TTCTATA--- ---TTAAATT TGATTTCAGA TATTTTCAAT TTGATATGGC TCGGACGAAT
HapG                TAATTCGCAA ATCCATTTTT CTAATAGAAT CTATTCCAAT TTCTATA--- ---TTAAATT TGATTTCAGA TATTTTCAAT TTGATATGGC TCGGACGAAT
HapAB               TAATTCGCAA ATCCATTTTT CTAATAGAAT CTATTCCAAT TTCTATA--- ---TTAAATT TGATTTCAGA TATTTTCAAT TTGATATGGC TCGGACGAAT
HapZ                TAATTCGCAA ATCCATTTTT CTAATAGAAT CTATTCCAAT TTCTATA--- ---TTAAATT TGATTTCAGA TATTTTCAAT TTGATATGGC TCGGACGAAT
HapD                TAATTCGCAA ATCCATTTTT CTAATAGAAT CTATTCCAAT TTCTATA--- ---TTAAATT TGATTTCAGA TATTTTCAAT TTGATATGGC TCGGACGAAT
HapA                TAATTCGCAA ATCCATTTTT CTAATAGAAT CTATTCCAAT TTCTATA--- ---TTAAATT TGATTTCAGA TATTTTCAAT TTGATATGGC TCGGACGAAT
HapB                TAATTCGCAA ATCCATTTTT CTAATAGAAT CTATTCCAAT TTCTATA--- ---TTAAATT TGATTTCAGA TATTTTCAAT TTGATATGGC TCGGACGAAT
HapC                TAATTCGCAA ATCCATTTTT CTAATAGAAT CTATTCCAAT TTCTATA--- ---TTAAATT TGATTTCAGA TATTTTCAAT TTGATATGGC TCGGACGAAT
HapY                TAATTCGCAA ATCCATTTTT CTAATAGAAT TTATTCCAAT TTCTATA--- ---TTAAATT TGATTTCAGA TATTTTCAAT TTGATATGGC TCGGACGAAT
HapW                TAATTCGCAA ATCCATTTTT CTAATAGAAT CTATTCCAAT TTCTATA--- ---TGAAATT TGATTTCAGA TATTTTCAAT TTGATATGGC TCGGACGAAT
HapX                TAATTCGCAA ATCCATTTTT CTAATAGAAT CTATTCCAAT TTCTATA--- ---TGAAATT TGATTTCAGA TATTTTCAAT TTGATATGGC TCGGACGAAT
HapS                TAATTCGCAA ATCCATTTTT CTAATAGAAT CTATTCCAAT TTCTATA--- ---TTAAATT TGATTTCAGA TATTTTCAAT TTGATATGGC TCGGACGAAT
HaKonecNC_025235    TAATTCGCAA ATCCATTTTT CTAATAGAAT CTATTCCAAT TTCTATA--- ---TTAAATT TGATTTCAGA TATTTTCAAT TTGATATGGC TCGGACGAAT
Monparadoxtrnt      TAATTCGCAA ATCAATTTTT CTAATAGAAT CGATTCCAAT TTCTATTCTC TATTGAA-TT GGATTTCAGA TATTTTCAAT TTTATATGGC TCGGACGAAT

                    ....|....| ....|....| ....|....| ....|....| ....|....| ....|....| ....|....| ....|....| ....|....| ....|....|
                         405        415        425        435        445        455        465        475        485        495

HapM                AAT------C TAATACATAG AAAAGAATAA TATATATGAA TAATATAATA AAGAGAAAAT GCCAATCTCT TGGCATTTTC ATTCGATCAT TATATACCAT
HapM1               AAT------C TAATACATAG AAAAGAATAA TATATATGAA TAATATAATA AAGAGAAAAT GCCAATCTCT TGGCATTTTC ATTCGATCAT TATATACCAT
FU12.1              AAT------C TAATACATAG AAAAGAATAA TATATATGAA TAATATAATA AAGAGAAAAT GCCAATCTCT TGGCATTTTC ATTCGATCAT TATATACCAT
FU10.1              AAT------C TAATACATAG AAAAGAATAA TATATATGAA TAATATAATA AAGAGAAAAT GCCAATCTCT TGGCATTTTC ATTCGATCAT TATATACCAT
FU1.1               AAT------C TAATACATAG AAAAGAATAA TATATATGAA TAATATAATA AAGAGAAAAT GCCAATCTCT TGGCATTTTC ATTCGATCAT TATATACCAT
FU2.1               AAT------C TAATACATAG AAAAGAATAA TATATATGAA TAATATAATA AAGAGAAAAT GCCAATCTCT TGGCATTTTC ATTCGATCAT TATATACCAT
FU11.1              AAT------C TAATACATAG AAAAGAATAA TATATATGAA TAATATAATA AAGAGAAAAT GCCAATCTCT TGGCATTTTC ATTCGATCAT TATATACCAT
CO4.1               AAT------C TAATACATAG AAAAGAATAA TATATATGAA TAATATAATA AAGAGAAAAT GCCAATCTCT TGGCATTTTC ATTCGATCAT TATATACCAT
CO11.1              AAT------C TAATACATAG AAAAGAATAA TATATATGAA TAATATAATA AAGAGAAAAT GCCAATCTCT TGGCATTTTC ATTCGATCAT TATATACCAT
TR1.1               AAT------C TAATACATAG AAAAGAATAA TATATATGAA TAATATAATA AAGAGAAAAT GCCAATCTCT TGGCATTTTC ATTCGATCAT TATATACCAT
TR3.1               AAT------C TAATACATAG AAAAGAATAA TATATATGAA TAATATAATA AAGAGAAAAT GCCAATCTCT TGGCATTTTC ATTCGATCAT TATATACCAT
TR10.1              AAT------C TAATACATAG AAAAGAATAA TATATATGAA TAATATAATA AAGAGAAAAT GCCAATCTCT TGGCATTTTC ATTCGATCAT TATATACCAT
TR11.1              AAT------C TAATACATAG AAAAGAATAA TATATATGAA TAATATAATA AAGAGAAAAT GCCAATCTCT TGGCATTTTC ATTCGATCAT TATATACCAT
Vi16.1              AAT------C TAATACTTAG AAAAGAATAA TAT-T-TGAA TAATATAATA AAGAGAAAAT GCCAATCTCT TGGCATTTTC ATTCGATCAT TATATACCAT
VI1.1               AAT------C TAATACTTAG AAAAGAATAA TAT-T-TGAA TAATATAATA AAGAGAAAAT GCCAATCTCT TGGCATTTTC ATTCGATCAT TATATACCAT
Vi3.1               AAT------C TAATACTTAG AAAAGAATAA TAT-T-TGAA TAATATAATA AAGAGAAAAT GCCAATCTCT TGGCATTTTC ATTCGATCAT TATATACCAT
CH1.1               AAT------C TAATACATAG AAAAGAATAA TATATATGAA TAATATAATA AAGAGAAAAT GCCAATCTCT TGGCATTTTC ATTCGATCAT TATATACCAT
CH4.1               AAT------C TAATACATAG AAAAGAATAA TATATATGAA TAATATAATA AAGAGAAAAT GCCAATCTCT TGGCATTTTC ATTCGATCAT TATATACCAT
CH6.1               AAT------C TAATACATAG AAAAGAATAA TATATATGAA TAATATAATA AAGAGAAAAT GCCAATCTCT TGGCATTTTC ATTCGATCAT TATATACCAT
CH2.2               AAT------C TAATACATAG AAAAGAATAA TATATATGAA TAATATAATA AAGAGAAAAT GCCAATCTCT TGGCATTTTC ATTCGATCAT TATATACCAT
CH2.1               AAT------C TAATACATAG AAAAGAATAA TATATATGAA TAATATAATA AAGAGAAAAT GCCAATCTCT TGGCATTTTC ATTCGATCAT TATATACCAT
HapAD               AAT------C TAATACATAG AAAAGAATAA TATATATGAA TAATATAATA AAGAGAAAAT GCCAATCTCT TGGCATTTTC ATTCGATCAT TATATACCAT
HapO                AAT------C TAATACATAG AAAAGAATAA TATATATGAA TAATATAATA AAGAGAAAAT GCCAATCTCT TGGCATTTTC ATTCGATCAT TATATACCAT
HapN                AATCTAATAC TAATACATAG AAAAGAATAA TATATATGAA TAATATAATA AAGAGAAAAT GCCAATCTCT TGGCATTTTC ATTCGATCAT TATATACCAT
HapK                AAT------C TAATACATAG AAAAGAATAA TATATATGAA TAATATAATA AAGAGAAAAT GCCAATCTCT TGGCATTTTC ATTCGATCAT TATATACCAT
HapJ                AAT------C TAATACATAG AAAAGAATAA TATATATGAA TAATATAATA AAGAGAAAAT GCCAATCTCT TGGCATTTTC ATTCGATCAT TATATACCAT
HapL                AAT------C TAATACATAG AAAAGAATAA TATATATGAA TAATATAATA AAGAGAAAAT GCCAATCTCT TGGCATTTTC ATTCGATCAT TATATACCAT
HapQ                AAT------C TAATACATAG AAAAGAATAA TATATATGAA TAATATAATA AAGAGAAAAT GCCAATCTCT TGGCATTTTC ATTCGATCAT TATATACCAT
HapV                AAT------C TAATACATAG AAAAGAATAA TATATATGAA TAATATAATA AAGAGAAAAT GCCAATCTCT TGGCATTTTC ATTCGATCAT TATATACCAT
HapR                AAT------C TAATACATAG AAAAGAATAA TATATATGAA TAATATAATA AAGAGAAAAT GCCAATCTCT TGGCATTTTC ATTCGATCAT TATATACCAT
HapP                AAT------C TAATACATAG AAAAGAATAA TATATATGAA TAATATAATA AAGAGAAAAT GCCAATCTCT TGGCATTTTC ATTCGATCAT TATATACCAT
HapU                AAT------C TAATACATAG AAAAGAATAA TATATATGAA TAATATAATA AAGAGAAAAT GCCAATCTCT TGGCATTTTC ATTCGATCAT TATATACCAT
HapT                AAT------C TAATACATAG AAAAGAATAA TATATATGAA TAATATAATA AAGAGAAAAT GCCAATCTCT TGGCATTTTC ATTCGATCAT TATATACCAT
HapAA               AAT------C TAATACATAG AAAAGAATAA TATATATGAA TAATATAATA AAGAGAAAAT GCCAATCTCT TGGCATTTTC ATTCGATCAT TATATACCAT
HapH30              AAT------C TAATACATAG AAAAGAATAA TATATATGAA TAATATAATA AAGAGAAAAT GCCAATCTCT TGGCATTTTC ATTCGATCAT TATATACCAT
HapI                AAT------C TAATACATAG AAAAGAATAA TATATATGAA TAATATAATA AAGAGAAAAT GCCAATCTCT TGGCATTTTC ATTCGATCAT TATATACCAT
HapE                AAT------C TAATACATAG AAAAGAATAA TATATATGAA TAATATAATA AAGAGAAAAT GCCAATCTCT TGGCATTTTC ATTCGATCAT TATATACCAT
HapAC               AAT------C TAATACATAG AAAAGAATAA TATATATGAA TAATATAATA AAGAGAAAAT GCCAAGAGAT TGGCATTTTC ATTCGATCAT TATATACCAT
HapF                AAT------C TAATACATAG AAAAGAATAA TATATATGAA TAATATAATA AAGAGAAAAT GCCAATCTCT TGGCATTTTC ATTCGATCAT TATATACCAT
HapH                AAT------C TAATACATAG AAAAGAATAA TATATATGAA TAATATAATA AAGAGAAAAT GCCAATCTCT TGGCATTTTC ATTCGATCAT TATATACCAT
HapG                AAT------C TAATACATAG AAAAGAATAA TATATATGAA TAATATAATA AAGAGAAAAT GCCAATCTCT TGGCATTTTC ATTCGATCAT TATATACCAT
HapAB               AAT------C TAATACATAG AAAAGAATAA TATATATGAA TAATATAATA AAGAGAAAAT GCCAATCTCT TGGCATTTTC ATTCGATCAT TATATACCAT
HapZ                AAT------C TAATACATAG AAAAGAATAA TATATATGAA TAATATAATA AAGAGAAAAT GCCAATCTCT TGGCATTTTC ATTCGATCAT TATATACCAT
HapD                AAT------C TAATACATAG AAAAGAATAA TATATATGAA TAATATAATA AAGAGAAAAT GCCAATCTCT TGGCATTTTC ATTCGATCAT TATATACCAT
HapA                AAT------C TAATACATAG AAAAGAATAA TATATATGAA TAATATAATA AAGAGAAAAT GCCAATCTCT TGGCATTTTC ATTCGATCAT TATATACCAT
HapB                AAT------C TAATACATAG AAAAGAATAA TATATATGAA TAATATAATA AAGAGAAAAT GCCAATCTCT TGGCATTTTC ATTCGATCAT TATATACCAT
HapC                AAT------C TAATACATAG AAAAGAATAA TATATATGAA TAATATAATA AAGAGAAAAT GCCAATCTCT TGGCATTTTC ATTCGATCAT TATATACCAT
HapY                AAT------C TAATACATAG AAAAGAATAA TATATATGAA TAATATAATA AAGAGAAAAT GCCAATCTCT TGGCATTTTC ATTCGATCAT TATATACCAT
HapW                AAT------C TAATACATAG AAAAGAATAA TATATATGAA TAATATAATA AAGAGAAAAT GCCAATCTCT TGGCATTTTC ATTCGATCAT TATATACCAT
HapX                AAT------C TAATACATAG AAAAGAATAA TATATATGAA TAATATAATA AAGAGAAAAT GCCAATCTCT TGGCATTTTC ATTCGATCAT TATATACCAT
HapS                AAT------C TAATACATAG AAAAGAATAA TATATATGAA TAATATAATA AAGAGAAAAT GCCAATCTCT TGGCATTTTC ATTCGATCAT TATATACCAT
HaKonecNC_025235    AAT------C TAATACATAG AAAAGAATAA TATATATGAA TAATATAATA AAGAGAAAAT GCCAAGAGAT TGGCATTTTC ATTCGATCAT TATATACCAT
Monparadoxtrnt      AAT------C TAATACATAG AAAAGAATAA TATATATGAA TAATATAACA AAGAGAAAAT GCCAAGAGAT TGGCATTTTC ------TCTT TA-------T

                    ....|....| ....|....| ....|....| ....|....| ....|....| ....|....| ....|....| ....|....| ....|....| ....|....|
                         505        515        525        535        545        555        565        575        585        595

HapM                TATATCCATT TTTTGAGATA TTTTGTTTTT TTATTATTTG TTAATAATTT AAGGATTAAT ATTTCACTAA GGAGAAGATA GAATCATAGC AAATGAAATT
HapM1               TATATCCATT TTTTGAGATA TTTTGTTTTT TTATTATTTG TTAATAATTT AAGGATTAAT ATTTCACTAA GGAGAAGATA GAATCATAGC AAATGAAATT
FU12.1              TATATCCATT TTTTGAGATA TTTTGTTTTT TTATTATTTG TTAATAATTT AAGGATTAAT ATTTCACTAA GGAGAAGATA GAATCATAGC AAATGAAATT
FU10.1              TATATCCATT TTTTGAGATA TTTTGTTTTT TTATTATTTG TTAATAATTT AAGGATTAAT ATTTCACTAA GGAGAAGATA GAATCATAGC AAATGAAATT
FU1.1               TATATCCATT TTTTGAGATA TTTTGTTTTT TTATTATTTG TTAATAATTT AAGGATTAAT ATTTCACTAA GGAGAAGATA GAATCATAGC AAATGAAATT
FU2.1               TATATCCATT TTTTGAGATA TTTTGTTTTT TTATTATTTG TTAATAATTT AAGGATTAAT ATTTCACTAA GGAGAAGATA GAATCATAGC AAATGAAATT
FU11.1              TATATCCATT TTTTGAGATA TTTTGTTTTT TTATTATTTG TTAATAATTT AAGGATTAAT ATTTCACTAA GGAGAAGATA GAATCATAGC AAATGAAATT
CO4.1               TATATCCATT TTTTGAGATA TTTTGTTTTT TTATTATTTG TTAATAATTT AAGGATTAAT ATTTCACTAA GGAGAAGATA GAATCATAGC AAATGAAATT
CO11.1              TATATCCATT TTTTGAGATA TTTTGTTTTT TTATTATTTG TTAATAATTT AAGGATTAAT ATTTCACTAA GGAGAAGATA GAATCATAGC AAATGAAATT
TR1.1               TATATCCATT TTTTGAGATA TTTTGTTTTT TTATTATTTG TTAATAATTT AAGGATTAAT ATTTCACTAA GGAGAAGATA GAATCATAGC AAATGAAATT
TR3.1               TATATCCATT TTTTGAGATA TTTTGTTTTT TTATTATTTG TTAATAATTT AAGGATTAAT ATTTCACTAA GGAGAAGATA GAATCATAGC AAATGAAATT
TR10.1              TATATCCATT TTTTGAGATA TTTTGTTTTT TTATTATTTG TTAATAATTT AAGGATTAAT ATTTCACTAA GGAGAAGATA GAATCATAGC AAATGAAATT
TR11.1              TATATCCATT TTTTGAGATA TTTTGTTTTT TTATTATTTG TTAATAATTT AAGGATTAAT ATTTCACTAA GGAGAAGATA GAATCATAGC AAATGAAATT
Vi16.1              TATATCCATT TTTTGAGATA TTTTGTTTTT TTATTATTTG TTAATAATTT AAGGATTAAT ATTTCACTAA GGAGAAGATA GAATCATAGC AAATGAA-TT
VI1.1               TATATCCATT TTTTGAGATA TTTTGTTTTT TTATTATTTG TTAATAATTT AAGGATTAAT ATTTCACTAA GGAGAAGATA GAATCATAGC AAATGAA-TT
Vi3.1               TATATCCATT TTTTGAGATA TTTTGTTTTT TTATTATTTG TTAATAATTT AAGGATTAAT ATTTCACTAA GGAGAAGATA GAATCATAGC AAATGAA-TT
CH1.1               TATATCCATT TTTTGAGATA TTTTGTTTTT TTATTATTTG TTAATAATTT AAGGATTAAT ATTTCACTAA GGAGAAGATA GAATCATAGC AAATGAAATT
CH4.1               TATATCCATT TTTTGAGATA TTTTGTTTTT TTATTATTTG TTAATAATTT AAGGATTAAT ATTTCACTAA GGAGAAGATA GAATCATAGC AAATGAAATT
CH6.1               TATATCCATT TTTTGAGATA TTTTGTTTTT TTATTATTTG TTAATAATTT AAGGATTAAT ATTTCACTAA GGAGAAGATA GAATCATAGC AAATGAAATT
CH2.2               TATATCCATT TTTTGAGATA TTTTGTTTTT TTATTATTTG TTAATAATTT AAGGATTAAT ATTTCACTAA GGAGAAGATA GAATCATAGC AAATGAAATT
CH2.1               TATATCCATT TTTTGAGATA TTTTGTTTTT TTATTATTTG TTAATAATTT AAGGATTAAT ATTTCACTAA GGAGAAGATA GAATCATAGC AAATGAAATT
HapAD               TATATCCATT TTTTGAGATA TTTTGTTTTT TTATTATTTG TTAATAATTT AAGGATTAAT ATTTCACTAA GGAGAAGATA GAATCATAGC AAATGAAATT
HapO                TATATCCATT TTTTGAGATA TTTTGTTTTT TTATTATTTG TTAATAATTT AAGGATTAAT ATTTCACTAA GGAGAAGATA GAATCATAGC AAATGAAATT
HapN                TATATCCATT TTTTGAGATA TTTTGTTTTT TTATTATTTG TTAATAATTT AAGGATTAAT ATTTCACTAA GGAGAAGATA GAATCATAGC AAATGAAATT
HapK                TATATCCATT TTTTGAGATA TTTTGTTTTT TTATTATTTG TTAATAATTT AAGGATTAAT ATTTCACTAA GGAGAAGATA GAATCATAGC AAATGAAATT
HapJ                TATATCCATT TTTTGAGATA TTTTGTTTTT TTATTATTTG TTAATAATTT AAGGATTAAT ATTTCACTAA GGAGAAGATA GAATCATAGC AAATGAAATT
HapL                TATATCCATT TTTTGAGATA TTTTGTTTTT TTATTATTTG TTAATAATTT AAGGATTAAT ATTTCACTAA GGAGAAGATA GAATCATAGC AAATGAAATT
HapQ                TATATCCATT TTTTGAGATA TTTTGTTTTT TTATTATTTG TTAATAATTT AAGGATTAAT ATTTCACTAA GGAGAAGATA GAATCATAGC AAATGAAATT
HapV                TATATCCATT TTTTGAGATA TTTTGTTTTT TTATTATTTG TTAATAATTT AAGGATTAAT ATTTCACTAA GGAGAAGATA GAATCATAGC AAATGAAATT
HapR                TATATCCATT TTTTGAGATA TTTTGTTTTT TTATTATTTG TTAATAATTT AAGGATTAAT ATTTCACTAA GGAGAAGATA GAATCATAGC AAATGAAATT
HapP                TATATCCATT TTTTGAGATA TTTTGTTTTT TTATTATTTG TTAATAATTT AAGGATTAAT ATTTCACTAA GGAGAAGATA GAATCATAGC AAATGAAATT
HapU                TATATCCATT TTTTGAGATA TTTTGTTTTT TTATTATTTG TTAATAATTT AAGGATTAAT ATTTCACTAA GGAGAAGATA GAATCATAGC AAATGAAATT
HapT                TATATCCATT TTTTGAGATA TTTTGTTTTT TTATTATTTG TTAATAATTT AAGGATTAAT ATTTCACTAA GGAGAAGATA GAATCATAGC AAATGAAATT
HapAA               TATATCCATT TTTTGAGATA TTTTGTTTTT TTATTATTTG TTAATAATTT AAGGATTAAT ATTTCACTAA GGAGAAGATA GAATCATAGC AAATGAAATT
HapH30              TATATCCATT TTTTGAGATA TTTTGTTTTT TTATTATTTG TTAATAATTT AAGGATTAAT ATTTCACTAA GGAGAAGATA GAATCATAGC AAATGAAATT
HapI                TATATCCATT TTTTGAGATA TTTTGTTTTT TTATTATTTG TTAATAATTT AAGGATTAAT ATTTCACTAA GGAGAAGATA GAATCATAGC AAATGAAATT
HapE                TATATCCATT TTTTGAGATA TTTTGTTTTT TTATTATTTG TTAATAATTT AAGGATTAAT ATTTCACTAA GGAGAAGATA GAATCATAGC AAATGAAATT
HapAC               TATATCCATT TTTTGAGATA TTTTGTTTTT TTATTATTTG TTAATAATTT AAGGATTAAT ATTTCACTAA GGAGAAGATA GAATCATAGC AAATGAAATT
HapF                TATATCCATT TTTTGAGATA TTTTGTTTTT TTATTATTTG TTAATAATTT AAGGATTAAT ATTTCACTAA GGAGAAGATA GAATCATAGC AAATGAAATT
HapH                TATATCCATT TTTTGAGATA TTTTGTTTTT TTATTATTTG TTAATAATTT AAGGATTAAT ATTTCACTAA GGAGAAGATA GAATCATAGC AAATGAAATT
HapG                TATATCCATT TTTTGAGATA TTTTGTTTTT TTATTATTTG TTAATAATTT AAGGATTAAT ATTTCACTAA GGAGAAGATA GAATCATAGC AAATGAAATT
HapAB               TATATCCATT TTTTGAGATA TTTTGTTTTT TTATTATTTG TTAATAATTT AAGGATTAAT ATTTCACTAA GGAGAAGATA GAATCATAGC AAATGAAATT
HapZ                TATATCCATT TTTTGAGATA TTTTGTTTTT TTATTATTTG TTAATAATTT AAGGATTAAT ATTTCACTAA GGAGAAGATA GAATCATAGC AAATGAAATT
HapD                TATATCCATT TTTTGAGATA TTTTGTTTTT TTATTATTTG TTAATAATTT AAGGATTAAT ATTTCACTAA GGAGAAGATA GAATCATAGC AAATGAAATT
HapA                TATATCCATT TTTTGAGATA TTTTGTTTTT TTATTATTTG TTAATAATTT AAGGATTAAT ATTTCACTAA GGAGAAGATA GAATCATAGC AAATGAAATT
HapB                TATATCCATT TTTTGAGATA TTTTGTTTTT TTATTATTTG TTAATAATTT AAGGATTAAT ATTTCACTAA GGAGAAGATA GAATCATAGC AAATGAAATT
HapC                TATATCCATT TTTTGAGATA TTTTGTTTTT TTATTATTTG TTAATAATTT AAGGATTAAT ATTTCACTAA GGAGAAGATA GAATCATAGC AAATGAAATT
HapY                TATATCCATT TTTTGAGATA TTTTGTTTTT TTATTATTTG TTAATAATTT AAGGATTAAT ATTTCACTAA GGAGAAGATA GAATCATAGC AAATGAAATT
HapW                TATATCCATT TTTTGAGATA TTTTGTTTTT TTATTATTTG TTAATAATTT AAGGATTAAT ATTTCACTAA GGAGAAGATA GAATCATAGC AAATGAAATT
HapX                TATATCCATT TTTTGAGATA TTTTGTTTTT TTATTATTTG TTAATAATTT AAGGATTAAT ATTTCACTAA GGAGAAGATA GAATCATAGC AAATGAAATT
HapS                TATATCCATT TTTTGAGATA TTTTGTTTTT TTATTATTTG TTAATAATTT AAGGATTAAT ATTTCACTAA GGAGAAGATA GAATCATAGC AAATGAAATT
HaKonecNC_025235    TATATACATT TTTTGAGATA TTTTGTTTTT TTATTATTTG TTAATAATTT AAGGATTAAT ATTTCACTAA GGAGAAGATA GAATCATAGC AAATGAAATT
Monparadoxtrnt      TATATACATT TTTTGAGATA -----TTTTT TTGTTATTTG TTAATAATTT AAGGATTAAT ATTTCACTAA GGAGAAGATA GAATCATAGC AAATGAAATT
```

```
                     ....|....| ....|....| ....|....| ....|....| ....|....| ....|....| ....|....| ....|....| ....|....| ....|....|
                           605        615        625        635        645        655        665        675        685        695

HapM                 TCTAATTCTG ATTAG--AAA AAAAAAGAAT GAATATCAAG CGTTATAGTA TGATTTTGAA TACTCTAAAA AAGGAAGGA- GGGGGGTGGG GGAGAGAAAA
HapM1                TCTAATTCTG ATTAG---AA AAAAAAGAAT GAATATCAAG CGTTATAGTA TGATTTTGAA TACTCTAAAA AAGGAAGGA- GGGGGGTGGG GGAGAGAAAA
FU12.1               TCTAATTCTG ATTAG--AAA AAAAAAGAAT GAATATCAAG CGTTATAGTA TGATTTTGAA TACTCTAAAA AAGGAAGGA- GGGGGGTGGG GGAGAGAAAA
FU10.1               TCTAATTCTG ATTAG--AAA AAAAAAGAAT GAATATCAAG CGTTATAGTA TGATTTTGAA TACTCTAAAA AAGGAAGGA- GGGGGGTGGG GGAGAGAAAA
FU1.1                TCTAATTCTG ATTAG--AAA AAAAAAGAAT GAATATCAAG CGTTATAGTA TGATTTTGAA TACTCTAAAA AAGGAAGGA- GGGGGGTGGG GGAGAGAAAA
FU2.1                TCTAATTCTG ATTAG--AAA AAAAAAGAAT GAATATCAAG CGTTATAGTA TGATTTTGAA TACTCTAAAA AAGGAAGGA- GGGGGGTGGG GGAGAGAAAA
FU11.1               TCTAATTCTG ATTAG--AAA AAAAAAGAAT GAATATCAAG CGTTATAGTA TGATTTTGAA TACTCTAAAA AAGGAAGGA- GGGGGGTGGG GGAGAGAAAA
CO4.1                TCTAATTCTG ATTAG--AAA AAAAAAGAAT GAATATCAAG CGTTATAGTA TGATTTTGAA TACTCTAAAA AAGGAAGGA- GGGGGGTGGG GGAGAGAAAA
CO11.1               TCTAATTCTG ATTAG--AAA AAAAAAGAAT GAATATCAAG CGTTATAGTA TGATTTTGAA TACTCTAAAA AAGGAAGGA- GGGGGGTGGG GGAGAGAAAA
TR1.1                TCTAATTCTG ATTAG--AAA AAAAAAGAAT GAATATCAAG CGTTATAGTA TGATTTTGAA TACTCTAAAA AAGGAAGGA- GGGGGGTGGG GGAGAGAAAA
TR3.1                TCTAATTCTG ATTAG--AAA AAAAAAGAAT GAATATCAAG CGTTATAGTA TGATTTTGAA TACTCTAAAA AAGGAAGGA- GGGGGGTGGG GGAGAGAAAA
TR10.1               TCTAATTCTG ATTAG--AAA AAAAAAGAAT GAATATCAAG CGTTATAGTA TGATTTTGAA TACTCTAAAA AAGGAAGGA- GGGGGGTGGG GGAGAGAAAA
TR11.1               TCTAATTCTG ATTAG--AAA AAAAAAGAAT GAATATCAAG CGTTATAGTA TGATTTTGAA TACTCTAAAA AAGGAAGGA- GGGGGGTGGG GGAGAGAAAA
Vi16.1               TCTAATTCTG ATTAG---AA AAAAAAGAAT GAATATCAAG CGTTATAGTA TGATTTTGAA TACTCTAAAA AAGGAAGGA- GGGGGGTGGG GGAGAGAAAA
VI1.1                TCTAATTCTG ATTAG---AA AAAAAAGAAT GAATATCAAG CGTTATAGTA TGATTTTGAA TACTCTAAAA AAGGAAGGA- GGGGGGTGGG GGAGAGAAAA
Vi3.1                TCTAATTCTG ATTAG---AA AAAAAAGAAT GAATATCAAG CGTTATAGTA TGATTTTGAA TACTCTAAAA AAGGAAGGA- GGGGGGTGGG GGAGAGAAAA
CH1.1                TCTAATTCTG ATTAG--AAA AAAAAAGAAT GAATATCAAG CGTTATAGTA TGATTTTGAA TACTCTAAAA AAGGAAGGA- GGGGGGTGGG GGAGAGAAAA
CH4.1                TCTAATTCTG ATTAG--AAA AAAAAAGAAT GAATATCAAG CGTTATAGTA TGATTTTGAA TACTCTAAAA AAGGAAGGA- GGGGGGTGGG GGAGAGAAAA
CH6.1                TCTAATTCTG ATTAG--AAA AAAAAAGAAT GAATATCAAG CGTTATAGTA TGATTTTGAA TACTCTAAAA AAGGAAGGA- GGGGGGTGGG GGAGAGAAAA
CH2.2                TCTAATTCTG ATTAG--AAA AAAAAAGAAT GAATATCAAG CGTTATAGTA TGATTTTGAA TACTCTAAAA AAGGAAGGA- GGGGGGTGGG GGAGAGAAAA
CH2.1                TCTAATTCTG ATTAG--AAA AAAAAAGAAT GAATATCAAG CGTTATAGTA TGATTTTGAA TACTCTAAAA AAGGAAGGA- GGGGGGTGGG GGAGAGAAAA
HapAD                TCTAATTCTG ATTAG--AAA AAAAAAGAAT GAATATCAAG CGTTATAGTA TGATTTTGAA TACTCTAAAA AAGGAAGGA- GGGGGGTGGG GGAGAGAAAA
HapO                 TCTAATTCTG ATTAG-AAAA AAAAAAGAAT GAATATCAAG CGTTATAGTA TGATTTTGAA TACTCTAAAA AAGGAAGGA- GGGGGGTGGG GGAGAGAAAA
HapN                 TCTAATTCTG ATTAG-AAAA AAAAAAGAAT GAATATCAAG CGTTATAGTA TGATTTTGAA TACTCTAAAA AAGGAAGGA- GGGGGGTGGG GGAGAGAAAA
HapK                 TCTAATTCTG ATTAG--AAA AAAAAAGAAT GAATATCAAG CGTTATAGTA TGATTTTGAA TACTCTAAAA AAGGAAGGA- GGGGGGTGGG GGAGAGAAAA
HapJ                 TCTAATTCTG ATTAGAAAAA AAAAAAGAAT GAATATCAAG CGTTATAGTA TGATTTTGAA TACTCTAAAA AAGGAAGGA- GGGGGGTGGG GGAGAGAAAA
HapL                 TCTAATTCTG ATTAGAAAAA AAAAAAGAAT GAATATCAAG CGTTATAGTA TGATTTTGAA TACTCTAAAA AAGGAAGGA- GGGGGGTGGG GGAGAGAAAA
HapQ                 TCTAATTCTG ATTAGAAAAA AAAAAAGAAT GAATATCAAG CGTTATAGTA TGATTTTGAA TACTCTAAAA AAGGAAGGA- GGGGGGTGGG GGAGAGAAAA
HapV                 TCTAATTCTG ATTAGAAAAA AAAAAAGAAT GAATATCAAG CGTTATAGTA TGATTTTGAA TACTCTAAAA AAGGAAGGA- GGGGGGTGGG GGAGAGAAAA
HapR                 TCTAATTCTG ATTAGAAAAA AAAAAAGAAT GAATATCAAG CGTTATAGTA TGATTTTGAA TACTCTAAAA AAGGAAGGA- GGGGGGTGGG GGAGAGAAAA
HapP                 TCTAATTCTG ATTAGAAAAA AAAAAAGAAT GAATATCAAG CGTTATAGTA TGATTTTGAA TACTCTAAAA AAGGAAGGA- GGGGGGTGGG GGAGAGAAAA
HapU                 TCTAATTCTG ATTAGAAAAA AAAAAAGAAT GAATATCAAG CGTTATAGTA TGATTTTGAA TACTCTAAAA AAGGAAGGA- GGGGGGTGGG GGAGAGAAAA
HapT                 TCTAATTCTG ATTAGAAAAA AAAAAAGAAT GAATATCAAG CGTTATAGTA TGATTTTGAA TACTCTAAAA AAGGAAGGA- GGGGGGTGGG GGAGAGAAAA
HapAA                TCTAATTCTG ATTAG-AAAA AAAAAAGAAT GAATATCAAG CGTTATAGTA TGATTTTGAA TACTCTAAAA AAGGAAGGAG GGGGGGTGGG GGAGAGAAAA
HapH30               TCTAATTCTG ATTAG-AAAA AAAAAAGAAT GAATATCAAG CGTTATAGTA TGATTTTGAA TACTCTAAAA AAGGAAGGA- GGGGGGTGGG GGAGAGAAAA
HapI                 TCTAATTCTG ATTAGAAAAA AAAAAAGAAT GAATATCAAG CGTTATAGTA TGATTTTGAA TACTCTAAAA AAGGAAGGA- GGGGGGTGGG GGAGAGAAAA
HapE                 TCTAATTCTG ATTAG-AAAA AAAAAAGAAT GAATATCAAG CGTTATAGTA TGATTTTGAA TACTCTAAAA AAGGAAGGAG GGGGGGTGGG GGAGAGAAAA
HapAC                TCTAATTCTG ATTAG-AAAA AAAAAAGAAT GAATATCAAG CGTTATAGTA TGATTTTGAA TACTCTAAAA AAGGAAGGAG GGGGGGTGGG GGAGAGAAAA
HapF                 TCTAATTCTG ATTAG-AAAA AAAAAAGAAT GAATATCAAG CGTTATAGTA TGATTTTGAA TACTCTAAAA AAGGAAGGAG GGGGGGTGGG GGAGAGAAAA
HapH                 TCTAATTCTG ATTAG-AAAA AAAAAAGAAT GAATATCAAG CGTTATAGTA TGATTTTGAA TACTCTAAAA AAGGAAGGAG GGGGGGTGGG GGAGAGAAAA
HapG                 TCTAATTCTG ATTAG-AAAA AAAAAAGAAT GAATATCAAG CGTTATAGTA TGATTTTGAA TACTCTAAAA AAGGAAGGAG GGGGGGTGGG GGAGAGAAAA
HapAB                TCTAATTCTG ATTAG-AAAA AAAAAAGAAT GAATATCAAG CGTTATAGTA TGATTTTGAA TACTCTAAAA AAGGAAGGAG GGGGGGTGGG GGAGAGAAAA
HapZ                 TCTAATTCTG ATTAG-AAAA AAAAAAGAAT GAATATCAAG CGTTATAGTA TGATTTTGAA TACTCTAAAA AAGGAAGGAG GGGGGGTGGG GGAGAGAAAA
HapD                 TCTAATTCTG ATTAG-AAAA AAAAAAGAAT GAATATCAAG CGTTATAGTA TGATTTTGAA TACTCTAAAA AAGGAAGGAG GGGGGGTGGG GGAGAGAAAA
HapA                 TCTAATTCTG ATTAG-AAAA AAAAAAGAAT GAATATCAAG CGTTATAGTA TGATTTTGAA TACTCTAAAA AAGGAAGGAG GGGGGGTGGG GGAGAGAAAA
HapB                 TCTAATTCTG ATTAG-AAAA AAAAAAGAAT GAATATCAAG CGTTATAGTA TGATTTTGAA TACTCTAAAA AAGGAAGGAG GGGGGGTGGG GGAGAGAAAA
HapC                 TCTAATTCTG ATTAG-AAAA AAAAAAGAAT GAATATCAAG CGTTATAGTA TGATTTTGAA TACTCTAAAA AAGGAAGGAG GGGGGGTGGG GGAGAGAAAA
HapY                 TCTAATTCTG ATTAG-AAAA AAAAAAGAAT GAATATCAAG CGTTATAGTA TGATTTTGAA TACTCTAAAA AAGGAAGGA- GGGGGGTGGG GGAGAGAAAA
HapW                 TCTAATTCTG ATTAG-AAAA AAAAAAGAAT GAATATCAAG CGTTATAGTA TGATTTTGAA TACTCTAAAA AAGGAAGGA- GGGGGGTGGG GGAGAGAAAA
HapX                 TCTAATTCTG ATTAG-AAAA AAAAAAGAAT GAATATCAAG CGTTATAGTA TGATTTTGAA TACTCTAAAA AAGGAAGGA- GGGGGGTGGG GGAGAGAAAA
HapS                 TCTAATTCTG ATTAG-AAAA AAAAAAGAAT GAATATCAAG CGTTATAGTA TGATTTTGAA TACTCTAAAA AAGGAAGGAG GGGGGGTGGG GGAGAGAAAA
HaKonecNC_025235     TCTAATTCTG ATTAG-AAAA AAAA--GAAT TAATATCAAG CGTTATAGTA TGATTTTGAA TACTCTAAAA AAGGAAGGG- GGGAGGTGGG GGAGAGAAAA
Monparadoxtrnt       TCTAATTCTG ATTAG-AAAA AAAAA-GAAT GAATATCAAG CGTTATAGTA AGATTTTGAA TACTCTAAAA AAATAAGGGG GATAAGCGGG GGAGAGAAAA

                     ....|....| ....|....| ....|....| ....|....| ....|....| ....|....| ....|....| ....|....| ....|....| ....|....|
                           705        715        725        735        745        755        765        775        785        795

HapM                 ACTTTTGGAT ATATTGATTC C-GATTGAAT TACAAATATA TCAACGATAG AATCAATTCA ATTCTGAATT GCAATAAGCG GGGTCTCTCA AAGAGAGACG
HapM1                ACTTTTGGAT ATATTGATTC C-GATTGAAT TACAAATATA TCAACGATAG AATCAATTCA ATTCTGAATT GCAATAAGCG GGGTCTCTCA AAGAGAGACG
FU12.1               ACTTTTGGAT ATATTGATTC C-GATTGAAT TACAAATATA TCAACGATAG AATCAATTCA ATTCTGAATT GCAATAAGCG GGGTCTCTCA AAGAGAGACG
FU10.1               ACTTTTGGAT ATATTGATTC C-GATTGAAT TACAAATATA TCAACGATAG AATCAATTCA ATTCTGAATT GCAATAAGCG GGGTCTCTCA AAGAGAGACG
FU1.1                ACTTTTGGAT ATATTGATTC C-GATTGAAT TACAAATATA TCAACGATAG AATCAATTCA ATTCTGAATT GCAATAAGCG GGGTCTCTCA AAGAGAGACG
FU2.1                ACTTTTGGAT ATATTGATTC C-GATTGAAT TACAAATATA TCAACGATAG AATCAATTCA ATTCTGAATT GCAATAAGCG GGGTCTCTCA AAGAGAGACG
FU11.1               ACTTTTGGAT ATATTGATTC C-GATTGAAT TACAAATATA TCAACGATAG AATCAATTCA ATTCTGAATT GCAATAAGCG GGGTCTCTCA AAGAGAGACG
CO4.1                ACTTTTGGAT ATATTGATTC C-GATTGAAT TACAAATATA TCAACGATAG AATCAATTCA ATTCTGAATT GCAATAAGCG GGGTCTCTCA AAGAGAGACG
CO11.1               ACTTTTGGAT ATATTGATTC C-GATTGAAT TACAAATATA TCAACGATAG AATCAATTCA ATTCTGAATT GCAATAAGCG GGGTCTCTCA AAGAGAGACG
TR1.1                ACTTTTGGAT ATATTGATTC C-GATTGAAT TACAAATATA TCAACGATAG AATCAATTCA ATTCTGAATT GCAATAAGCG GGGTCTCTCA AAGAGAGACG
TR3.1                ACTTTTGGAT ATATTGATTC C-GATTGAAT TACAAATATA TCAACGATAG AATCAATTCA ATTCTGAATT GCAATAAGCG GGGTCTCTCA AAGAGAGACG
TR10.1               ACTTTTGGAT ATATTGATTC C-GATTGAAT TACAAATATA TCAACGATAG AATCAATTCA ATTCTGAATT GCAATAAGCG GGGTCTCTCA AAGAGAGACG
TR11.1               ACTTTTGGAT ATATTGATTC C-GATTGAAT TACAAATATA TCAACGATAG AATCAATTCA ATTCTGAATT GCAATAAGCG GGGTCTCTCA AAGAGAGACG
Vi16.1               ACTTTTGGAT ATATTGATTC C-GATTGAAT TACAAATATA TCAACGATAG AATCAATTCA ATTCTGAATT GCAATAAGCG GGGTCTCTCA AAGAGAGACG
VI1.1                ACTTTTGGAT ATATTGATTC C-GATTGAAT TACAAATATA TCAACGATAG AATCAATTCA ATTCTGAATT GCAATAAGCG GGGTCTCTCA AAGAGAGACG
Vi3.1                ACTTTTGGAT ATATTGATTC C-GATTGAAT TACAAATATA TCAACGATAG AATCAATTCA ATTCTGAATT GCAATAAGCG GGGTCTCTCA AAGAGAGACG
CH1.1                ACTTTTGGAT ATATTGATTC C-GATTGAAT TACAAATATA TCAACGATAG AATCAATTCA ATTCTGAATT GCAATAAGCG GGGTCTCTCA AAGAGAGACG
CH4.1                ACTTTTGGAT ATATTGATTC C-GATTGAAT TACAAATATA TCAACGATAG AATCAATTCA ATTCTGAATT GCAATAAGCG GGGTCTCTCA AAGAGAGACG
CH6.1                ACTTTTGGAT ATATTGATTC C-GATTGAAT TACAAATATA TCAACGATAG AATCAATTCA ATTCTGAATT GCAATAAGCG GGGTCTCTCA AAGAGAGACG
CH2.2                ACTTTTGGAT ATATTGATTC C-GATTGAAT TACAAATATA TCAACGATAG AATCAATTCA ATTCTGAATT GCAATAAGCG GGGTCTCTCA AAGAGAGACG
CH2.1                ACTTTTGGAT ATATTGATTC C-GATTGAAT TACAAATATA TCAACGATAG AATCAATTCA ATTCTGAATT GCAATAAGCG GGGTCTCTCA AAGAGAGACG
HapAD                ACTTTTGGAT ATATTGATTC C-GATTGAAT TACAAATATA TCAACGATAG AATCAATTCA ATTCTGAATT GCAATAAGCG GGGTCTCTCA AAGAGAGACG
HapO                 ACTTTTGGAT ATATTGATTC C-GATTGAAT TACAAATATA TCAACGATAG AATCAATTCA ATTCTGAATT GCAATAAGCG GGGTCTCTCA AAGAGAGACG
HapN                 ACTTTTGGAT ATATTGATTC C-GATTGAAT TACAAATATA TCAACGATAG AATCAATTCA ATTCTGAATT GCAATAAGCG GGGTCTCTCA AAGAGAGACG
HapK                 ACTTTTGGAT ATATTGATTC C-GATTGAAT TACAAATATA TCAACGATAG AATCAATTCA ATTCTGAATT GCAATAAGCG GGGTCTCTCA AAGAGAGACG
HapJ                 ACTTTTGGAT ATATTGATTC C-GATTGAAT TACAAATATA TCAACGATAG AATCAATTCA ATTCTGAATT GCAATAAGCG GGGTCTCTCA AAGAGAGACG
HapL                 ACTTTTGGAT ATATTGATTC C-GATTGAAT TACAAATATA TCAACGATAG AATCAATTCA ATTCTGAATT GCAATAAGCG GGGTCTCTCA AAGAGAGACG
HapQ                 ACTTTTGGAT ATATTGATTC C-GATTGAAT TACAAATATA TCAACGATAG AATCAATTCA ATTCTGAATT GCAATAAGCG GGGTCTCTCA AAGAGAGACG
HapV                 ACTTTTGGAT ATATTGATTC C-GATTGAAT TACAAATATA TCAACGATAG AATCAATTCA ATTCTGAATT GCAATAAGCG GGGTCTCTCA AAGAGAGACG
HapR                 ACTTTTGGAT ATATTGATTC C-GATTGAAT TACAAATATA TCAACGATAG AATCAATTCA ATTCTGAATT GCAATAAGCG GGGTCTCTCA AAGAGAGACG
HapP                 ACTTTTGGAT ATATTGATTC C-GATTGAAT TACAAATATA TCAACGATAG AATCAATTCA ATTCTGAATT GCAATAAGCG GGGTCTCTCA AAGAGAGACG
HapU                 ACTTTTGGAT ATATTGATTC C-GATTGAAT TACAAATATA TCAACGATAG AATCAATTCA ATTCTGAATT GCAATAAGCG GGGTCTCTCA AAGAGAGACG
HapT                 ACTTTTGGAT ATATTGATTC C-GATTGAAT TACAAATATA TCAACGATAG AATCAATTCA ATTCTGAATT GCAATAAGCG GGGTCTCTCA AAGAGAGACG
HapAA                ACTTTTGGAT ATATTGATTC C-GATTGAAT TACAAATATA TCAACGATAG AATCAATTCA ATTCTAAATT GCAATAAGCG GGGTCTCTCA AAGAGAGACG
HapH30               ACTTTTGGAT ATATTGATTC C-GATTGAAT TACAAATATA TCAACGATAG AATCAATTCA ATTCTGAATT GCAATAAGCG GGGTCTCTCA AAGAGAGACG
HapI                 ACTTTTGGAT ATATTGATTC C-GATTGAAT TACAAATATA TCAACGATAG AATCAATTCA ATTCTGAATT GCAATAAGCG GGGTCTCTCA AAGAGAGACG
HapE                 ACTTTTGGAT ATATTGATTC C-GATTGAAT TACAAATATA TCAACGATAG AATCAATTCA ATTCTAAATT GCAATAAGCG GGGTCTCTCA AAGAGAGACG
HapAC                ACTTTTGGAT ATATTGATTC C-GATTGAAT TACAAATATA TCAACGATAG AATCAATTCA ATTCTAAATT GCAATAAGCG GGGTCTCTCA AAGAGAGACG
HapF                 ACTTTTGGAT ATATTGATTC C-GATTGAAT TACAAATATA TCAACGATAG AATCAATTCA ATTCTAAATT GCAATAAGCG GGGTCTCTCA AAGAGAGACG
HapH                 ACTTTTGGAT ATATTGATTC C-GATTGAAT TACAAATATA TCAACGATAG AATCAATTCA ATTCTGAATT GCAATAAGCG GGGTCTCTCA AAGAGAGACG
HapG                 ACTTTTGGAT ATATTGATTC C-GATTGAAT TACAAATATA TCAACGATAG AATCAATTCA ATTCTGAATT GCAATAAGCG GGGTCTCTCA AAGAGAGACG
HapAB                ACTTTTGGAT ATATTGATTC C-GATTGAAT TACAAATATA TCAACGATAG AATCAATTCA ATTCTAAATT GCAATAAGCG GGGTCTCTCA AAGAGAGACG
HapZ                 ACTTTTGGAT ATATTGATTC C-GATTGAAT TACAAATATA TCAACGATAG AATCAATTCA ATTCTAAATT GCAATAAGCG GGGTCTCTCA AAGAGAGACG
HapD                 ACTTTTGGAT ATATTGATTC C-GATTGAAT TACAAATATA TCAACGATAG AATCAATTCA ATTCTGAATT GCAATAAGCG GGGTCTCTCA AAGAGAGACG
HapA                 ACTTTTGGAT ATATTGATTC C-GATTGAAT TACAAATATA TCAACGATAG AATCAATTCA ATTCTGAATT GCAATAAGCG GGGTCTCTCA AAGAGAGACG
HapB                 ACTTTTGGAT ATATTGATTC C-GATTGAAT TACAAATATA TCAACGATAG AATCAATTCA ATTCTGAATT GCAATAAGCG GGGTCTCTCA AAGAGAGACG
HapC                 ACTTTTGGAT ATATTGATTC C-GATTGAAT TACAAATATA TCAACGATAG AATCAATTCA ATTCTGAATT GCAATAAGCG GGGTCTCTCA AAGAGAGACG
HapY                 ACTTTTGGAT ATATTGATTC C-GATTGAAT TACAAATATA TCAACGATAG AATCAATTCA ATTCTGAATT GCAATAAGCG GGGTCTCTCA AAGAGAGACG
HapW                 ACTTTTGGAT ATATTGATTC C-GATTGAAT TACAAATATA TCAACGATAG AATCAATTCA ATTCTGAATT GCAATAAGCG GGGTCTCTCA AAGAGAGACG
HapX                 ACTTTTGGAT ATATTGATTC C-GATTGAAT TACAAATATA TCAACGATAG AATCAATTCA ATTCTGAATT GCAATAAGCG GGGTCTCTCA AAGAGAGACG
HapS                 ACTTTTGGAT ATATTGATTC C-GATTGAAT TACAAATATA TCAACGATAG AATCAATTCA ATTCTAAATT GCAATAAGCG GGGTCTCTCA AAGAGAGACG
HaKonecNC_025235     ACTTTTGGAT ATATTGATTC CCGAT-GAAT TGCAAATATA TCAACGATAG AATCAATTCA ATTCTGAATT GCAATAAGCG GGGTCTCTCA AAGAGAGACG
Monparadoxtrnt       ATTTTTGGAT ATATTGATTC C-GATTGAAT TGCAAATCGA TCAACGATAG AATCAATTCA ATTCTGAATT GCAATAAGCG AGGTCTCTCA AATAGAGACG

                     ....|....| ....|....| ....|....| ....|....| ....|....| ....|....| ....|....| ....|....| ....|....| ....|....|
                           805        815        825        835        845        855        865        875        885        895

HapM                 AGCTGCTAGA CTACGTCGAA TAATGAATTC AATGATTCAA AAAAAAAACT AAGA-GATGG GTGAAAT--- -----TACAC AAGGAATCCT CCGACAAGCT
HapM1                AGCTGCTAGA CTACGTCGAA TAATGAATTC AATGATTCAA AAAAAAAACT AAGA-GATGG GTGAAAT--- -----TACAC AAGGAATCCT CCGACAAGCT
FU12.1               AGCTGCTAGA CTACGTCGAA TAATGAATTC AATGATTCAA AAAAAAAACT AAGA-GATGG GTGAAAT--- -----TACAC AAGGAATCCT CCGACAAGCT
FU10.1               AGCTGCTAGA CTACGTCGAA TAATGAATTC AATGATTCAA AAAAAAAACT AAGA-GATGG GTGAAAT--- -----TACAC AAGGAATCCT CCGACAAGCT
FU1.1                AGCTGCTAGA CTACGTCGAA TAATGAATTC AATGATTCAA AAAAAAAACT AAGA-GATGG GTGAAAT--- -----TACAC AAGGAATCCT CCGACAAGCT
FU2.1                AGCTGCTAGA CTACGTCGAA TAATGAATTC AATGATTCAA AAAAAAAACT AAGA-GATGG GTGAAAT--- -----TACAC AAGGAATCCT CCGACAAGCT
FU11.1               AGCTGCTAGA CTACGTCGAA TAATGAATTC AATGATTCAA AAAAAAAACT AAGA-GATGG GTGAAAT--- -----TACAC AAGGAATCCT CCGACAAGCT
CO4.1                AGCTGCTAGA CTACGTCGAA TAATGAATTC AATGATTCAA AAAAAAAACT AAGAAGATGG GTGAAAT--- -----TACAC AAGGAATCCT CCGACAAGCT
CO11.1               AGCTGCTAGA CTACGTCGAA TAATGAATTC AATGATTCAA AAAAAAAACT AAGAAGATGG GTGAAAT--- -----TACAC AAGGAATCCT CCGACAAGCT
TR1.1                AGCTGCTAGA CTACGTCGAA TAATGAATTC AATGATTCAA AAAAAAAACT AAGA-GATGG GTGAAAT--- -----TACAC AAGGAATCCT CCGACAAGCT
TR3.1                AGCTGCTAGA CTACGTCGAA TAATGAATTC AATGATTCAA AAAAAAAACT AAGA-GATGG GTGAAAT--- -----TACAC AAGGAATCCT CCGACAAGCT
TR10.1               AGCTGCTAGA CTACGTCGAA TAATGAATTC AATGATTCAA AAAAAAAACT AAGA-GATGG GTGAAAT--- -----TACAC AAGGAATCCT CCGACAAGCT
TR11.1               AGCTGCTAGA CTACGTCGAA TAATGAATTC AATGATTCAA AAAAAAAACT AAGA-GATGG GTGAAAT--- -----TACAC AAGGAATCCT CCGACAAGCT
Vi16.1               AGCTGCTAGA CTACGTCGAA TAATGAATTC AATGATTCAA AAAAAAAACT AAGA-GATGG GTGAAA---- -----TACAC AAGGAATCCT CCGACAAGCT
VI1.1                AGCTGCTAGA CTACGTCGAA TAATGAATTC AATGATTCAA AAAAAAAACT AAGA-GATGG GTGAAA---- -----TACAC AAGGAATCCT CCGACAAGCT
Vi3.1                AGCTGCTAGA CTACGTCGAA TAATGAATTC AATGATTCAA AAAAAAAACT AAGA-GATGG GTGAAA---- -----TACAC AAGGAATCCT CCGACAAGCT
CH1.1                AGCTGCTAGA CTACGTCGAA TAATGAATTC AATGATTCAA AAAAAAAACT AAGA-GATGG GTGAAAT--- -----TACAC AAGGAATCCT CCGACAAGCT
CH4.1                AGCTGCTAGA CTACGTCGAA TAATGAATTC AATGATTCAA AAAAAAAACT AAGA-GATGG GTGAAAT--- -----TACAC AAGGAATCCT CCGACAAGCT
CH6.1                AGCTGCTAGA CTACGTCGAA TAATGAATTC AATGATTCAA AAAAAAAACT AAGA-GATGG GTGAAAT--- -----TACAC AAGGAATCCT CCGACAAGCT
CH2.2                AGCTGCTAGA CTACGTCGAA TAATGAATTC AATGATTCAA AAAAAAAACT AAGA-GATGG GTGAAAT--- -----TACAC AAGGAATCCT CCGACAAGCT
CH2.1                AGCTGCTAGA CTACGTCGAA TAATGAATTC AATGATTCAA AAAAAAAACT AAGA-GATGG GTGAAAT--- -----TACAC AAGGAATCCT CCGACAAGCT
HapAD                AGCTGCTAGA CTACGTCGAA TAATGAATTC AATGATTCAA AAAAAAAACT AAGA-GATGG GTGAAAT--- -----TACAC AAGGAATCCT CCGACAAGCT
HapO                 AGCTGCTAGA CTACGTCGAA TAATGAATTC AATGATTCAA AAAAAAAACT AAGA-GATGG GTGAAAT--- -----TACAC AAGGAATCCT CCGACAAGCT
HapN                 AGCTGCTAGA CTACGTCGAA TAATGAATTC AATGATTCAA AAAAAAAACT AAGA-GATGG GTGAAAT--- -----TACAC AAGGAATCCT CCGACAAGCT
HapK                 AGCTGCTAGA CTACGTCGAA TAATGAATTC AATGATTCAA AAAAAAAACT AAGA-GATGG GTGAAAT--- -----TACAC AAGGAATCCT CCGACAAGCT
HapJ                 AGCTGCTAGA CTACGTCGAA TAATGAATTC AATGATTCAA AAAAAAAACT AAGA-GATGG GTGAAAT--- -----TACAC AAGGAATCCT CCGACAAGCT
HapL                 AGCTGCTAGA CTACGTCGAA TAATGAATTC AATGATTCAA AAAAAAAACT AAGA-GATGG GTGAAAT--- -----TACAC AAGGAATCCT CCGACAAGCT
HapQ                 AGCTGCTAGA CTACGTCGAA TAATGAATTC AATGATTCAA AAAAAAAACT AAGA-GATGG GTGAAAT--- -----TACAC AAGGAATCCT CCGACAAGCT
HapV                 AGCTGCTAGA CTACGTCGAA TAATGAATTC AATGATTCAA AAAAAAAACT AAGA-GATGG GTGAAAT--- -----TACAC AAGGAATCCT CCGACAAGCT
HapR                 AGCTGCTAGA CTACGTCGAA TAATGAATTC AATGATTCAA AAAAAAAACT AAGA-GATGG GTGAAAT--- -----TACAC AAGGAATCCT CCGACAAGCT
HapP                 AGCTGCTAGA CTACGTCGAA TAATGAATTC AATGATTCAA AAAAAAAACT AAGA-GATGG GTGAAAT--- -----TACAC AAGGAATCCT CCGACAAGCT
HapU                 AGCTGCTAGA CTACGTCGAA TAATGAATTC AATGATTCAA AAAAAAAACT AAGA-GATGG GTGAAAT--- -----TACAC AAGGAATCCT CCGACAAGCT
HapT                 AGCTGCTAGA CTACGTCGAA TAATGAATTC AATGATTCAA AAAAAAAACT AAGA-GATGG GTGAAAT--- -----TACAC AAGGAATCCT CCGACAAGCT
HapAA                AGCTGCTAGA CTACGTCGAA TAATGAATTC AATGATTCAA AAAAAAAACT AAGA-GATGG GTGAAATTAC ACAAGTACAC AAGGAATCCT ----------
HapH30               AGCTGCTAGA CTACGTCGAA TAATGAATTC AATGATTCAA AAAAAAAACT AAGA-GATGG GTGAAAT--- -----TACAC AAGGAATCCT CCGACAAGCT
HapI                 AGCTGCTAGA CTACGTCGAA TAATGAATTC AATGATTCAA AAAAAAAACT AAGA-GATGG GTGAAAT--- -----TACAC AAGGAATCCT CCGACAAGCT
HapE                 AGCTGCTAGA CTACGTCGAA TAATGAATTC AATGATTCAA AAAAAAAACT AAGA-GATGG GTGAAATTAC ACAAGTACAC AAGGAATCCT CCGACAAGCT
HapAC                AGCTGCTAGA CTACGTCGAA TAATGAATTC AATGATTCAA AAAAAAAACT AAGA-GATGG GTGAAATTAC ACAAGTACAC AAGGAATCCT CCGACAAGCT
HapF                 AGCTGCTAGA CTACGTCGAA TAATGAATTC AATGATTCAA AAAAAAAACT AAGA-GATGG GTGAAATTAC ACAAGTACAC AAGGAATCCT CCGACAAGCT
HapH                 AGCTGCTAGA CTACGTCGAA TAATGAATTC AATGATTCAA AAAAAAAACT AAGA-GATGG GTGAAATTAC ACAAGTACAC AAGGAATCCT CCGACAAGCT
HapG                 AGCTGCTAGA CTACGTCGAA TAATGAATTC AATGATTCAA AAAAAAAACT AAGA-GATGG GTGAAATTAC ACAAGTACAC AAGGAATCCT CCGACAAGCT
HapAB                AGCTGCTAGA CTACGTCGAA TAATGAATTC AATGATTCAA AAAAAAAACT AAGA-GATGG GTGAAATTAC ACAAGTACAC AAGGAATCCT CCGACAAGCT
HapZ                 AGCTGCTAGA CTACGTCGAA TAATGAATTC AATGATTCAA AAAAAAAACT AAGA-GATGG GTGAAATTAC ACAAGTACAC AAGGAATCCT CCGACAAGCT
HapD                 AGCTGCTAGA CTACGTCGAA TAATGAATTC AATGATTCAA AAAAAAAACT AAGA-GATGG GTGAAATTAC ACAAGTACAC AAGGAATCCT CCGACAAGCT
HapA                 AGCTGCTAGA CTACGTCGAA TAATGAATTC AATGATTCAA AAAAAAAACT AAGA-GATGG GTGAAATTAC ACAAGTACAC AAGGAATCCT CCGACAAGCT
HapB                 AGCTGCTAGA CTACGTCGAA TAATGAATTC AATGATTCAA AAAAAAAACT AAGA-GATGG GTGAAATTAC ACAAGTACAC AAGGAATCCT CCGACAAGCT
HapC                 AGCTGCTAGA CTACGTCGAA TAATGAATTC AATGATTCAA AAAAAAAACT AAGA-GATGG GTGAAATTAC ACAAGTACAC AAGGAATCCT CCGACAAGCT
HapY                 AG-------- ---------- ---------- ---------- ---------- ---------- ---------- ---------- ---------- CCGACAAGCT
HapW                 AGCTGCTAGA CTACGTCGAA TAATGAATTC AATGATTCAA AAAAAAAACT AAGA-GATGG GTGAAAT--- -----TACAC AAGGAATCCT CCGACAAGCT
HapX                 AGCTGCTAGA CTACGTCGAA TAATGAATTC AATGATTCAA AAAAAAAACT AAGA-GATGG GTGAAAT--- -----TACAC AAGGAATCCT CCGACAAGCT
HapS                 AGCTGCTAGA CTACGTCGAA TAATGAATTC AATGATTCAA AAAAAAAACT AAGA-GATGG GTGAAATTAC ACAAGTACAC AAGGAATCCT CCGACAAGCT
HaKonecNC_025235     AGCTGCTAGA CTACGTCGAA TAATGAATTC AATGATTCAA AAAAAAAACT AAGA-GATGG GTGAAAT--- -----TACAC AAGGAATCCT CCGACAAGCT
Monparadoxtrnt       AGCTGCTAGA CTACGTCGAA TAATGAATTC AATGATTCAA AAAAAA--CT AAGA-GATGG ATGAAANNNN NNNNNNNNNN NNNNNNNNNN NNNACAAGCT
```

```
                    ....|....| ....|....| ....|....| ....|....| ....|....| ....|....| ....|....| ....|....| ....|....| ....|....|
                          905        915        925        935        945        955        965        975        985        995

HapM                TGCAAATGGA GTCCTGAACT AGCCGCAGCT TGTGAAATAT GGAAGGCGAT CAAATTCGAG TTCGCGCCGG TAGATACCAT CG-------- ----------
HapM1               TGCAAATGGA GTCCTGAACT AGCCGCAGCT TGTGAAATAT GGAAGGCGAT CAAATTCGAG TTCGCGCCGG TAGATACCAT CG-------- ----------
FU12.1              TGCAAATGGA GTCCTGAACT AGCCGCAGCT TGTGAAATAT GGAAGGCGAT CAAATTCGAG TTCGCGCCGG TAGATACCAT CG-------- ----------
FU10.1              TGCAAATGGA GTCCTGAACT AGCCGCAGCT TGTGAAATAT GGAAGGCGAT CAAATTCGAG TTCGCGCCGG TAGATACCAT CG-------- ----------
FU1.1               TGCAAATGGA GTCCTGAACT AGCCGCAGCT TGTGAAATAT GGAAGGCGAT CAAATTCGAG TTCGCGCCGG TAGATACCAT CG-------- ----------
FU2.1               TGCAAATGGA GTCCTGAACT AGCCGCAGCT TGTGAAATAT GGAAGGCGAT CAAATTCGAG TTCGCGCCGG TAGATACCAT CG-------- ----------
FU11.1              TGCAAATGGA GTCCTGAACT AGCCGCAGCT TGTGAAATAT GGAAGGCGAT CAAATTCGAG TTCGCGCCGG TAGATACCAT CG-------- ----------
CO4.1               TGCAAATGGA GTCCTGAACT AGCCGCAGCT TGTGAAATAT GGAAGGCGAT CAAATTCGAG TTCGCGCCGG TAGATACCAT CG-------- ----------
CO11.1              TGCAAATGGA GTCCTGAACT AGCCGCAGCT TGTGAAATAT GGAAGGCGAT CAAATTCGAG TTCGCGCCGG TAGATACCAT CG-------- ----------
TR1.1               TGCAAATGGA GTCCTGAACT AGCCGCAGCT TGTGAAATAT GGAAGGCGAT CAAATTCGAG TTCGCGCCGG TAGATACCAT CG-------- ----------
TR3.1               TGCAAATGGA GTCCTGAACT AGCCGCAGCT TGTGAAATAT GGAAGGCGAT CAAATTCGAG TTCGCGCCGG TAGATACCAT CG-------- ----------
TR10.1              TGCAAATGGA GTCCTGAACT AGCCGCAGCT TGTGAAATAT GGAAGGCGAT CAAATTCGAG TTCGCGCCGG TAGATACCAT CG-------- ----------
TR11.1              TGCAAATGGA GTCCTGAACT AGCCGCAGCT TGTGAAATAT GGAAGGCGAT CAAATTCGAG TTCGCGCCGG TAGATACCAT CG-------- ----------
Vi16.1              TGCAAATGGA GTCCTGAACT AGCCGCAGCT TGTGAAATAT GGAAGGCGAT CAAATTCGAG TTCGCGCCGG TAGATACCAT CG-------- ----------
VI1.1               TGCAAATGGA GTCCTGAACT AGCCGCAGCT TGTGAAATAT GGAAGGCGAT CAAATTCGAG TTCGCGCCGG TAGATACCAT CG-------- ----------
Vi3.1               TGCAAATGGA GTCCTGAACT AGCCGCAGCT TGTGAAATAT GGAAGGCGAT CAAATTCGAG TTCGCGCCGG TAGATACCAT CG-------- ----------
CH1.1               TGCAAATGGA GTCCTGAACT AGCCGCAGCT TGTGAAATAT GGAAGGCGAT CAAATTCGAG TTCGCGCCGG TAGATACCAT CG-------- ----------
CH4.1               TGCAAATGGA GTCCTGAACT AGCCGCAGCT TGTGAAATAT GGAAGGCGAT CAAATTCGAG TTCGCGCCGG TAGATACCAT CG-------- ----------
CH6.1               TGCAAATGGA GTCCTGAACT AGCCGCAGCT TGTGAAATAT GGAAGGCGAT CAAATTCGAG TTCGCGCCGG TAGATACCAT CG-------- ----------
CH2.2               TGCAAATGGA GTCCTGAACT AGCCGCAGCT TGTGAAATAT GGAAGGCGAT CAAATTCGAG TTCGCGCCGG TAGATACCAT CG-------- ----------
CH2.1               TGCAAATGGA GTCCTGAACT AGCCGCAGCT TGTGAAATAT GGAAGGCGAT CAAATTCGAG TTCGCGCCGG TAGATACCAT CG-------- ----------
HapAD               TGCAAATGGA GTCCTGAACT AGCCGCAGCT TGTGAAATAT GGAAGGCGAT CAAATTCGAG TTCGCGCCGG TAGATACCAT CG-------- ----------
HapO                TGCAAATGGA GTCCTGAACT AGCCGCAGCT TGTGAAATAT GGAAGGCGAT CAAATTCGAG TTCGCGCCGG TAGATACCAT CG-------- ----------
HapN                TGCAAATGGA GTCCTGAACT AGCCGCAGCT TGTGAAATAT GGAAGGCGAT CAAATTCGAG TTCGCGCCGG TAGATACCAT CG-------- ----------
HapK                TGCAAATGGA GTCCTGAACT AGCCGCAGCT TGTGAAATAT GGAAGGCGAT CAAATTCGAG TTCGCGCCGG TAGATACCAT CG-------- ----------
HapJ                TGCAAATGGA GTCCTGAACT AGCCGCAGCT TGTGAAATAT GGAAGGCGAT CAAATTCGAG TTCGCGCCGG TAGATACCAT CG-------- ----------
HapL                TGCAAATGGA GTCCTGAACT AGCCGCAGCT TGTGAAATAT GGAAGGCGAT CAAATTCGAG TTCGCGCCGG TAGATACCAT CG-------- ----------
HapQ                TGCAAATGGA GTCCTGAACT AGCCGCAGCT TGTGAAATAT GGAAGGCGAT CAAATTCGAG TTCGAGCCGG TAGATACCAT CG-------- ----------
HapV                TGCAAATGGA GTCCTGAACT AGCCGCAGCT TGTGAAATAT GGAAGGCGAT CAAATTCGAG TTCGAGCCGG TAGATACCAT CG-------- ----------
HapR                TGCAAATGGA GTCCTGAACT AGCCGCAGCT TGTGAAATAT GGAAGGCGAT CAAATTCGAG TTCGAGCCGG TAGATACCAT CG-------- ----------
HapP                TGCAAATGGA GTCCTGAACT AGCCGCAGCT TGTGAAATAT GGAAGGCGAT CAAATTCGAG TTCGAGCCGG TAGATACCAT CG-------- ----------
HapU                TGCAAATGGA GTCCTGAACT AGCCGCAGCT TGTGAAATAT GGAAGGCGAT CAAATTCGAG TTCGAGCCGG TAGATACCAT CG-------- ----------
HapT                TGCAAATGGA GTCCTGAACT AGCCGCAGCT TGTGAAATAT GGAAGGCGAT CAAATTCGAG TTCGCGCCGG TAGATACCAT CG-------- ----------
HapAA               ---------- ---------- ---------- ----AAATAT GGAAGGCGAT CAAATTCGAG TTCGAGCCGG TAGATACCAT CGATTAAGTA GATAAAACTA
HapH30              TGCAAATGGA GTCCTGAACT AGCCGCAGCT TGTGAAATAT GGAAGGCGAT CAAATTCGAG TTCGAGCCGG TAGATACCAT CG-------- ----------
HapI                TGCAAATGGA GTCCTGAACT AGCCGCAGCT TGTGAAATAT GGAAGGCGAT CAAATTCGAG TTCGAGCCGG TAGATACCAT CG-------- ----------
HapE                TGCAAATGGA GTCCTGAACT AGCCGCAGCT TGTGAAATAT GGAAGGCGAT CAAATTCGAG TTCGAGCCGG TAGATACCAT CG-------- ----------
HapAC               TGCAAATGGA GTCCTGAACT AGCCGCAGCT TGTGAAATAT GGAAGGCGAT CAAATTCGAG TTCGAGCCGG TAGATACCAT CG-------- ----------
HapF                TGCAAATGGA GTCCTGAACT AGCCGCAGCT TGTGAAATAT GGAAGGCGAT CAAATTCGAG TTCGAGCCGG TAGATACCAT CG-------- ----------
HapH                TGCAAATGGA GTCCTGAACT AGCCGCAGCT TGTGAAATAT GGAAGGCGAT CAAATTCGAG TTCGAGCCGG TAGATACCAT CG-------- ----------
HapG                TGCAAATGGA GTCCTGAACT AGCCGCAGCT TGTGAAATAT GGAAGGCGAT CAAATTCGAG TTCGAGCCGG TAGATACCAT CG-------- ----------
HapAB               TGCAAATGGA GTCCTGAACT AGCCGCAGCT TGTGAAATAT GGAAGGCGAT CAAATTCGAG TTCGAGCCGG TAGATACCAT CG-------- ----------
HapZ                TGCAAATGGA GTCCTGAACT AGCCGCAGCT TGTGAAATAT GGAAGGCGAT CAAATTCGAG TTCGAGCCGG TAGATACCAT CG-------- ----------
HapD                TGCAAATGGA GTCCTGAACT AGCCGCAGCT TGTGAAATAT GGAAGGCGAT CAAATTCGAG TTCGAGCCGG TAGATACCAT CG-------- ----------
HapA                TGCAAATGGA GTCCTGAACT AGCCGCAGCT TGTGAAATAT GGAAGGCGAT CAAATTCGAG TTCGAGCCGG TAGATACCAT CG-------- ----------
HapB                TGCAAATGGA GTCCTGAACT AGCCGCAGCT TGTGAAATAT GGAAGGCGAT CAAATTCGAG TTCGAGCCGG TAGATACCAT CG-------- ----------
HapC                TGCAAATGGA GTCCTGAACT AGCCGCAGCT TGTGAAATAT GGAAGGCGAT CAAATTCGAG TTCGAGCCGG TAGATACCAT CG-------- ----------
HapY                TGCAAATGGA GTCCTGAACT AGCCGCAGCT TGTGAAATAT GGAAGGCGAT CAAATTCGAG TTCGAGCCGG TAGATACCAT CG-------- ----------
HapW                TGCAAATGGA GTCCTGAACT AGCCGCAGCT TGTGAAATAT GGAAGGCGAT CAAATTCGAG TTCGCGCCGG TAGATACCAT CG-------- ----------
HapX                TGCAAATGGA GTCCTGAACT AGCCGCAGCT TGTGAAATAT GGAAGGCGAT CAAATTCGAG TTCGCGCCGG TAGATACCAT CG-------- ----------
HapS                TGCAAATGGA GTCCTGAACT AGCCGCAGCT TGTGAAATAT GGAAGGCGAT CAAATTCGAG TTCGAGCCGG TAGATACCAT CG-------- ----------
HaKonecNC_025235    TGCAAATGGA GTCCTGAACT AGCCGCAGCT TGTGAAGTAT GGAAGGCGAT CACATTCGAT TTCGCGCCGG TAGATACCAT CG-------- ----------
Monparadoxtrnt      TGCAAATGGA GTCCTGAACT AGCCGCAGCT TGTGAAGTAT GGAAGGCGAT CAAATTCGAG TTCAAGCCGG TAGATACCAT CG-------- ----------

                    ....|....| ....|....| ....|....| ....|....| ....|....| ....|....| ....|....| ....|....| ....|....| ....|....|
                         1005       1015       1025       1035       1045       1055       1065       1075       1085       1095

HapM                -----ATTAA GTAGATAAAA CTAGATATAA AGAAGGTCTA AAAAAAAAAG AAGCGAAATA GAAA------ -AAAAATCAG TTACGAAATG CAGTAATTCT
HapM1               -----ATTAA GTAGATAAAA CTAGATATAA AGAAGGTCTA AAAAAAAAAG AAGCGAAATA GAAA------ -AAAAATCAG TTACGAAATG CAGTAATTCT
FU12.1              -----ATTAA GTAGATAAAA CTAGATATAA AGAAGGTCTA AAAAAAAAAG AAGCGAAATA GAAA------ -AAAAATCAG TTACGAAATG CAGTAATTCT
FU10.1              -----ATTAA GTAGATAAAA CTAGATATAA AGAAGGTCTA AAAAAAAAAG AAGCGAAATA GAAA------ -AAAAATCAG TTACGAAATG CAGTAATTCT
FU1.1               -----ATTAA GTAGATAAAA CTAGATATAA AGAAGGTCTA AAAAAAAAAG AAGCGAAATA GAAA------ -AAAAATCAG TTACGAAATG CAGTAATTCT
FU2.1               -----ATTAA GTAGATAAAA CTAGATATAA AGAAGGTCTA AAAAAAAAAG AAGCGAAATA GAAA------ -AAAAATCAG TTACGAAATG CAGTAATTCT
FU11.1              -----ATTAA GTAGATAAAA CTAGATATAA AGAAGGTCTA AAAAAAAAAG AAGCGAAATA GAAA------ -AAAAATCAG TTACGAAATG CAGTAATTCT
CO4.1               -----ATTAA GTAGATAAAA CTAGATATAA AGAAGGTCTA AAAAAAAAAG AAGCGAAATA GAAA------ -AAAAATCAG TTACGAAATG CAGTAATTCT
CO11.1              -----ATTAA GTAGATAAAA CTAGATATAA AGAAGGTCTA AAAAAAAAAG AAGCGAAATA GAAA------ -AAAAATCAG TTACGAAATG CAGTAATTCT
TR1.1               -----ATTAA GTAGATAAAA CTAGATATAA AGAAGGTCTA AAAAAAAAAG AAGCGAAATA GAAA------ -AAAAATCAG TTACGAAATG CAGTAATTCT
TR3.1               -----ATTAA GTAGATAAAA CTAGATATAA AGAAGGTCTA AAAAAAAAAG AAGCGAAATA GAAA------ -AAAAATCAG TTACGAAATG CAGTAATTCT
TR10.1              -----ATTAA GTAGATAAAA CTAGATATAA AGAAGGTCTA AAAAAAAAAG AAGCGAAATA GAAA------ -AAAAATCAG TTACGAAATG CAGTAATTCT
TR11.1              -----ATTAA GTAGATAAAA CTAGATATAA AGAAGGTCTA AAAAAAAAAG AAGCGAAATA GAAA------ -AAAAATCAG TTACGAAATG CAGTAATTCT
Vi16.1              -----ATTAA GTAGATAAAA CTAGATATAA AGAAGGTCTA AAAAAAAAAG AAGCGAAATA GAAA------ -AAAAATCAG TTACGAAATG CAGTAATTCT
VI1.1               -----ATTAA GTAGATAAAA CTAGATATAA AGAAGGTCTA AAAAAAAAAG AAGCGAAATA GAAA------ -AAAAATCAG TTACGAAATG CAGTAATTCT
Vi3.1               -----ATTAA GTAGATAAAA CTAGATATAA AGAAGGTCTA AAAAAAAAAG AAGCGAAATA GAAA------ -AAAAATCAG TTACGAAATG CAGTAATTCT
CH1.1               -----ATTAA GTAGATAAAA CTAGATATAA AGAAGGTCTA AAAAAAAAAG AAGCGAAATA GAAA------ -AAAAATCAG TTACGAAATG CAGTAATTCT
CH4.1               -----ATTAA GTAGATAAAA CTAGATATAA AGAAGGTCTA AAAAAAAAAG AAGCGAAATA GAAA------ -AAAAATCAG TTACGAAATG CAGTAATTCT
CH6.1               -----ATTAA GTAGATAAAA CTAGATATAA AGAAGGTCTA AAAAAAAAAG AAGCGAAATA GAAA------ -AAAAATCAG TTACGAAATG CAGTAATTCT
CH2.2               -----ATTAA GTAGATAAAA CTAGATATAA AGAAGGTCTA AAAAAAAAAG AAGCGAAATA GAAA------ -AAAAATCAG TTACGAAATG CAGTAATTCT
CH2.1               -----ATTAA GTAGATAAAA CTAGATATAA AGAAGGTCTA AAAAAAAAAG AAGCGAAATA GAAA------ -AAAAATCAG TTACGAAATG CAGTAATTCT
HapAD               -----ATTAA GTAGATAAAA CTAGATATAA AGAAGGTCTA AAAAAAAAAG AAGCGAAATA GAAA------ -AAAAATCAG TTACGAAATG CAGTAATTCT
HapO                -----ATTAA GTAGATAAAA CTAGATATAA AGAAGGTCTA AAAAAAAAAG AAGCGAAATA GAAA------ -AAAAATCAG TTACGAAATG CAGTAATTCT
HapN                -----ATTAA GTAGATAAAA CTAGATATAA AGAAGGTCTA AAAAAAAAAG AAGCGAAATA GAAA------ -AAAAATCAG TTACGAAATG CAGTAATTCT
HapK                -----ATTAA GTAGATAAAA CTAGATATAA AGAAGGTCTA AAAAAAAAAG AAGCGAAATA GAAA------ -AAAAATCAG TTACGAAATG CAGTAATTCT
HapJ                -----ATTAA GTAGATAAAA CTAGATATAA AGAAGGTCTA AAAAAAAAAG AAGCGAAATA GAAA------ -AAAAATCAG TTACGAAATG CAGTAATTCT
HapL                -----ATTAA GTAGATAAAA CTAGATATAA AGAAGGTCTA AAAAAAAAAG AAGCGAAATA GAAA------ -AAAAATCAG TTACGAAATG CAGTAATTCT
HapQ                -----ATTAA GTAGATAAAA CTAGATATAA AGAAGGTCTA AAAAAAAAAG AAGCGAAATA GAAA------ -AAAAATCAG TTACGAAATG CAGTAATTCT
HapV                -----ATTAA GTAGATAAAA CTAGATATAA AGAAGGTCTA AAAAAAAAAG AAGCGAAATA GAAA------ -AAAAATCAG TTACGAAATG CAGTAATTCT
HapR                -----ATTAA GTAGATAAAA CTAGATATAA AGAAGGTCTA AAAAAAAAAG AAGCGAAATA GAAA------ -AAAAATCAG TTACGAAATG CAGTAATTCT
HapP                -----ATTAA GTAGATAAAA CTAGATATAA AGAAGGTCTA AAAAAAAAAG AAGCGAAATA GAAA------ -AAAAATCAG TTACGAAATG CAGTAATTCT
HapU                -----ATTAA GTAGATAAAA CTAGATATAA AGAAGGTCTA AAAAAAAAAG AAGCGAAATA GAAA------ -AAAAATCAG TTACGAAATG CAGTAATTCT
HapT                -----ATTAA GTAGATAAAA CTAGATATAA AGAAGGTCTA AAAAAAAAAG AAGCGAAATA GAAA------ -AAAAATCAG TTACGAAATG CAGTAATTCT
HapAA               GATATATTAA GTAGATAAAA CTAGATATAA AGAAGGTCTA AAAAAAAAAG AAGCGAAATA GAAA------ -AAAAATCAG TTACGAAATG CAGTAATTCT
HapH30              -----ATTAA GTAGATAAAA CTAGATATAA AGAAGGTCTA AAAAAAAAAG AAGCGAAATA GAAA------ -AAAAATCAG TTACGAAATG CAGTAATTCT
HapI                -----ATTAA GTAGATAAAA CTAGATATAA AGAAGGTCTA AAAAAAAAAG AAGCGAAATA GAAA------ -AAAAATCAG TTACGAAATG CAGTAATTCT
HapE                -----ATTAA GTAGATAAAA CTAGATATAA AGAAGGTCTA AAAAAAAAAG AAGCGAAATA GAAA------ -AAAAATCAG TTACGAAATG CAGTAATTCT
HapAC               -----ATTAA GTAGATAAAA CTAGATATAA AGAAGGTCTA AAAAAAAAAG AAGCGAAATA GAAA------ -AAAAATCAG TTACGAAATG CAGTAATTCT
HapF                -----ATTAA GTAGATAAAA CTAGATATAA AGAAGGTCTA AAAAAAAAAG AAGCGAAATA GAAA------ -AAAAATCAG TTACGAAATG CAGTAATTCT
HapH                -----ATTAA GTAGATAAAA CTAGATATAA AGAAGGTCTA AAAAAAAAAG AAGCGAAATA GAAA------ -AAAAATCAG TTACGAAATG CAGTAATTCT
HapG                -----ATTAA GTAGATAAAA CTAGATATAA AGAAGGTCTA AAAAAAAAAG AAGCGAAATA GAAA------ -AAAAATCAG TTACGAAATG CAGTAATTCT
HapAB               -----ATTAA GTAGATAAAA CTAGATATAA AGAAGGTCTA AAAAAAAAAG AAGCGAAATA GAAA------ -AAAAATCAG TTACGAAATG CAGTAATTCT
HapZ                -----ATTAA GTAGATAAAA CTAGATATAA AGAAGGTCTA AAAAAAAAAG AAGCGAAATA GAAA------ -AAAAATCAG TTACGAAATG CAGTAATTCT
HapD                -----ATTAA GTAGATAAAA CTAGATATAA AGAAGGTCTA AAAAAAAAAG AAGCGAAATA GAAA------ -AAAAATCAG TTACGAAATG CAGTAATTCT
HapA                -----ATTAA GTAGATAAAA CTAGATATAA AGAAGGTCTA AAAAAAAAAG AAGCGAAATA GAAA------ -AAAAATCAG TTACGAAATG CAGTAATTCT
HapB                -----ATTAA GTAGATAAAA CTAGATATAA AGAAGGTCTA AAAAAAAAAG AAGCGAAATA GAAA------ -AAAAATCAG TTACGAAATG CAGTAATTCT
HapC                -----ATTAA GTAGATAAAA CTAGATATAA AGAAGGTCTA AAAAAAAAAG AAGCGAAATA GAAA------ -AAAAATCAG TTACGAAATG CAGTAATTCT
HapY                -----ATTAA GTAGATAAAA CTAGATATAA AGAAGGTCTA AAAAAAAAAG AAGCGAAATA GAAA------ -AAAAATCAG TTACGAAATG CAGTAATTCT
HapW                -----ATTAA GTAGATAAAA CTAGATATAA AGAAGGTCTA AAAAAAAAAG AAGCGAAATA GAAA------ -AAAAATCAG TTACGAAATG CAGTAATTCT
HapX                -----ATTAA GTAGATAAAA CTAGATATAA AGAAGGTCTA AAAAAAAAAG AAGCGAAATA GAAA------ -AAAAATCAG TTACGAAATG CAGTAATTCT
HapS                -----ATTAA GTAGATAAAA CTAGATATAA AGAAGGTCTA AAAAAAAAAG AAGCGAAATA GAAA------ -AAAAATCAG TTACGAAATG CAGTAATTCT
HaKonecNC_025235    -----ATTAA GTAGATAAAA CTAGATATAA AGAAGGTCTA AAAAAAAA-G AAGCGAAATA GAAA------ --AAAATCAG TTACGAAATG CAGTAATTCT
Monparadoxtrnt      -----ATTAA GTAGATAAAA CTAGATATAA AGAAGGTCTA AATAAAAAAG AAGCAAAATA GAAAGAAAGA AAAAAATCAG TTACGAAATG CAGTAATTCT

                    ....|....| ....|....| ....|....| ....|....| ....|....| ....|....| ....|....| ....|....| ....|....| ....|....|
                         1105       1115       1125       1135       1145       1155       1165       1175       1185       1195

HapM                TCTTTATTCT TCTAATTGAT TGCAATGAAA TTCGGCTCAA TC-----TTT TTCG------ -AAAAAGATT GAGCCGAATT TCAATAGATC TTGATACGAT
HapM1               TCTTTATTCT TCTAATTGAT TGCAATGAAA TTCGGCTCAA TC-----TTT TTCG------ -AAAAAGATT GAGCCGAATT TCAATAGATC TTGATACGAT
FU12.1              TCTTTATTCT TCTAATTGAT TGCAATGAAA TTCGGCTCAA TC-----TTT TTCG------ -AAAAAGATT GAGCCGAATT TCAATAGATC TTGATACGAT
FU10.1              TCTTTATTCT TCTAATTGAT TGCAATGAAA TTCGGCTCAA TC-----TTT TTCG------ -AAAAAGATT GAGCCGAATT TCAATAGATC TTGATACGAT
FU1.1               TCTTTATTCT TCTAATTGAT TGCAATGAAA TTCGGCTCAA TC-----TTT TTCG------ -AAAAAGATT GAGCCGAATT TCAATAGATC TTGATACGAT
FU2.1               TCTTTATTCT TCTAATTGAT TGCAATGAAA TTCGGCTCAA TC-----TTT TTCG------ -AAAAAGATT GAGCCGAATT TCAATAGATC TTGATACGAT
FU11.1              TCTTTATTCT TCTAATTGAT TGCAATGAAA TTCGGCTCAA TC-----TTT TTCG------ -AAAAAGATT GAGCCGAATT TCAATAGATC TTGATACGAT
CO4.1               TCTTTATTCT TCTAATTGAT TGCAATGAAA TTCGGCTCAA TC-----TTT TTCG------ -AAAAAGATT GAGCCGAATT TCAATAGATC TTGATACGAT
CO11.1              TCTTTATTCT TCTAATTGAT TGCAATGAAA TTCGGCTCAA TC-----TTT TTCG------ -AAAAAGATT GAGCCGAATT TCAATAGATC TTGATACGAT
TR1.1               TCTTTATTCT TCTAATTGAT TGCAATGAAA TTCGGCTCAA TC-----TTT TTCG------ -AAAAAGATT GAGCCGAATT TCAATAGATC TTGATACGAT
TR3.1               TCTTTATTCT TCTAATTGAT TGCAATGAAA TTCGGCTCAA TC-----TTT TTCG------ -AAAAAGATT GAGCCGAATT TCAATAGATC TTGATACGAT
TR10.1              TCTTTATTCT TCTAATTGAT TGCAATGAAA TTCGGCTCAA TC-----TTT TTCG------ -AAAAAGATT GAGCCGAATT TCAATAGATC TTGATACGAT
TR11.1              TCTTTATTCT TCTAATTGAT TGCAATGAAA TTCGGCTCAA TC-----TTT TTCG------ -AAAAAGATT GAGCCGAATT TCAATAGATC TTGATACGAT
Vi16.1              TCTTTATTCT TCTAATTGAT TGCAATGAAA TTCGGCTCAA TC-----TTT TTCG------ -AAAAAGATT GAGCCGAATT TCAATAGATC TTGATACGAT
VI1.1               TCTTTATTCT TCTAATTGAT TGCAATGAAA TTCGGCTCAA TC-----TTT TTCG------ -AAAAAGATT GAGCCGAATT TCAATAGATC TTGATACGAT
Vi3.1               TCTTTATTCT TCTAATTGAT TGCAATGAAA TTCGGCTCAA TC-----TTT TTCG------ -AAAAAGATT GAGCCGAATT TCAATAGATC TTGATACGAT
CH1.1               TCTTTATTCT TCTAATTGAT TGCAATGAAA TTCGGCTCAA TC-----TTT TTCG------ -AAAAAGATT GAGCCGAATT TCAATAGATC TTGATACGAT
CH4.1               TCTTTATTCT TCTAATTGAT TGCAATGAAA TTCGGCTCAA TC-----TTT TTCG------ -AAAAAGATT GAGCCGAATT TCAATAGATC TTGATACGAT
CH6.1               TCTTTATTCT TCTAATTGAT TGCAATGAAA TTCGGCTCAA TC-----TTT TTCG------ -AAAAAGATT GAGCCGAATT TCAATAGATC TTGATACGAT
CH2.2               TCTTTATTCT TCTAATTGAT TGCAATGAAA TTCGGCTCAA TC-----TTT TTCG------ -AAAAAGATT GAGCCGAATT TCAATAGATC TTGATACGAT
CH2.1               TCTTTATTCT TCTAATTGAT TGCAATGAAA TTCGGCTCAA TC-----TTT TTCG------ -AAAAAGATT GAGCCGAATT TCAATAGATC TTGATACGAT
HapAD               TCTTTATTCT TCTAATTGAT TGCAATGAAA TTCGGCTCAA TC-----TTT TTCG------ -AAAAAGATT GAGCCGAATT TCAATAGATC TTGATACGAT
HapO                TCTTTATTCT TCTAATTGAT TGCAATGAAA TTCGGCTCAA TC-----TTT TTCG------ -AAAAAGATT GAGCCGAATT TCAATAGATC TTGATACGAT
HapN                TCTTTATTCT TCTAATTGAT TGCAATGAAA TTCGGCTCAA TC-----TTT TTCG------ -AAAAAGATT GAGCCGAATT TCAATAGATC TTGATACGAT
HapK                TCTTTATTCT TCTAATTGAT TGCAATGAAA TTCGGCTCAA TC-----TTT TTCG------ -AAAAAGATT GAGCCGAATT TCAATAGATC TTGATACGAT
HapJ                TCTTTATTCT TCTAATTGAT TGCAATGAAA TTCGGCTCAA TC-----TTT TTCG------ -AAAAAGATT GAGCCGAATT TCAATAGATC TTGATACGAT
HapL                TCTTTATTCT TCTAATTGAT TGCAATGAAA TTCGGCTCAA TC-----TTT TTCG------ -AAAAAGATT GAGCCGAATT TCAATAGATC TTGATACGAT
HapQ                TCTTTATTCT TCTAATTGAT TGCAATGAAA TTCGGCTCAA TC-----TTT TTCG------ -AAAAAGATT GAGCCGAATT TCAATAGATC TTGATACGAT
HapV                TCTTTATTCT TCTAATTGAT TGCAATGAAA TTCGGCTCAA TC-----TTT TTCG------ -AAAAAGATT GAGCCGAATT TCAATAGATC TTGATACGAT
HapR                TCTTTATTCT TCTAATTGAT TGCAATGAAA TTCGGCTCAA TC-----TTT TTCG------ -AAAAAGATT GAGCCGAATT TCAATAGATC TTGATACGAT
HapP                TCTTTATTCT TCTAATTGAT TGCAATGAAA TTCGGCTCAA TC-----TTT TTCG------ -AAAAAGATT GAGCCGAATT TCAATAGATC TTGATACGAT
HapU                TCTTTATTCT TCTAATTGAT TGCAATGAAA TTCGGCTCAA TCTTTTTTTT TTCG------ -AAAAAGATT GAGCCGAATT TCAATAGATC TTGATACGAT
HapT                TCTTTATTCT TCTAATTGAT TGCAATGAAA TTCGGCTCAA TC-----TTT TTCG------ -AAAAAGATT GAGCCGAATT TCAATAGATC TTGATACGAT
HapAA               TCTTTATTCT TCTAATTGAT TGCAATGAAA TTCGGCTCAA TC-----TTT TTCG------ -AAAAAGATT GAGCCGAATT TCAATAGATC TTGATACGAT
HapH30              TCTTTATTCT TCTAATTGAT TGCAATGAAA TTCGGCTCAA TC-----TTT TTCG------ -AAAAAGATT GAGCCGAATT TCAATAGATC TTGATACGAT
HapI                TCTTTATTCT TCTAATTGAT TGCAATGAAA TTCGGCTCAA TC-----TTT TTCG--AAAA AAAAAAGATT GAGCCGAATT TCAATAGATC TTGATACGAT
HapE                TCTTTATTCT TCTAATTGAT TGCAATGAAA TTCGGCTCAA TC-----TTT TTCG------ -AAAAAGATT GAGCCGAATT TCAATAGATC TTGATACGAT
HapAC               TCTTTATTCT TCTAATTGAT TGCAATGAAA TTCGGCTCAA TC-----TTT TTCG------ -AAAAAGATT GAGCCGAATT TCAATAGATC TTGATACGAT
HapF                TCTTTATTCT TCTAATTGAT TGCAATGAAA TTCGGCTCAA TC-----TTT TTCG------ -AAAAAGATT GAGCCGAATT TCAATAGATC TTGATACGAT
HapH                TCTTTATTCT TCTAATTGAT TGCAATGAAA TTCGGCTCAA TC-----TTT TTCG------ -AAAAAGATT GAGCCGAATT TCAATAGATC TTGATACGAT
HapG                TCTTTATTCT TCTAATTGAT TGCAATGAAA TTCGGCTCAA TC-----TTT TTCG------ -AAAAAGATT GAGCCGAATT TCAATAGATC TTGATACGAT
HapAB               TCTTTATTCT TCTAATTGAT TGCAATGAAA TTCGGCTCAA TC-----TTT TTCG------ -AAAAAGATT GAGCCGAATT TCAATAGATC TTGATACGAT
HapZ                TCTTTATTCT TCTAATTGAT TGCAATGAAA TTCGGCTCAA TC-----TTT TTCG------ -AAAAAGATT GAGCCGAATT TCAATAGATC TTGATACGAT
HapD                TCTTTATTCT TCTAATTGAT TGCAATGAAA TTCGGCTCAA TC-----TTT TTCG------ -AAAAAGATT GAGCCGAATT TCAATAGATC TTGATACGAT
HapA                TCTTTATTCT TCTAATTGAT TGCAATGAAA TTCGGCTCAA TC-----TTT TTCG------ -AAAAAGATT GAGCCGAATT TCAATAGATC TTGATACGAT
HapB                TCTTTATTCT TCTAATTGAT TGCAATGAAA TTCGGCTCAA TC-----TTT TTCG------ -AAAAAGATT GAGCCGAATT TCAATAGATC TTGATACGAT
HapC                TCTTTATTCT TCTAATTGAT TGCAATGAAA TTCGGCTCAA TC-----TTT TTCG------ -AAAAAGATT GAGCCGAATT TCAATAGATC TTGATACGAT
HapY                TCTTTATTCT TCTAATTGAT TGCAATGAAA TTCGGCTCAA TC-----TTT TTCG--AAAA AAAAAAGATT GAGCCGAATT TCAATAGATC TTGATACGAT
HapW                TCTTTATTCT TCTAATTGAT TGCAATGAAA TTCGGCTCAA TC-----TTT TTCG------ -AAAAAGATT GAGCCGAATT TCAATAGATC TTGATACGAT
HapX                TCTTTATTCT TCTAATTGAT TGCAATGAAA TTCGGCTCAA TC-----TTT TTCG------ -AAAAAGATT GAGCCGAATT TCAATAGATC TTGATACGAT
HapS                TCTTTATTCT TCTAATTGAT TGCAATGAAA TTCGGCTCAA TC-----TTT TTCG------ -AAAAAGATT GAGCCGAATT TCAATAGATC TTGATACGAT
HaKonecNC_025235    TCTTTATTCT TCTAATTGAT TGCAATGAAA TTCGGCTCAA TC--TTTTTT TTCT---AAA AAAAAAGATT GAGCTGAATT TCAATAGATC TTGATACGAT
Monparadoxtrnt      TCTTTATTCT TCTAATTGAT TGCAATTAAA TTCAGCTCAA TC-----TTT TTCTAAAAAA AAAAAAGATT GAGCTGAATT TAAATAGATC TTGATACGAT
```

```
                       ....|....| ....|....| ....|....| ....|....| ....|....| ....|....| ....|....| ....|....| ....|....| ....|....|
                            1205       1215       1225       1235       1245       1255       1265       1275       1285       1295

HapM                   CATGAGACTT GACAAATCGA GATTCTTCTA TTCTATATAT CTAGAATATA GA-AAGGTAT AATACAATAA ACAAATACAA ATAAAAATAT AGTATTATCA
HapM1                  CATGAGACTT GACAAATCGA GATTCTTCTA TTCTATATAT CTAGAATATA GA-AAGGTAT AATACAATAA ACAAATACAA ATAAAAATAT AGTATTATCA
FU12.1                 CATGAGACTT GACAAATCGA GATTCTTCTA TTCTATATAT CTAGAATATA GA-AAGGTAT AATACAATAA ACAAATACAA ATAAAAATAT AGTATTATCA
FU10.1                 CATGAGACTT GACAAATCGA GATTCTTCTA TTCTATATAT CTAGAATATA GA-AAGGTAT AATACAATAA ACAAATACAA ATAAAAATAT AGTATTATCA
FU1.1                  CATGAGACTT GACAAATCGA GATTCTTCTA TTCTATATAT CTAGAATATA GA-AAGGTAT AATACAATAA ACAAATACAA ATAAAAATAT AGTATTATCA
FU2.1                  CATGAGACTT GACAAATCGA GATTCTTCTA TTCTATATAT CTAGAATATA GA-AAGGTAT AATACAATAA ACAAATACAA ATAAAAATAT AGTATTATCA
FU11.1                 CATGAGACTT GACAAATCGA GATTCTTCTA TTCTATATAT CTAGAATATA GA-AAGGTAT AATACAATAA ACAAATACAA ATAAAAATAT AGTATTATCA
CO4.1                  CATGAGACTT GACAAATCGA GATTCTTCTA TTCTATATAT CTAGAATATA GA-AAGGTAT AATACAATAA ACAAATACAA ATAAAAATAT AGTATTATCA
CO11.1                 CATGAGACTT GACAAATCGA GATTCTTCTA TTCTATATAT CTAGAATATA GA-AAGGTAT AATACAATAA ACAAATACAA ATAAAAATAT AGTATTATCA
TR1.1                  CATGAGACTT GACAAATCGA GATTCTTCTA TTCTATATAT CTAGAATATA GA-AAGGTAT AATACAATAA ACAAATACAA ATAAAAATAT AGTATTATCA
TR3.1                  CATGAGACTT GACAAATCGA GATTCTTCTA TTCTATATAT CTAGAATATA GA-AAGGTAT AATACAATAA ACAAATACAA ATAAAAATAT AGTATTATCA
TR10.1                 CATGAGACTT GACAAATCGA GATTCTTCTA TTCTATATAT CTAGAATATA GA-AAGGTAT AATACAATAA ACAAATACAA ATAAAAATAT AGTATTATCA
TR11.1                 CATGAGACTT GACAAATCGA GATTCTTCTA TTCTATATAT CTAGAATATA GA-AAGGTAT AATACAATAA ACAAATACAA ATAAAAATAT AGTATTATCA
Vi16.1                 CATGAGACTT GACAAATCGA GATTCTTCTA TTCTATATAT CTAGAATATA GA-AAGGTAT AATACAATAA ACAAATACAA ATAAAAATAT AGTATTATCA
VI1.1                  CATGAGACTT GACAAATCGA GATTCTTCTA TTCTATATAT CTAGAATATA GA-AAGGTAT AATACAATAA ACAAATACAA ATAAAAATAT AGTATTATCA
Vi3.1                  CATGAGACTT GACAAATCGA GATTCTTCTA TTCTATATAT CTAGAATATA GA-AAGGTAT AATACAATAA ACAAATACAA ATAAAAATAT AGTATTATCA
CH1.1                  CATGAGACTT GACAAATCGA GATTCTTCTA TTCTATATAT CTAGAATATA GA-AAGGTAT AATACAATAA ACAAATACAA ATAAAAATAT AGTATTATCA
CH4.1                  CATGAGACTT GACAAATCGA GATTCTTCTA TTCTATATAT CTAGAATATA GA-AAGGTAT AATACAATAA ACAAATACAA ATAAAAATAT AGTATTATCA
CH6.1                  CATGAGACTT GACAAATCGA GATTCTTCTA TTCTATATAT CTAGAATATA GA-AAGGTAT AATACAATAA ACAAATACAA ATAAAAATAT AGTATTATCA
CH2.2                  CATGAGACTT GACAAATCGA GATTCTTCTA TTCTATATAT CTAGAATATA GA-AAGGTAT AATACAATAA ACAAATACAA ATAAAAATAT AGTATTATCA
CH2.1                  CATGAGACTT GACAAATCGA GATTCTTCTA TTCTATATAT CTAGAATATA GA-AAGGTAT AATACAATAA ACAAATACAA ATAAAAATAT AGTATTATCA
HapAD                  CATGAGACTT GACAAATCGA GATTCTTCTA TTCTATATAT CTAGAATATA GA-AAGGTAT AATACAATAA ACAAATACAA ATAAAAATAT AGTATTATCA
HapO                   CATGAGACTT GACAAATCGA GATTCTTCTA TTCTATATAT CTAGAATATA GA-AAGGTAT AATACAATAA ACAAATACAA ATAAAAATAT AGTATTATCA
HapN                   CATGAGACTT GACAAATCGA GATTCTTCTA TTCTATATAT CTAGAATATA GA-AAGGTAT AATACAATAA ACAAATACAA ATAAAAATAT AGTATTATCA
HapK                   CATGAGACTT GACAAATCGA GATTCTTCTA TTCTATATAT CTAGAATATA GA-AAGGTAT AATACAATAA ACAAATACAA ATAAAAATAT AGTATTATCA
HapJ                   CATGAGACTT GACAAATCGA GATTCTTCTA TTCTATATAT CTAGAATATA GA-AAGGTAT AATACAATAA ACAAATACAA ATAAAAATAT AGTATTATCA
HapL                   CATGAGACTT GACAAATCGA GATTCTTCTA TTCTATATAT CTAGAATATA GA-AAGGTAT AATACAATAA ACAAATACAA ATAAAAATAT AGTATTATCA
HapQ                   CATGAGACTT GACAAATCGA GATTCTTCTA TTCTATATAT CTAGAATATA GA-AAGGTAT AATACAATAA ACAAATACAA ATAAAAATAT AGTATTATCA
HapV                   CATGAGACTT GACAAATCGA GATTCTTCTA TTCTATATAT CTAGAATATA GA-AAGGTAT AATACAATAA ACAAATACAA ATAAAAATAT AGTATTATCA
HapR                   CATGAGACTT GACAAATCGA GATTCTTCTA TTCTATATAT CTAGAATATA GA-AAGGTAT AATACAATAA ACAAATACAA ATAAAAATAT AGTATTATCA
HapP                   CATGAGACTT GACAAATCGA GATTCTTCTA TTCTATATAT CTAGAATATA GA-AAGGTAT AATACAATAA ACAAATACAA ATAAAAATAT AGTATTATCA
HapU                   CATGAGACTT GACAAATCGA GATTCTTCTA TTCTATATAT CTAGAATATA GA-AAGGTAT AATACAATAA ACAAATACAA ATAAAAATAT AGTATTATCA
HapT                   CATGAGACTT GACAAATCGA GATTCTTCTA TTCTATATAT CTAGAATATA GA-AAGGTAT AATACAATAA ACAAATACAA ATAAAAATAT AGTATTATCA
HapAA                  CATGAGACTT GACAAATCGA GATTCTTCTA TTCTATATAT CTAGAATATA GATAAGGTAT AATACAATAA ACAAATACAA ATAAAAATAT AGTATTATCA
HapH30                 CATGAGACTT GACAAATCGA GATTCTTCTA TTCTATATAT CTAGAATATA GA-AAGGTAT AATACAATAA ACAAATACAA ATAAAAATAT AGTATTATCA
HapI                   CATGAGACTT GACAAATCGA GATTCTTCTA TTCTATATAT CTAGAATATA GA-AAGGTAT AATACAATAA ACAAATACAA ATAAAAATAT AGTATTATCA
HapE                   CATGAGACTT GACAAATCGA GATTCTTCTA TTCTATATAT CTAGAATATA GATAAGGTAT AATACAATAA ACAAATACAA ATAAAAATAT AGTATTATCA
HapAC                  CATGAGACTT GACAAATCGA GATTCTTCTA TTCTATATAT CTAGAATATA GATAAGGTAT AATACAATAA ACAAATACAA ATAAAAATAT AGTATTATCA
HapF                   CATGAGACTT GACAAATCGA GATTCTTCTA TTCTATATAT CTAGAATATA GATAAGGTAT AATACAATAA ACAAATACAA ATAAAAATAT AGTATTATCA
HapH                   CATGAGACTT GACAAATCGA GATTCTTCTA TTCTATATAT CTAGAATATA GATAAGGTAT AATACAATAA ACAAATACAA ATAAAAATAT AGTATTATCA
HapG                   CATGAGACTT GACAAATCGA GATTCTTCTA TTCTATATAT CTAGAATATA GATAAGGTAT AATACAATAA ACAAATACAA ATAAAAATAT AGTATTATCA
HapAB                  CATGAGACTT GACAAATCGA GATTCTTCTA TTCTATATAT CTAGAATATA GATAAGGTAT AATACAATAA ACAAATACAA ATAAAAATAT AGTATTATCA
HapZ                   CATGAGACTT GACAAATCGA GATTCTTCTA TTCTATATAT CTAGAATATA GATAAGGTAT AATACAATAA ACAAATACAA ATAAAAATAT AGTATTATCA
HapD                   CATGAGACTT GACAAATCGA GATTCTTCTA TTCTATATAT CTAGAATATA GATAAGGTAT AATACAATAA ACAAATACAA ATAAAAATAT AGTATTATCA
HapA                   CATGAGACTT GACAAATCGA GATTCTTCTA TTCTATATAT CTAGAATATA GATAAGGTAT AATACAATAA ACAAATACAA ATAAAAATAT AGTATTATCA
HapB                   CATGAGACTT GACAAATCGA GATTCTTCTA TTCTATATAT CTAGAATATA GATAAGGTAT AATACAATAA ACAAATACAA ATAAAAATAT AGTATTATCA
HapC                   CATGAGACTT GACAAATCGA GATTCTTCTA TTCTATATAT CTAGAATATA GATAAGGTAT AATACAATAA ACAAATACAA ATAAAAATAT AGTATTATCA
HapY                   CATGAGACTT GACAAATCGA GATTCTTCTA TTCTATATAT CTAGAATATA GA-AAGGTAT AATACAATAA ACAAATACAA ATAAAAATAT AGTATTATCA
HapW                   CATGAGACTT GACAAATCGA GATTCTTCTA TTCTATATAT CTAGAATATA GA-AAGGTAT AATACAATAA ACAAATACAA ATAAAAATAT AGTATTATCA
HapX                   CATGAGACTT GACAAATCGA GATTCTTCTA TTCTATATAT CTAGAATATA GA-AAGGTAT AATACAATAA ACAAATACAA ATAAAAATAT AGTATTATCA
HapS                   CATGAGACTT GACAAATCGA GATTCTTCTA TTCTATATAT CTAGAATATA GATAAGGTAT AATACAATAA ACAAATACAA ATAAAAATAT AGTATTATCA
HaKonecNC_025235       CATGAGACTT GACAAATCGA GATTCTTCTA TTCTATATAT CTAGAATATA GA-AAGGTAT AATACAATAA ACAAATACAA ATAAAAATAT AGTATTATCA
Monparadoxtrnt         CATGAAACTT GACAAATCGA GATTCTTCTA TTCTATATAT CTAGAATATA GA-AAGGTAT AATACAATAA ACAAATATAA ATCAAAATAA AGTATTATCA

                       ....|....| ....|....| ....|....| ....|....| ....|....| ....|....| ....|....| ....|....| ....|....| ....|....|
                            1305       1315       1325       1335       1345       1355       1365       1375       1385       1395

HapM                   TACGATAATG GAATCAAATA CGCAGTATTT ACAGAAAAGA GTCTTCGTTT ATTAGGAAAG AATCAATATA CTTCTA---- ----ATGTCG AATTGGGATT
HapM1                  TACGATAATG GAATCAAATA CGCAGTATTT ACAGAAAAGA GTCTTCGTTT ATTAGGAAAG AATCAATATA CTTCTA---- ----ATGTCG AATTGGGATT
FU12.1                 TACGATAATG GAATCAAATA CGCAGTATTT ACAGAAAAGA GTCTTCGTTT ATTAGGAAAG AATCAATATA CTTCTA---- ----ATGTCG AATTGGGATT
FU10.1                 TACGATAATG GAATCAAATA CGCAGTATTT ACAGAAAAGA GTCTTCGTTT ATTAGGAAAG AATCAATATA CTTCTA---- ----ATGTCG AATTGGGATT
FU1.1                  TACGATAATG GAATCAAATA CGCAGTATTT ACAGAAAAGA GTCTTCGTTT ATTAGGAAAG AATCAATATA CTTCTA---- ----ATGTCG AATTGGGATT
FU2.1                  TACGATAATG GAATCAAATA CGCAGTATTT ACAGAAAAGA GTCTTCGTTT ATTAGGAAAG AATCAATATA CTTCTA---- ----ATGTCG AATTGGGATT
FU11.1                 TACGATAATG GAATCAAATA CGCAGTATTT ACAGAAAAGA GTCTTCGTTT ATTAGGAAAG AATCAATATA CTTCTA---- ----ATGTCG AATTGGGATT
CO4.1                  TACGATAATG GAATCAAATA CGCAGTATTT ACAGAAAAGA GTCTTCGTTT ATTAGGAAAG AATCAATATA CTTCTA---- ----ATGTCG AATTGGGATT
CO11.1                 TACGATAATG GAATCAAATA CGCAGTATTT ACAGAAAAGA GTCTTCGTTT ATTAGGAAAG AATCAATATA CTTCTA---- ----ATGTCG AATTGGGATT
TR1.1                  TACGATAATG GAATCAAATA CGCAGTATTT ACAGAAAAGA GTCTTCGTTT ATTAGGAAAG AATCAATATA CTTCTA---- ----ATGTCG AATTGGGATT
TR3.1                  TACGATAATG GAATCAAATA CGCAGTATTT ACAGAAAAGA GTCTTCGTTT ATTAGGAAAG AATCAATATA CTTCTA---- ----ATGTCG AATTGGGATT
TR10.1                 TACGATAATG GAATCAAATA CGCAGTATTT ACAGAAAAGA GTCTTCGTTT ATTAGGAAAG AATCAATATA CTTCTA---- ----ATGTCG AATTGGGATT
TR11.1                 TACGATAATG GAATCAAATA CGCAGTATTT ACAGAAAAGA GTCTTCGTTT ATTAGGAAAG AATCAATATA CTTCTA---- ----ATGTCG AATTGGGATT
Vi16.1                 TACGATAATG GAATCAAATA CGCAGTATTT ACAGAAAAGA GTCTTCGTTT ATTAGGAAAG AATCAATATA CTTCTA---- ----ATGTCG AATTGGGATT
VI1.1                  TACGATAATG GAATCAAATA CGCAGTATTT ACAGAAAAGA GTCTTCGTTT ATTAGGAAAG AATCAATATA CTTCTA---- ----ATGTCG AATTGGGATT
Vi3.1                  TACGATAATG GAATCAAATA CGCAGTATTT ACAGAAAAGA GTCTTCGTTT ATTAGGAAAG AATCAATATA CTTCTA---- ----ATGTCG AATTGGGATT
CH1.1                  TACGATAATG GAATCAAATA CGCAGTATTT ACAGAAAAGA GTCTTCGTTT ATTAGGAAAG AATCAATATA CTTCTA---- ----ATGTCG AATTGGGATT
CH4.1                  TACGATAATG GAATCAAATA CGCAGTATTT ACAGAAAAGA GTCTTCGTTT ATTAGGAAAG AATCAATATA CTTCTA---- ----ATGTCG AATTGGGATT
CH6.1                  TACGATAATG GAATCAAATA CGCAGTATTT ACAGAAAAGA GTCTTCGTTT ATTAGGAAAG AATCAATATA CTTCTA---- ----ATGTCG AATTGGGATT
CH2.2                  TACGATAATG GAATCAAATA CGCAGTATTT ACAGAAAAGA GTCTTCGTTT ATTAGGAAAG AATCAATATA CTTCTA---- ----ATGTCG AATTGGGATT
CH2.1                  TACGATAATG GAATCAAATA CGCAGTATTT ACAGAAAAGA GTCTTCGTTT ATTAGGAAAG AATCAATATA CTTCTA---- ----ATGTCG AATTGGGATT
HapAD                  TACGATAATG GAATCAAATA CGCAGTATTT ACAGAAAAGA GTCTTCGTTT ATTAGGAAAG AATCAATATA CTTCTA---- ----ATGTCG AATTGGGATT
HapO                   TACGATAATG GAATCAAATA CGCAGTATTT ACAGAAAAGA GTCTTCGTTT ATTAGGAAAG AATCAATATA CTTCTA---- ----ATGTCG AATTGGGATT
HapN                   TACGATAATG GAATCAAATA CGCAGTATTT ACAGAAAAGA GTCTTCGTTT ATTAGGAAAG AATCAATATA CTTCTA---- ----ATGTCG AATTGGGATT
HapK                   TACGATAATG GAATCAAATA CGCAGTATTT ACAGAAAAGA GTCTTCGTTT ATTAGGAAAG AATCAATATA CTTCTA---- ----ATGTCG AATTGGGATT
HapJ                   TACGATAATG GAATCAAATA CGCAGTATTT ACAGAAAAGA GTCTTCGTTT ATTAGGAAAG AATCAATATA CTTCTA---- ----ATGTCG AATTGGGATT
HapL                   TACGATAATG GAATCAAATA CGCAGTATTT ACAGAAAAGA GTCTTCGTTT ATTAGGAAAG AATCAATATA CTTCTA---- ----ATGTCG AATTGGGATT
HapQ                   TACGATAATG GAATCAAATA CGCAGTATTT ACAGAAAAGA GTCTTCGTTT ATTAGGAAAG AATCAATATA CTTCTA---- ----ATGTCG AATTGGGATT
HapV                   TACGATAATG GAATCAAATA CGCAGTATTT ACAGAAAAGA GTCTTCGTTT ATTAGGAAAG AATCAATATA CTTCTA---- ----ATGTCG AATTGGGATT
HapR                   TACGATAATG GAATCAAATA CGCAGTATTT ACAGAAAAGA GTCTTCGTTT ATTAGGAAAG AATCAATATA CTTCTA---- ----ATGTCG AATTGGGATT
HapP                   TACGATAATG GAATCAAATA CGCAGTATTT ACAGAAAAGA GTCTTCGTTT ATTAGGAAAG AATCAATATA CTTCTA---- ----ATGTCG AATTGGGATT
HapU                   TACGATAATG GAATCAAATA CGCAGTATTT ACAGAAAAGA GTCTTCGTTT ATTAGGAAAG AATCAATATA CTTCTA---- ----ATGTCG AATTGGGATT
HapT                   TACGATAATG GAATCAAATA CGCAGTATTT ACAGAAAAGA GTCTTCGTTT ATTAGGAAAG AATCAATATA CTTCTA---- ----ATGTCG AATTGGGATT
HapAA                  TACGATAATG ATATCTAGAA TATAGATAAG GTATAATACA ATAAACAAAT ACAAATAAAA ATATAGTATT ATCATACGAT AATGNNNNNN NNNNNNNNNN
HapH30                 TACGATAATG GAATCAAATA CGCAGTATTT ACAGAAAAGA GTCTTCGTTT ATTAGGAAAG AATCAATATA CTTCTA---- ----ATGTCG AATTGGGATT
HapI                   TACGATAATG GAATCAAATA CGCAGTATTT ACAGAAAAGA GTCTTCGTTT ATTAGGAAAG AATCAATATA CTTCTA---- ----ATGTCG AATTGGGATT
HapE                   TACGATAATG GAATCAAATA CGCAGTATTT ACAGAAAAGA GTCTTCGTTT ATTAGGAAAG AATCAATATA CTTCTA---- ----ATGTCG AATTGGGATT
HapAC                  TACGATAATG GAATCAAATA CGCAGTATTT ACAGAAAAGA GTCTTCGTTT ATTAGGAAAG AATCAATATA CTTCTA---- ----ATGTCG AATTGGGATT
HapF                   TACGATAATG GAATCAAATA CGCAGTATTT ACAGAAAAGA GTCTTCGTTT ATTAGGAAAG AATCAATATA CTTCTA---- ----ATGTCG AATTGGGATT
HapH                   TACGATAATG GAATCAAATA CGCAGTATTT ACAGAAAAGA GTCTTCGTTT ATTAGGAAAG AATCAATATA CTTCTA---- ----ATGTCG AATTGGGATT
HapG                   TACGATAATG GAATCAAATA CGCAGTATTT ACAGAAAAGA GTCTTCGTTT ATTAGGAAAG AATCAATATA CTTCTA---- ----ATGTCG AATTGGGATT
HapAB                  TACGATAATG GAATCAAATA CGCAGTATTT ACAGAAAAGA GTCTTCGTTT ATTAGGAAAG AATCAATATA CTTCTA---- ----ATGTCG AATTGGGATT
HapZ                   TACGATAATG GAATCAAATA CGCAGTATTT ACAGAAAAGA GTCTTCGTTT ATTAGGAAAG AATCAATATA CTTCTA---- ----ATGTCG AATTGGGATT
HapD                   TACGATAATG GAATCAAATA CGCAGTATTT ACAGAAAAGA GTCTTCGTTT ATTAGGAAAG AATCAATATA CTTCTA---- ----ATGTCG AATTGGGATT
HapA                   TACGATAATG GAATCAAATA CGCAGTATTT ACAGAAAAGA GTCTTCGTTT ATTAGGAAAG AATCAATATA CTTCTA---- ----ATGTCG AATTGGGATT
HapB                   TACGATAATG GAATCAAATA CGCAGTATTT ACAGAAAAGA GTCTTCGTTT ATTAGGAAAG AATCAATATA CTTCTA---- ----ATGTCG AATTGGGATT
HapC                   TACGATAATG GAATCAAATA CGCAGTATTT ACAGAAAAGA GTCTTCGTTT ATTAGGAAAG AATCAATATA CTTCTA---- ----ATGTCG AATTGGGATT
HapY                   TACGATAATG GAATCAAATA CGCAGTATTT ACAGAAAAGA GTCTTCGTTT ATTAGGAAAG AATCAATATA CTTCTA---- ----ATGTCG AATTGGGATT
HapW                   TACGATAATG GAATCAAATA CGCAGTATTT ACAGAAAAGA GTCTTCGTTT ATTAGGAAAG AATCAATATA CTTCTA---- ----ATGTCG AATTGGGATT
HapX                   TACGATAATG GAATCAAATA CGCAGTATTT ACAGAAAAGA GTCTTCGTTT ATTAGGAAAG AATCAATATA CTTCTA---- ----ATGTCG AATTGGGATT
HapS                   TACGATAATG GAATCAAATA CGCAGTATTT ACAGAAAAGA GTCTTCGTTT ATTAGGAAAG AATCAATATA CTTCTA---- ----ATGTCG AATTGGGATT
HaKonecNC_025235       TACGATAATG GAATCAAATA TGCAGTATTT ACAGAAAAGA GTCTTCGTTT ATTAGGAAAG AATCAATATA CTTCTA---- ----ATGTCG AATCGGGATT
Monparadoxtrnt         TACGATAATG GAATCAAATA CGCAGTATTT ACAGAAAAGA GTCTTCATTT ATTGGGAAAG AATCAATATA CTTTTA---- ----ATGTCG AATCGGGATT

                       ....|....| ....|....| ....|....| ....|....| ....|....| ....|....| ....|....| ....|....| ....|....| ....|....|
                            1405       1415       1425       1435       1445       1455       1465       1475       1485       1495

HapM                   CACTAAGGCA GAAATAAAGC ATTGGGTCGA ACTCTTCTTT GGTGTTAAGG TAGTAGCTGT GAATAGCCAT CGACTACCCG GAAAGGGTAG AAGAACGGGA
HapM1                  CACTAAGGCA GAAATAAAGC ATTGGGTCGA ACTCTTCTTT GGTGTTAAGG TAGTAGCTGT GAATAGCCAT CGACTACCCG GAAAGGGTAG AAGAACGGGA
FU12.1                 CACTAAGGCA GAAATAAAGC ATTGGGTCGA ACTCTTCTTT GGTGTTAAGG TAGTAGCTGT GAATAGCCAT CGACTACCCG GAAAGGGTAG AAGAACGGGA
FU10.1                 CACTAAGGCA GAAATAAAGC ATTGGGTCGA ACTCTTCTTT GGTGTTAAGG TAGTAGCTGT GAATAGCCAT CGACTACCCG GAAAGGGTAG AAGAACGGGA
FU1.1                  CACTAAGGCA GAAATAAAGC ATTGGGTCGA ACTCTTCTTT GGTGTTAAGG TAGTAGCTGT GAATAGCCAT CGACTACCCG GAAAGGGTAG AAGAACGGGA
FU2.1                  CACTAAGGCA GAAATAAAGC ATTGGGTCGA ACTCTTCTTT GGTGTTAAGG TAGTAGCTGT GAATAGCCAT CGACTACCCG GAAAGGGTAG AAGAACGGGA
FU11.1                 CACTAAGGCA GAAATAAAGC ATTGGGTCGA ACTCTTCTTT GGTGTTAAGG TAGTAGCTGT GAATAGCCAT CGACTACCCG GAAAGGGTAG AAGAACGGGA
CO4.1                  CACTAAGGCA GAAATAAAGC ATTGGGTCGA ACTCTTCTTT GGTGTTAAGG TAGTAGCTGT GAATAGCCAT CGACTACCCG GAAAGGGTAG AAGAACGGGA
CO11.1                 CACTAAGGCA GAAATAAAGC ATTGGGTCGA ACTCTTCTTT GGTGTTAAGG TAGTAGCTGT GAATAGCCAT CGACTACCCG GAAAGGGTAG AAGAACGGGA
TR1.1                  CACTAAGGCA GAAATAAAGC ATTGGGTCGA ACTCTTCTTT GGTGTTAAGG TAGTAGCTGT GAATAGCCAT CGACTACCCG GAAAGGGTAG AAGAACGGGA
TR3.1                  CACTAAGGCA GAAATAAAGC ATTGGGTCGA ACTCTTCTTT GGTGTTAAGG TAGTAGCTGT GAATAGCCAT CGACTACCCG GAAAGGGTAG AAGAACGGGA
TR10.1                 CACTAAGGCA GAAATAAAGC ATTGGGTCGA ACTCTTCTTT GGTGTTAAGG TAGTAGCTGT GAATAGCCAT CGACTACCCG GAAAGGGTAG AAGAACGGGA
TR11.1                 CACTAAGGCA GAAATAAAGC ATTGGGTCGA ACTCTTCTTT GGTGTTAAGG TAGTAGCTGT GAATAGCCAT CGACTACCCG GAAAGGGTAG AAGAACGGGA
Vi16.1                 CACTAAGGCA GAAATAAAGC ATTGGGTCGA ACTCTTCTTT GGTGTTAAGG TAGTAGCTGT GAATAGCCAT CGACTACCCG GAAAGGGTAG AAGAACGGGA
VI1.1                  CACTAAGGCA GAAATAAAGC ATTGGGTC-A CCTCTTCTTT GGTGTTAAGG TAGTAGCTGT GAATAGCCAT CGACTACCCG GAAAGGGTAG AAGAACGGGA
Vi3.1                  CACTAAGGCA GAAATAAAGC ATTGGGTC-A CCTCTTCTTT GGTGTTAAGG TAGTAGCTGT GAATAGCCAT CGACTACCCG GAAAGGGTAG AAGAACGGGA
CH1.1                  CACTAAGGCA GAAATAAAGC ATTGGGTCGA ACTCTTCTTT GGTGTTAAGG TAGTAGCTGT GAATAGCCAT CGACTACCCG GAAAGGGTAG AAGAACGGGA
CH4.1                  CACTAAGGCA GAAATAAAGC ATTGGGTCGA ACTCTTCTTT GGTGTTAAGG TAGTAGCTGT GAATAGCCAT CGACTACCCG GAAAGGGTAG AAGAACGGGA
CH6.1                  CACTAAGGCA GAAATAAAGC ATTGGGTCGA ACTCTTCTTT GGTGTTAAGG TAGTAGCTGT GAATAGCCAT CGACTACCCG GAAAGGGTAG AAGAACGGGA
CH2.2                  CACTAAGGCA GAAATAAAGC ATTGGGTCGA ACTCTTCTTT GGTGTTAAGG TAGTAGCTGT GAATAGCCAT CGACTACCCG GAAAGGGTAG AAGAACGGGA
CH2.1                  CACTAAGGCA GAAATAAAGC ATTGGGTCGA ACTCTTCTTT GGTGTTAAGG TAGTAGCTGT GAATAGCCAT CGACTACCCG GAAAGGGTAG AAGAACGGGA
HapAD                  CACTAAGGCA GAAATAAAGC ATTGGGTCGA ACTCTTCTTT GGTGTTAAGG TAGTAGCTGT GAATAGCCAT CGACTACCCG GAAAGGGTAG AAGAACGGGA
HapO                   CACTAAGGCA GAAATAAAGC ATTGGGTCGA ACTCTTCTTT GGTGTTAAGG TAGTAGCTGT GAATAGCCAT CGACTACCCG GAAAGGGTAG AAGAACGGGA
HapN                   CACTAAGGCA GAAATAAAGC ATTGGGTCGA ACTCTTCTTT GGTGTTAAGG TAGTAGCTGT GAATAGCCAT CGACTACCCG GAAAGGGTAG AAGAACGGGA
HapK                   CACTAAGGCA GAAATAAAGC ATTGGGTCGA ACTCTTCTTT GGTGTTAAGG TAGTAGCTGT GAATAGCCAT CGACTACCCG GAAAGGGTAG AAGAACGGGA
HapJ                   CACTAAGGCA GAAATAAAGC ATTGGGTCGA ACTCTTCTTT GGTGTTAAGG TAGTAGCTGT GAATAGCCAT CGACTACCCG GAAAGGGTAG AAGAACGGGA
HapL                   CACTAAGGCA GAAATAAAGC ATTGGGTCGA ACTCTTCTTT GGTGTTAAGG TAGTAGCTGT GAATAGCCAT CGACTACCCG GAAAGGGTAG AAGAACGGGA
HapQ                   CACTAAGGCA GAAATAAAGC ATTGGGTCGA ACTCTTCTTT GGTGTTAAGG TAGTAGCTGT GAATAGCCAT CGACTACCCG GAAAGGGTAG AAGAACGGGA
HapV                   CACTAAGGCA GAAATAAAGC ATTGGGTCGA ACTCTTCTTT GGTGTTAAGG TAGTAGCTGT GAATAGCCAT CGACTACCCG GAAAGGGTAG AAGAACGGGA
HapR                   CACTAAGGCA GAAATAAAGC ATTGGGTCGA ACTCTTCTTT GGTGTTAAGG TAGTAGCTGT GAATAGCCAT CGACTACCCG GAAAGGGTAG AAGAACGGGA
HapP                   CACTAAGGCA GAAATAAAGC ATTGGGTCGA ACTCTTCTTT GGTGTTAAGG TAGTAGCTGT GAATAGCCAT CGACTACCCG GAAAGGGTAG AAGAACGGGA
HapU                   CACTAAGGCA GAAATAAAGC ATTGGGTCGA ACTCTTCTTT GGTGTTAAGG TAGTAGCTGT GAATAGCCAT CGACTACCCG GAAAGGGTAG AAGAACGGGA
HapT                   CACTAAGGCA GAAATAAAGC ATTGGGTCGA ACTCTTCTTT GGTGTTAAGG TAGTAGCTGT GAATAGCCAT CGACTACCCG GAAAGGGTAG AAGAACGGGA
HapAA                  NNNNNNNNNN NNNNNNNNNN NNNNNNNNNN NNNNNNNNNN NNNNNNNNNN NNNNNNNNNN NNNNNNNNNN NNNNNNNNNN NNNNNNNNNN NNNNNNNNNN
HapH30                 CACTAAGGCA GAAATAAAGC ATTGGGTCGA ACTCTTCTTT GGTGTTAAGG TAGTAGCTGT GAATAGCCAT CGACTACCCG GAAAGGGTAG AAGAACGGGA
HapI                   CACTAAGGCA GAAATAAAGC ATTGGGTCGA ACTCTTCTTT GGTGTTAAGG TAGTAGCTGT GAATAGCCAT CGACTACCCG GAAAGGGTAG AAGAACGGGA
HapE                   CACTAAGGCA GAAATAAAGC ATTGGGTCGA ACTCTTCTTT GGTGTTAAGG TAGTAGCTGT GAATAGCCAT CGACTACCCG GAAAGGGTAG AAGAACGGGA
HapAC                  CACTAAGGCA GAAATAAAGC ATTGGGTCGA ACTCTTCTTT GGTGTTAAGG TAGTAGCTGT GAATAGCCAT CGACTACCCG GAAAGGGTAG AAGAACGGGA
HapF                   CACTAAGGCA GAAATAAAGC ATTGGGTCGA ACTCTTCTTT GGTGTTAAGG TAGTAGCTGT GAATAGCCAT CGACTACCCG GAAAGGGTAG AAGAACGGGA
HapH                   CACTAAGGCA GAAATAAAGC ATTGGGTCGA ACTCTTCTTT GGTGTTAAGG TAGTAGCTGT GAATAGCCAT CGACTACCCG GAAAGGGTAG AAGAACGGGA
HapG                   CACTAAGGCA GAAATAAAGC ATTGGGTCGA ACTCTTCTTT GGTGTTAAGG TAGTAGCTGT GAATAGCCAT CGACTACCCG GAAAGGGTAG AAGAACGGGA
HapAB                  CACTAAGGCA GAAATAAAGC ATTGGGTCGA ACTCTTCTTT GGTGTTAAGG TAGTAGCTGT GAATAGCCAT CGACTACCCG GAAAGGGTAG AAGAACGGGA
HapZ                   CACTAAGGCA GAAATAAAGC ATTGGGTCGA ACTCTTCTTT GGTGTTAAGG TAGTAGCTGT GAATAGCCAT CGACTACCCG GAAAGGGTAG AAGAACGGGA
HapD                   CACTAAGGCA GAAATAAAGC ATTGGGTCGA ACTCTTCTTT GGTGTTAAGG TAGTAGCTGT GAATAGCCAT CGACTACCCG GAAAGGGTAG AAGAACGGGA
HapA                   CACTAAGGCA GAAATAAAGC ATTGGGTCGA ACTCTTCTTT GGTGTTAAGG TAGTAGCTGT GAATAGCCAT CGACTACCCG GAAAGGGTAG AAGAACGGGA
HapB                   CACTAAGGCA GAAATAAAGC ATTGGGTCGA ACTCTTCTTT GGTGTTAAGG TAGTAGCTGT GAATAGCCAT CGACTACCCG GAAAGGGTAG AAGAACGGGA
HapC                   CACTAAGGCA GAAATAAAGC ATTGGGTCGA ACTCTTCTTT GGTGTTAAGG TAGTAGCTGT GAATAGCCAT CGACTACCCG GAAAGGGTAG AAGAACGGGA
HapY                   CACTAAGGCA GAAATAAAGC ATTGGGTCGA ACTCTTCTTT GGTGTTAAGG TAGTAGCTGT GAATAGCCAT CGACTACCCG GAAAGGGTAG AAGAACGGGA
HapW                   CACTAAGGCA GAAATAAAGC ATTGGGTCGA ACTCTTCTTT GGTGTTAAGG TAGTAGCTGT GAATAGCCAT CGACTACCCG GAAAGGGTAG AAGAACGGGA
HapX                   CACTAAGGCA GAAATAAAGC ATTGGGTCGA ACTCTTCTTT GGTGTTAAGG TAGTAGCTGT GAATAGCCAT CGACTACCCG GAAAGGGTAG AAGAACGGGA
HapS                   CACTAAGGCA GAAATAAAGC ATTGGGTCGA ACTCTTCTTT GGTGTTAAGG TAGTAGCTGT GAATAGCCAT CGACTACCCG GAAAGGGTAG AAGAACGGGA
HaKonecNC_025235       CACTAAGACA GAAATAAAGC ATTGGGTCGA ACTCTTCTTT GGTGTTAAGG TAGTAGCTGT GAATAGCCAT CGACTACCCG GAAAGGGTAG AAGAACGGGA
Monparadoxtrnt         CACTAAGACA GAAATAAAGC ATTAGGTCGA ACTCTTTTTT GGTGTTAAGG TAGTAGCTGT GAATAGCCAT CGACTACCCG GAAAGGGTAG AAGAATGGGA
```

```
                    ....|....| ....|....| ....|....| ....|....| ....|....| ....|....| ....|....| ....|....| ....|....| ....|....|
                         1505       1515       1525       1535       1545       1555       1565       1575       1585       1595

HapM                CCTATTCTGG GACATACAAT GCATTACAGA CG--TACGAT CATTACCCTT CAACCGGGTA TTCTATTCCA CTTCTACTTT TTAATTAAAT TAAAGGGTAT
HapM1               CCTATTCTGG GACATACAAT GCATTACAGA CG--TACGAT CATTACCCTT CAACCGGGTA TTCTATTCCA CTTCTACTTT TTAATTAAAT TAAAGGGTAT
FU12.1              CCTATTCTGG GACATACAAT GCATTACAGA CG--TACGAT CATTACCCTT CAACCGGGTA TTCTATTCCA CTTCTACTTT TTAATTAAAT TAAAGGGTAT
FU10.1              CCTATTCTGG GACATACAAT GCATTACAGA CG--TACGAT CATTACCCTT CAACCGGGTA TTCTATTCCA CTTCTACTTT TTAATTAAAT TAAAGGGTAT
FU1.1               CCTATTCTGG GACATACAAT GCATTACAGA CG--TACGAT CATTACCCTT CAACCGGGTA TTCTATTCCA CTTCTACTTT TTAATTAAAT TAAAGGGTAT
FU2.1               CCTATTCTGG GACATACAAT GCATTACAGA CG--TACGAT CATTACCCTT CAACCGGGTA TTCTATTCCA CTTCTACTTT TTAATTAAAT TAAAGGGTAT
FU11.1              CCTATTCTGG GACATACAAT GCATTACAGA CG--TACGAT CATTACCCTT CAACCGGGTA TTCTATTCCA CTTCTACTTT TTAATTAAAT TAAAGGGTAT
CO4.1               CCTATTCTGG GACATACAAT GCATTACAGA CG--TACGAT CATTACCCTT CAACCGGGTA TTCTATTCCA CTTCTACTTT TTAATTAAAT TAAAGGGTAT
CO11.1              CCTATTCTGG GACATACAAT GCATTACAGA CG--TACGAT CATTACCCTT CAACCGGGTA TTCTATTCCA CTTCTACTTT TTAATTAAAT TAAAGGGTAT
TR1.1               CCTATTCTGG GACATACAAT GCATTACAGA CG--TACGAT CATTACCCTT CAACCGGGTA TTCTATTCCA CTTCTACTTT TTAATTAAAT TAAAGGGTAT
TR3.1               CCTATTCTGG GACATACAAT GCATTACAGA CG--TACGAT CATTACCCTT CAACCGGGTA TTCTATTCCA CTTCTACTTT TTAATTAAAT TAAAGGGTAT
TR10.1              CCTATTCTGG GACATACAAT GCATTACAGA CG--TACGAT CATTACCCTT CAACCGGGTA TTCTATTCCA CTTCTACTTT TTAATTAAAT TAAAGGGTAT
TR11.1              CCTATTCTGG GACATACAAT GCATTACAGA CG--TACGAT CATTACCCTT CAACCGGGTA TTCTATTCCA CTTCTACTTT TTAATTAAAT TAAAGGGTAT
Vi16.1              CCTATTCTGG GACATACAAT GCATTACAGA CGGTACGGAT CATTACCCTT CAACCGGGTA TTCTATTCCA CTTCTACTTT TTAATTAAAT TAAAGGGTAT
VI1.1               CCTATTCTGG GACATACAAT GCATTACAGA CGGTACGGAT CATTACCCTT CAACCGGGTA TTCTATTCCA CTTCTACTTT TTAATTAAAT TAAAGGGTAT
Vi3.1               CCTATTCTGG GACATACAAT GCATTACAGA CGGTACGGAT CATTACCCTT CAACCGGGTA TTCTATTCCA CTTCTACTTT TTAATTAAAT TAAAGGGTAT
CH1.1               CCTATTCTGG GACATACAAT GCATTACAGA CG--TACGAT CATTACCCTT CAACCGGGTA TTCTATTCCA CTTCTACTTT TTAATTAAAT TAAAGGGTAT
CH4.1               CCTATTCTGG GACATACAAT GCATTACAGA CG--TACGAT CATTACCCTT CAACCGGGTA TTCTATTCCA CTTCTACTTT TTAATTAAAT TAAAGGGTAT
CH6.1               CCTATTCTGG GACATACAAT GCATTACAGA CG--TACGAT CATTACCCTT CAACCGGGTA TTCTATTCCA CTTCTACTTT TTAATTAAAT TAAAGGGTAT
CH2.2               CCTATTCTGG GACATACAAT GCATTACAGA CG--TACGAT CATTACCCTT CAACCGGGTA TTCTATTCCA CTTCTACTTT TTAATTAAAT TAAAGGGTAT
CH2.1               CCTATTCTGG GACATACAAT GCATTACAGA CG--TACGAT CATTACCCTT CAACCGGGTA TTCTATTCCA CTTCTACTTT TTAATTAAAT TAAAGGGTAT
HapAD               CCTATTCTGG GACATACAAT GCATTACAGA CG--TACGAT CATTACCCTT CAACCGGGTA TTCTATTCCA CTTCTACTTT TTAATTAAAT TAAAGGGTAT
HapO                CCTATTCTGG GACATACAAT GCATTACAGA CG--TACGAT CATTACCCTT CAACCGGGTA TTCTATTCCA CTTCTACTTT TTAATTAAAT TAAAGGGTAT
HapN                CCTATTCTGG GACATACAAT GCATTACAGA CG--TACGAT CATTACCCTT CAACCGGGTA TTCTATTCCA CTTCTACTTT TTAATTAAAT TAAAGGGTAT
HapK                CCTATTCTGG GACATACAAT GCATTACAGA CG--TACGAT CATTACCCTT CAACCGGGTA TTCTATTCCA CTTCTACTTT TTAATTAAAT TAAAGGGTAT
HapJ                CCTATTCTGG GACATACAAT GCATTACAGA CG--TACGAT CATTACCCTT CAACCGGGTA TTCTATTCCA CTTCTACTTT TTAATTAAAT TAAAGGGTAT
HapL                CCTATTCTGG GACATACAAT GCATTACAGA CG--TACGAT CATTACCCTT CAACCGGGTA TTCTATTCCA CTTCTACTTT TTAATTAAAT TAAAGGGTAT
HapQ                CCTATTCTGG GACATACAAT GCATTACAGA CG--TACGAT CATTACCCTT CAACCGGGTA TTCTATTCCA CTTCTACTTT TTAATTAAAT TAAAGGGTAT
HapV                CCTATTCTGG GACATACAAT GCATTACAGA CG--TACGAT CATTACCCTT CAACCGGGTA TTCTATTCCA CTTCTACTTT TTAATTAAAT TAAAGGGTAT
HapR                CCTATTCTGG GACATACAAT GCATTACAGA CG--TACGAT CATTACCCTT CAACCGGGTA TTCTATTCCA CTTCTACTTT TTAATTAAAT TAAAGGGTAT
HapP                CCTATTCTGG GACATACAAT GCATTACAGA CG--TACGAT CATTACCCTT CAACCGGGTA TTCTATTCCA CTTCTACTTT TTAATTAAAT TAAAGGGTAT
HapU                CCTATTCTGG GACATACAAT GCATTACAGA CG--TACGAT CATTACCCTT CAACCGGGTA TTCTATTCCA CTTCTACTTT TTAATTAAAT TAAAGGGTAT
HapT                CCTATTCTGG GACATACAAT GCATTACAGA CG--TACGAT CATTACCCTT CAACCGGGTA TTCTATTCCA CTTCTACTTT TTAATTAAAT TAAAGGGTAT
HapAA               NNNNNNNNNN NNNNNNNNNN NNNNNNNNNN NN--NNNNNN NNNNNNNNNN NNNNNNNNNN NNNNNNNNNN NNNNNNNNNN NNNNNNNNNN NNNNNNNNNN
HapH30              CCTATTCTGG GACATACAAT GCATTACAGA CG--TACGAT CATTACCCTT CAACCGGGTA TTCTATTCCA CTTCTACTTT TTAATTAAAT TAAAGGGTAT
HapI                CCTATTCTGG GACATACAAT GCATTACAGA CG--TACGAT CATTACCCTT CAACCGGGTA TTCTATTCCA CTTCTACTTT TTAATTAAAT TAAAGGGTAT
HapE                CCTATTCTGG GACATACAAT GCATTACAGA CG--TACGAT CATTACCCTT CAACCGGGTA TTCTATTCCA CTTCTACTTT TTAATTAAAT TAAAGGGTAT
HapAC               CCTATTCTGG GACATACAAT GCATTACAGA CG--TACGAT CATTACCCTT CAACCGGGTA TTCTATTCCA CTTCTACTTT TTAATTAAAT TAAAGGGTAT
HapF                CCTATTCTGG GACATACAAT GCATTACAGA CG--TACGAT CATTACCCTT CAACCGGGTA TTCTATTCCA CTTCTACTTT TTAATTAAAT TAAAGGGTAT
HapH                CCTATTCTGG GACATACAAT GCATTACAGA CG--TACGAT CATTACCCTT CAACCGGGTA TTCTATTCCA CTTCTACTTT TTAATTAAAT TAAAGGGTAT
HapG                CCTATTCTGG GACATACAAT GCATTACAGA CG--TACGAT CATTACCCTT CAACCGGGTA TTCTATTCCA CTTCTACTTT TTAATTAAAT TAAAGGGTAT
HapAB               CCTATTCTGG GACATACAAT GCATTACAGA CG--TACGAT CATTACCCTT CAACCGGGTA TTCTATTCCA CTTCTACTTT TTAATTAAAT TAAAGGGTAT
HapZ                CCTATTCTGG GACATACAAT GCATTACAGA CG--TACGAT CATTACCCTT CAACCGGGTA TTCTATTCCA CTTCTACTTT TTAATTAAAT TAAAGGGTAT
HapD                CCTATTCTGG GACATACAAT GCATTACAGA CG--TACGAT CATTACCCTT CAACCGGGTA TTCTATTCCA CTTCTACTTT TTAATTAAAT TAAAGGGTAT
HapA                CCTATTCTGG GACATACAAT GCATTACAGA CG--TACGAT CATTACCCTT CAACCGGGTA TTCTATTCCA CTTCTACTTT TTAATTAAAT TAAAGGGTAT
HapB                CCTATTCTGG GACATACAAT GCATTACAGA CG--TACGAT CATTACCCTT CAACCGGGTA TTCTATTCCA CTTCTACTTT TTAATTAAAT TAAAGGGTAT
HapC                CCTATTCTGG GACATACAAT GCATTACAGA CG--TACGAT CATTACCCTT CAACCGGGTA TTCTATTCCA CTTCTACTTT TTAATTAAAT TAAAGGGTAT
HapY                CCTATTCTGG GACATACAAT GCATTACAGA CG--TACGAT CATTACCCTT CAACCGGGTA TTCTATTCCA CTTCTACTTT TTAATTAAAT TAAAGGGTAT
HapW                CCTATTCTGG GACATACAAT GCATTACAGA CG--TACGAT CATTACCCTT CAACCGGGTA TTCTATTCCA CTTCTACTTT TTAATTAAAT TAAAGGGTAT
HapX                CCTATTCTGG GACATACAAT GCATTACAGA CG--TACGAT CATTACCCTT CAACCGGGTA TTCTATTCCA CTTCTACTTT TTAATTAAAT TAAAGGGTAT
HapS                CCTATTCTGG GACATACAAT GCATTACAGA CG--TACGAT CATTACCCTT CAACCGGGTA TTCTATTCCA CTTCTACTTT TTAATTAAAT TAAAGGGTAT
HaKonecNC_025235    CCTATTCTGG GACATACAAT GCATTACAGA TG--TACGAT CATTACCCTT CAACCGGGTA TTCTATTCCA CTTCTACTTT TTAATTAAAT TAAAGGGTAT
Monparadoxtrnt      CCTATTCTGG GACATACAAT GCATTACAGA CG--TATGAT CATTACCCTT CAACCGGGTA TTCTATCCCA CTTCTACTTT TTA------T TAAAGGGTAT

                    ....|....| ....|....| ....|....| ....|....| ....|....| ....|....| ....|....| ....|....| ....|....| ....|....|
                         1605       1615       1625       1635       1645       1655       1665       1675       1685       1695

HapM                TCTGAAAAAG GTATTTCTTT CATTTTCACA ATTGCTTTCT TTTGAATCCA ATTTCAAGTT CGATTAGAAG GATAGAAAGG CCATGAGAAT GGGAAAAGAA
HapM1               TCTGAAAAAG GTATTTCTTT CATTTTCACA ATTGCTTTCT TTTGAATCCA ATTTCAAGTT CGATTAGAAG GATAGAAAGG CCATGAGAAT GGGAAAAGAA
FU12.1              TCTGAAAAAG GTATTTCTTT CATTTTCACA ATTGCTTTCT TTTGAATCCA ATTTCAAGTT CGATTAGAAG GATAGAAAGG CCATGAGAAT GGGAAAAGAA
FU10.1              TCTGAAAAAG GTATTTCTTT CATTTTCACA ATTGCTTTCT TTTGAATCCA ATTTCAAGTT CGATTAGAAG GATAGAAAGG CCATGAGAAT GGGAAAAGAA
FU1.1               TCTGAAAAAG GTATTTCTTT CATTTTCACA ATTGCTTTCT TTTGAATCCA ATTTCAAGTT CGATTAGAAG GATAGAAAGG CCATGAGAAT GGGAAAAGAA
FU2.1               TCTGAAAAAG GTATTTCTTT CATTTTCACA ATTGCTTTCT TTTGAATCCA ATTTCAAGTT CGATTAGAAG GATAGAAAGG CCATGAGAAT GGGAAAAGAA
FU11.1              TCTGAAAAAG GTATTTCTTT CATTTTCACA ATTGCTTTCT TTTGAATCCA ATTTCAAGTT CGATTAGAAG GATAGAAAGG CCATGAGAAT GGGAAAAGAA
CO4.1               TCTGAAAAAG GTATTTCTTT CATTTTCACA ATTGCTTTCT TTTGAATCCA ATTTCAAGTT CGATTAGAAG GATAGAAAGG CCATGAGAAT GGGAAAAGAA
CO11.1              TCTGAAAAAG GTATTTCTTT CATTTTCACA ATTGCTTTCT TTTGAATCCA ATTTCAAGTT CGATTAGAAG GATAGAAAGG CCATGAGAAT GGGAAAAGAA
TR1.1               TCTGAAAAAG GTATTTCTTT CATTTTCACA ATTGCTTTCT TTTGAATCCA ATTTCAAGTT CGATTAGAAG GATAGAAAGG CCATGAGAAT GGGAAAAGAA
TR3.1               TCTGAAAAAG GTATTTCTTT CATTTTCACA ATTGCTTTCT TTTGAATCCA ATTTCAAGTT CGATTAGAAG GATAGAAAGG CCATGAGAAT GGGAAAAGAA
TR10.1              TCTGAAAAAG GTATTTCTTT CATTTTCACA ATTGCTTTCT TTTGAATCCA ATTTCAAGTT CGATTAGAAG GATAGAAAGG CCATGAGAAT GGGAAAAGAA
TR11.1              TCTGAAAAAG GTATTTCTTT CATTTTCACA ATTGCTTTCT TTTGAATCCA ATTTCAAGTT CGATTAGAAG GATAGAAAGG CCATGAGAAT GGGAAAAGAA
Vi16.1              TCTGAAAAAG GTATTTCTTT CATTTTCACA ATTGCTTTCT TTTGAATCCA ATTTCAAGTT CGATTAGAAG GATAGAAAGG CCATGAGAAT GGGAAAAGAA
VI1.1               TCTGAAAAAG GTATTTCTTT CATTTTCACA ATTGCTTTCT TTTGAATCCA ATTTCAAGTT CGATTAGAAG GATAGAAAGG CCATGAGAAT GGGAAAAGAA
Vi3.1               TCTGAAAAAG GTATTTCTTT CATTTTCACA ATTGCTTTCT TTTGAATCCA ATTTCAAGTT CGATTAGAAG GATAGAAAGG CCATGAGAAT GGGAAAAGAA
CH1.1               TCTGAAAAAG GTATTTCTTT CATTTTCACA ATTGCTTTCT TTTGAATCCA ATTTCAAGTT CGATTAGAAG GATAGAAAGG CCATGAGAAT GGGAAAAGAA
CH4.1               TCTGAAAAAG GTATTTCTTT CATTTTCACA ATTGCTTTCT TTTGAATCCA ATTTCAAGTT CGATTAGAAG GATAGAAAGG CCATGAGAAT GGGAAAAGAA
CH6.1               TCTGAAAAAG GTATTTCTTT CATTTTCACA ATTGCTTTCT TTTGAATCCA ATTTCAAGTT CGATTAGAAG GATAGAAAGG CCATGAGAAT GGGAAAAGAA
CH2.2               TCTGAAAAAG GTATTTCTTT CATTTTCACA ATTGCTTTCT TTTGAATCCA ATTTCAAGTT CGATTAGAAG GATAGAAAGG CCATGAGAAT GGGAAAAGAA
CH2.1               TCTGAAAAAG GTATTTCTTT CATTTTCACA ATTGCTTTCT TTTGAATCCA ATTTCAAGTT CGATTAGAAG GATAGAAAGG CCATGAGAAT GGGAAAAGAA
HapAD               TCTGAAAAAG GTATTTCTTT CATTTTCACA ATTGCTTTCT TTTGAATCCA ATTTCAAGTT CGATTAGAAG GATAGAAAGG CCATGAGAAT GGGAAAAGAA
HapO                TCTGAAAAAG GTATTTCTTT CATTTTCACA ATTGCTTTCT TTTGAATCCA ATTTCAAGTT CGATTAGAAG GATAGAAAGG CCATGAGAAT GGGAAAAGAA
HapN                TCTGAAAAAG GTATTTCTTT CATTTTCACA ATTGCTTTCT TTTGAATCCA ATTTCAAGTT CGATTAGAAG GATAGAAAGG CCATGAGAAT GGGAAAAGAA
HapK                TCTGAAAAAG GTATTTCTTT CATTTTCACA ATTGCTTTCT TTTGAATCCA ATTTCAAGTT CGATTAGAAG GATAGAAAGG CCATGAGAAT GGGAAAAGAA
HapJ                TCTGAAAAAG GTATTTCTTT CATTTTCACA ATTGCTTTCT TTTGAATCCA ATTTCAAGTT CGATTAGAAG GATAGAAAGG CCATGAGAAT GGGAAAAGAA
HapL                TCTGAAAAAG GTATTTCTTT CATTTTCACA ATTGCTTTCT TTTGAATCCA ATTTCAAGTT CGATTAGAAG GATAGAAAGG CCATGAGAAT GGGAAAAGAA
HapQ                TCTGAAAAAG GTATTTCTTT CATTTTCACA ATTGCTTTCT TTTGAATCCA ATTTCAAGTT CGATTAGAAG GATAGAAAGG CCATGAGAAT GGGAAAAGAA
HapV                TCTGAAAAAG GTATTTCTTT CATTTTCACA ATTGCTTTCT TTTGAATCCA ATTTCAAGTT CGATTAGAAG GATAGAAAGG CCATGAGAAT GGGAAAAGAA
HapR                TCTGAAAAAG GTATTTCTTT CATTTTCACA ATTGCTTTCT TTTGAATCCA ATTTCAAGTT CGATTAGAAG GATAGAAAGG CCATGAGAAT GGGAAAAGAA
HapP                TCTGAAAAAG GTATTTCTTT CATTTTCACA ATTGCTTTCT TTTGAATCCA ATTTCAAGTT CGATTAGAAG GATAGAAAGG CCATGAGAAT GGGAAAAGAA
HapU                TCTGAAAAAG GTATTTCTTT CATTTTCACA ATTGCTTTCT TTTGAATCCA ATTTCAAGTT CGATTAGAAG GATAGAAAGG CCATGAGAAT GGGAAAAGAA
HapT                TCTGAAAAAG GTATTTCTTT CATTTTCACA ATTGCTTTCT TTTGAATCCA ATTTCAAGTT CGATTAGAAG GATAGAAAGG CCATGAGAAT GGGAAAAGAA
HapAA               NNNNNNNNNN NNNNNNNNNN NNNNNNNNNN NNNNNNNNNN NNNNNNNNNN NNNNNNNNNT TGATTAGAAG GATAGAAAGG CCATGAGAAT GGGAAAAGAA
HapH30              TCTGAAAAAG GTATTTCTTT CATTTTCACA ATTGCTTTCT TTTGAATCCA ATTTCAAGTT CGATTAGAAG GATAGAAAGG CCATGAGAAT GGGAAAAGAA
HapI                TCTGAAAAAG GTATTTCTTT CATTTTCACA ATTGCTTTCT TTTGAATCCA ATTTCAAGTT CGATTAGAAG GATAGAAAGG CCATGAGAAT GGGAAAAGAA
HapE                TCTGAAAAAG GTATTTCTTT CATTTTCACA ATTGCTTTCT TTTGAATCCA ATTTCAAGTT CGATTAGAAG GATAGAAAGG CCATGAGAAT GGGAAAAGAA
HapAC               TCTGAAAAAG GTATTTCTTT CATTTTCACA ATTGCTTTCT TTTGAATCCA ATTTCAAGTT CGATTAGAAG GATAGAAAGG CCATGAGAAT GGGAAAAGAA
HapF                TCTGAAAAAG GTATTTCTTT CATTTTCACA ATTGCTTTCT TTTGAATCCA ATTTCAAGTT CGATTAGAAG GATAGAAAGG CCATGAGAAT GGGAAAAGAA
HapH                TCTGAAAAAG GTATTTCTTT CATTTTCACA ATTGCTTTCT TTTGAATCCA ATTTCAAGTT CGATTAGAAG GATAGAAAGG CCATGAGAAT GGGAAAAGAA
HapG                TCTGAAAAAG GTATTTCTTT CATTTTCACA ATTGCTTTCT TTTGAATCCA ATTTCAAGTT CGATTAGAAG GATAGAAAGG CCATGAGAAT GGGAAAAGAA
HapAB               TCTGAAAAAG GTATTTCTTT CATTTTCACA ATTGCTTTCT TTTGAATCCA ATTTCAAGTT CGATTAGAAG GATAGAAAGG CCATGAGAAT GGGAAAAGAA
HapZ                TCTGAAAAAG GTATTTCTTT CATTTTCACA ATTGCTTTCT TTTGAATCCA ATTTCAAGTT CGATTAGAAG GATAGAAAGG CCATGAGAAT GGGAAAAGAA
HapD                TCTGAAAAAG GTATTTCTTT CATTTTCACA ATTGCTTTCT TTTGAATCCA ATTTCAAGTT CGATTAGAAG GATAGAAAGG CCATGAGAAT GGGAAAAGAA
HapA                TCTGAAAAAG GTATTTCTTT CATTTTCACA ATTGCTTTCT TTTGAATCCA ATTTCAAGTT CGATTAGAAG GATAGAAAGG CCATGAGAAT GGGAAAAGAA
HapB                TCTGAAAAAG GTATTTCTTT CATTTTCACA ATTGCTTTCT TTTGAATCCA ATTTCAAGTT CGATTAGAAG GATAGAAAGG CCATGAGAAT GGGAAAAGAA
HapC                TCTGAAAAAG GTATTTCTTT CATTTTCACA ATTGCTTTCT TTTGAATCCA ATTTCAAGTT CGATTAGAAG GATAGAAAGG CCATGAGAAT GGGAAAAGAA
HapY                TCTGAAAAAG GTATTTCTTT CATTTTCACA ATTGCTTTCT TTTGAATCCA ATTTCAAGTT CGATTAGAAG GATAGAAAGG CCATGAGAAT GGGAAAAGAA
HapW                TCTGAAAAAG GTATTTCTTT CATTTTCACA ATTGCTTTCT TTTGAATCCA ATTTCAAGTT CGATTAGAAG GATAGAAAGG CCATGAGAAT GGGAAAAGAA
HapX                TCTGAAAAAG GTATTTCTTT CATTTTCACA ATTGCTTTCT TTTGAATCCA ATTTCAAGTT CGATTAGAAG GATAGAAAGG CCATGAGAAT GGGAAAAGAA
HapS                TCTGAAAAAG GTATTTCTTT CATTTTCACA ATTGCTTTCT TTTGAATCCA ATTTCAAGTT CGATTAGAAG GATAGAAAGG CCATGAGAAT GGGAAAAGAA
HaKonecNC_025235    TCTGAAAGAG GTATTTCTTT CATTTTCACA ATTGCTTTCT TTTGAATCCA ATTTCAAGTT CGATTAGAAG GATAGAAAGG CCATGAGAAT GGGAAAAGAA
Monparadoxtrnt      TCTGAAAGAA GGATTTCTTT CATTTTCACA ATTGCTTTCT TTTGAATCCA ATTTTAAGTT CGATTAGAAG GATAGAAAGG CCATGAGAAT GGGAAAAGAA

                    ....|....| ....|....| ....|....| ....|....| ....|....| ....|....| ....|....| ....|....| ....|....| ....|....|
                         1705       1715       1725       1735       1745       1755       1765       1775       1785       1795

HapM                AAATAAAATC TTTTTAATTA GTTCCCCTTT TTTGCAACTT TCTTATTATC CATTCCATTC ATTTTTTT-- ---------- ---------- ----------
HapM1               AAATAAAATC TTTTTAATTA GTTCCCCTTT TTTGCAACTT TCTTATTATC CATTCCATTC ATTTTTTT-- ---------- ---------- ----------
FU12.1              AAATAAAATC TTTTTAATTA GTTCCCCTTT TTTGCAACTT TCTTATTATC CATTCCATTC ATTTTTTT-- ---------- ---------- ----------
FU10.1              AAATAAAATC TTTTTAATTA GTTCCCCTTT TTTGCAACTT TCTTATTATC CATTCCATTC ATTTTTTT-- ---------- ---------- ----------
FU1.1               AAATAAAATC TTTTTAATTA GTTCCCCTTT TTTGCAACTT TCTTATTATC CATTCCATTC ATTTTTTT-- ---------- ---------- ----------
FU2.1               AAATAAAATC TTTTTAATTA GTTCCCCTTT TTTGCAACTT TCTTATTATC CATTCCATTC ATTTTTTT-- ---------- ---------- ----------
FU11.1              AAATAAAATC TTTTTAATTA GTTCCCCTTT TTTGCAACTT TCTTATTATC CATTCCATTC ATTTTTTT-- ---------- ---------- ----------
CO4.1               AAATAAAATC TTTTTAATTA GTTCCCCTTT TTTGCAACTT TCTTATTATC CATTCCATTC ATTTTTTT-- ---------- ---------- ----------
CO11.1              AAATAAAATC TTTTTAATTA GTTCCCCTTT TTTGCAACTT TCTTATTATC CATTCCATTC ATTTTTT--- ---------- ---------- ----------
TR1.1               AAATAAAATC TTTTTAATTA GTTCCCCTTT TTTGCAACTT TCTTATTATC CATTCCATTC ATTTTTTT-- ---------- ---------- ----------
TR3.1               AAATAAAATC TTTTTAATTA GTTCCCCTTT TTTGCAACTT TCTTATTATC CATTCCATTC ATTTTTTT-- ---------- ---------- ----------
TR10.1              AAATAAAATC TTTTTAATTA GTTCCCCTTT TTTGCAACTT TCTTATTATC CATTCCATTC ATTTTTTT-- ---------- ---------- ----------
TR11.1              AAATAAAATC TTTTTAATTA GTTCCCCTTT TTTGCAACTT TCTTATTATC CATTCCATTC ATTTTTTT-- ---------- ---------- ----------
Vi16.1              AAATAAAATC TTTTTAATTA GTTCCCCTTT TTTGCAACTT TCTTATTATC CATTCCATTC ATTTTTTT-- ---------- ---------- ----------
VI1.1               AAATAAAATC TTTTTAATTA GTTCCCCTTT TTTGCAACTT TCTTATTATC CATTCCATTC ATTTTTTT-- ---------- ---------- ----------
Vi3.1               AAATAAAATC TTTTTAATTA GTTCCCCTTT TTTGCAACTT TCTTATTATC CATTCCATTC ATTTTTTT-- ---------- ---------- ----------
CH1.1               AAATAAAATC TTTTTAATTA GTTCCCCTTT TTTGCAACTT TCTTATTATC CATTCCATTC ATTTTTT--- ---------- ---------- ----------
CH4.1               AAATAAAATC TTTTTAATTA GTTCCCCTTT TTTGCAACTT TCTTATTATC CATTCCATTC ATTTTTT--- ---------- ---------- ----------
CH6.1               AAATAAAATC TTTTTAATTA GTTCCCCTTT TTTGCAACTT TCTTATTATC CATTCCATTC ATTTTTT--- ---------- ---------- ----------
CH2.2               AAATAAAATC TTTTTAATTA GTTCCCCTTT TTTGCAACTT TCTTATTATC CATTCCATTC ATTTTTT--- ---------- ---------- ----------
CH2.1               AAATAAAATC TTTTTAATTA GTTCCCCTTT TTTGCAACTT TCTTATTATC CATTCCATTC ATTTTTT--- ---------- ---------- ----------
HapAD               AAATAAAATC TTTTTAATTA GTTCCCCTTT TTTGCAACTT TCTTATTATC CATTCCATTC ATTTTTTT-- ---------- ---------- ----------
HapO                AAATAAAATC TTTTTAATTA GTTCCCCTTT TTTGCAACTT TCTTATTATC CATTCCATTC ATTTTTTT-- ---------- ---------- ----------
HapN                AAATAAAATC TTTTTAATTA GTTCCCCTTT TTTGCAACTT TCTTATTATC CATTCCATTC ATTTTTTT-- ---------- ---------- ----------
HapK                AAATAAAATC TTTTTAATTA GTTCCCCTTT TTTGCAACTT TCTTATTATC CATTCCATTC ATTTTTTT-- ---------- ---------- ----------
HapJ                AAATAAAATC TTTTTAATTA GTTCCCCTTT TTTGCAACTT TCTTATTATC CATTCCATTC ATTTTTTT-- ---------- ---------- ----------
HapL                AAATAAAATC TTTTTAATTA GTTCCCCTTT TTTGCAACTT TCTTATTATC CATTCCATTC ATTTTTTT-- ---------- ---------- ----------
HapQ                AAATCAAATC TTTTTAATTA GTTCCCCTTT TTTGCAACTT TCTTATTATC CATTCCATTC ATTTTTTT-- ---------- ---------- ----------
HapV                AAATCAAATC TTTTTAATTA GTTCCCCTTT TTTGCAACTT TCTTATTATC CATTCCATTC ATTTTTTT-- ---------- ---------- ----------
HapR                AAATAAAATC TTTTTAATTA GTTCCCCTTT TTTGCAACTT TCTTATTATC CATTCCATTC ATTTTTTT-- ---------- ---------- ----------
HapP                AAATCAAATC TTTTTAATTA GTTCCCCTTT TTTGCAACTT TCTTATTATC CATTCCATTC ATTTTTTT-- ---------- ---------- ----------
HapU                AAATCAAATC TTTTTAATTA GTTCCCCTTT TTTGCAACTT TCTTATTATC CATTCCATTC ATTTTTTT-- ---------- ---------- ----------
HapT                AAATAAAATC TTTTTAATTA GTTCCCCTTT TTTGCAACTT TCTTATTATC CATTCCATTC ATTTTTTT-- ---------- ---------- ----------
HapAA               AAATCAAATC TTTTTAATTA GTTCCCCTTT TTTGCAACTT TCTTATTATC CATTCCATTC ATTTTTT--- ---------- ---------- ----------
HapH30              AAATCAAATC TTTTTAATTA GTTCCCCTTT TTTGCAACTT TCTTATTATC CATTCCATTC ATTTTTTT-- ---------- ---------- ----------
HapI                AAATCAAATC TTTTTAATTA GTTCCCCTTT TTTGCAACTT TCTTATTATC CATTCCATTC ATTTTTT--- ---------- ---------- ----------
HapE                AAATCAAATC TTTTTAATTA GTTCCCCTTT TTTGCAACTT TCTTATTATC CATTCCATTC ATTTTTT--- ---------- ---------- ----------
HapAC               AAATCAAATC TTTTTAATTA GTTCCCCTTT TTTGCAACTT TCTTATTATC CATTCCATTC ATTTTTT--- ---------- ---------- ----------
HapF                AAATCAAATC TTTTTAATTA GTTCCCCTTT TTTGCAACTT TCTTATTATC CATTCCATTC ATTTTTT--- ---------- ---------- ----------
HapH                AAATCAAATC TTTTTAATTA GTTCCCCTTT TTTGCAACTT TCTTATTATC CATTCCATTC ATTTTTT--- ---------- ---------- ----------
HapG                AAATCAAATC TTTTTAATTA GTTCCCCTTT TTTGCAACTT TCTTATTATC CATTCCATTC ATTTTTT--- ---------- ---------- ----------
HapAB               AAATCAAATC TTTTTAATTA GTTCCCCTTT TTTGCAACTT TCTTATTATC CATTCCATTC ATTTTTT--- ---------- ---------- ----------
HapZ                AAATCAAATC TTTTTAATTA GTTCCCCTTT TTTGCAACTT TCTTATTATC CATTCCATTC ATTTTTT--- ---------- ---------- ----------
HapD                AAATCAAATC TTTTTAATTA GTTCCCCTTT TTTGCAACTT TCTTATTATC CATTCCATTC ATTTTTTTTT --CTAGATAT AGAATACTTT TATAGAATAC
HapA                AAATCAAATC TTTTTAATTA GTTCCCCTTT TTTGCAACTT TCTTATTATC CATTCCATTC ATTTTTTTTT --CTAGATAT AGAATACTTT TATAGAATAC
HapB                AAATCAAATC TTTTTAATTA GTTCCCCTTT TTTGCAACTT TCTTATTATC CATTCCATTC ATTTTTTTTT --CTAGATAT AGAATACTTT TATAGAATAC
HapC                AAATCAAATC TTTTTAATTA GTTCCCCTTT TTTGCAACTT TCTTATTATC CATTCCATTC ATTTTTTTTT --CTAGATAT AGAATACTTT TATAGAATAC
HapY                AAATCAAATC TTTTTAATTA GTTCCCCTTT TTTGCAACTT TCTTATTATC CATTCCATTC ATTTTTTT-- ---------- ---------- ----------
HapW                AAATAAAATC TTTTTAATTA GTTCCCCTTT TTTGCAACTT TCTTATTATC CATTCCATTC ATTTTTTTTT T-CTAGATAT AGAATACTTT TCTAGAATAC
HapX                AAATAAAATC TTTTTAATTA GTTCCCCTTT TTTGCAACTT TCTTATTATC CATTCCATTC ATTTTTTTTT T-CTAGATAT AGAATACTTT TATAGAATAC
HapS                AAATCAAATC TTTTTAATTA GTTCCCCTTT TTTGCAACTT TCTTATTATC CATTCCATTC ATTTTTT--- ---------- ---------- ----------
HaKonecNC_025235    AAATCAAATC TTTTTAATTA GTTCCCCTTT TTTGCAACTT TCTTATTATC CATTCCATTC ATTTTTTTTT TTCTAGA--- ---------- ----------
Monparadoxtrnt      AAATCAAATC TTTTTAATTA GTTCCC-TTT TTTGCAACTT TCTTATTATC CATTCCATTC ATTTTTTTTT T--------- ---------- ----------
```

```
                       ....|....| ....|....| ....|....| ....|....| ....|....| ....|....| ....|....| ....|....| ....|....| ....|....|
                          1805       1815       1825       1835       1845       1855       1865       1875       1885       1895

HapM                   -TTTCTAGAT ATAGAATACT TTTATAGAAT ACTAAAGTAT TCTAT----- -AAAAAATCT TTATTTTATT TTGCAAACTA AAAAATACAA TAGTCAATAT
HapM1                  -TTTCTAGAT ATAGAATACT TTTATAGAAT ACTAAAGTAT TCTAT----- -AAAAAATCT TTATTTTATT TTGCAAACTA AAAAATACAA TAGTCAATAT
FU12.1                 -TTTCTAGAT ATAGAATACT TTTATAGAAT ACTAAAGTAT TCTAT----- -AAAAAATCT TTATTTTATT TTGCAAACTA AAAAATACAA TAGTCAATAT
FU10.1                 -TTTCTAGAT ATAGAATACT TTTATAGAAT ACTAAAGTAT TCTAT----- -AAAAAATCT TTATTTTATT TTGCAAACTA AAAAATACAA TAGTCAATAT
FU1.1                  -TTTCTAGAT ATAGAATACT TTTATAGAAT ACTAAAGTAT TCTAT----- -AAAAAATCT TTATTTTATT TTGCAAACTA AAAAATACAA TAGTCAATAT
FU2.1                  -TTTCTAGAT ATAGAATACT TTTATAGAAT ACTAAAGTAT TCTAT----- -AAAAAATCT TTATTTTATT TTGCAAACTA AAAAATACAA TAGTCAATAT
FU11.1                 -TTTCTAGAT ATAGAATACT TTTATAGAAT ACTAAAGTAT TCTAT----- -AAAAAATCT TTATTTTATT TTGCAAACTA AAAAATACAA TAGTCAATAT
CO4.1                  -TTTCTAGAT ATAGAATACT TTTATAGAAT ACTAAAGTAT TCTAT----- -AAAAAATCT TTATTTTATT TTGCAAACTA AAAAATACAA TAGTCAATAT
CO11.1                 -TTTCTAGAT ATAGAATACT TTTATAGAAT ACTAAAGTAT TCTAT----- -AAAAAATCT TTATTTTATT TTGCAAACTA AAAAATACAA TAGTCAATAT
TR1.1                  -TTTCTAGAT ATAGAATACT TTTATAGAAT ACTAAAGTAT TCTAT----- -AAAAAATCT TTATTTTATT TTGCAAACTA AAAAATACAA TAGTCAATAT
TR3.1                  -TTTCTAGAT ATAGAATACT TTTATAGAAT ACTAAAGTAT TCTAT----- -AAAAAATCT TTATTTTATT TTGCAAACTA AAAAATACAA TAGTCAATAT
TR10.1                 -TTTCTAGAT ATAGAATACT TTTATAGAAT ACTAAAGTAT TCTAT----- -AAAAAATCT TTATTTTATT TTGCAAACTA AAAAATACAA TAGTCAATAT
TR11.1                 -TTTCTAGAT ATAGAATACT TTTATAGAAT ACTAAAGTAT TCTAT----- -AAAAAATCT TTATTTTATT TTGCAAACTA AAAAATACAA TAGTCAATAT
Vi16.1                 -TTTCTAGAT ATAGAATACT TTTATAGAAT ACTAAAGTAT TCTAT----- -AAAAAATCT TTATTTTATT TTGCAAACTA AAAAATACAA TAGTCAATAT
VI1.1                  -TTTCTAGAT ATAGAATACT TTTATAGAAT ACTAAAGTAT TCTAT----- -AAAAAATCT TTATTTTATT TTGCAAACTA AAAAATACAA TAGTCAATAT
Vi3.1                  -TTTCTAGAT ATAGAATACT TTTATAGAAT ACTAAAGTAT TCTAT----- -AAAAAATCT TTATTTTATT TTGCAAACTA AAAAATACAA TAGTCAATAT
CH1.1                  -TTTCTAGAT ATAGAATACT TTTATAGAAT ACTAAAGTAT TCTAT----- -AAAAAATCT TTATTTTATT TTGCAAACTA AAAAATACAA TAGTCAATAT
CH4.1                  -TTTCTAGAT ATAGAATACT TTTATAGAAT ACTAAAGTAT TCTAT----- -AAAAAATCT TTATTTTATT TTGCAAACTA AAAAATACAA TAGTCAATAT
CH6.1                  -TTTCTAGAT ATAGAATACT TTTATAGAAT ACTAAAGTAT TCTAT----- -AAAAAATCT TTATTTTATT TTGCAAACTA AAAAATACAA TAGTCAATAT
CH2.2                  -TTTCTAGAT ATAGAATACT TTTATAGAAT ACTAAAGTAT TCTAT----- -AAAAAATCT TTATTTTATT TTGCAAACTA AAAAATACAA TAGTCAATAT
CH2.1                  -TTTCTAGAT ATAGAATACT TTTATAGAAT ACTAAAGTAT TCTAT----- -AAAAAATCT TTATTTTATT TTGCAAACTA AAAAATACAA TAGTCAATAT
HapAD                  -TTTCTAGAT ATAGAATACT TTTATAGAAT ACTAAAGTAT TCTAT----- -AAAAAATCT TTATTTTATT TTGCAAACTA AAAAATACAA TAGTCAATAT
HapO                   -TTTCTAGAT ATAGAATACT TTTATAGAAT ACTAAAGTAT TCTAT----- -AAAAAATCT TTATTTTATT TTGCAAACTA AAAAATACAA TAGTCAATAT
HapN                   -TTTCTAGAT ATAGAATACT TTTATAGAAT ACTAAAGTAT TCTAT----- -AAAAAATCT TTATTTTATT TTGCAAACTA AAAAATACAA TAGTCAATAT
HapK                   -TTTCTAGAT ATAGAATACT TTTATAGAAT ACTAAAGTAT TCTAT----- -AAAAAATCT TTATTTTATT TTGCAAACTA AAAAATACAA TAGTCAATAT
HapJ                   -TTTCTAGAT ATAGAATACT TTTATAGAAT ACTAAAGTAT TCTAT----- -AAAAAATCT TTATTTTATT TTGCAAACTA AAAAATACAA TAGTCAATAT
HapL                   -TTTCTAGAT ATAGAATACT TTTATAGAAT ACTAAAGTAT TCTAT----- -AAAAAATCT TTATTTTATT TTGCAAACTA AAAAATACAA TAGTCAATAT
HapQ                   -TTTCTAGAT ATAGAATACT TTTATAGAAT ACTAAAGTAT TCTAT----- -AAAAAATCT TTATTTTATT TTGCAAACTA AAAAATACAA TAGTCAATAT
HapV                   -TTTCTAGAT ATAGAATACT TTTATAGAAT ACTAAAGTAT TCTAT----- -AAAAAATTT TTATTTTATT TTGCAAACTA AAAAATACAA TAGTCAATAT
HapR                   -TTTCTAGAT ATAGAATACT TTTATAGAAT ACTAAAGTAT TCTAT----- -AAAAAATCT TTATTTTATT TTGCAAACTA AAAAATACAA TAGTCAATAT
HapP                   -TTTCTAGAT ATAGAATACT TTTATAGAAT ACTAAAGTAT TCTAT----- -AAAAAATCT TTATTTTATT TTGCAAACTA AAAAATACAA TAGTCAATAT
HapU                   -TTTCTAGAT ATAGAATACT TTTATAGAAT ACTTTAGTAT TCTAT----- -AAAAAATCT TTATTTTATT TTGCAAACTA AAAAATACAA TAGTCAATAT
HapT                   -TTTCTAGAT ATAGAATACT TTTATAGAAT ACTAAAGTAT TCTAT----- -AAAAAATCT TTATTTTATT TTGCAAACTA AAAAATACAA TAGTCAATAT
HapAA                  -TTTCTAGAT ATAGAATACT TTTATAGAAT ACTAAAGTAT TCTAT----- -AAAAAATCT TTATTTTATT TTGCAAACTA AAAAATACAA TAGTCAATAT
HapH30                 -TTTCTAGAT ATAGAATACT TTTATAGAAT ACTAAAGTAT TCTAT----- -AAAAAATCT TTATTTTATT TTGCAAACTA AAAAATACAA TAGTCAATAT
HapI                   -TTTCTAGAT ATAGAATACT TTTATAGAAT ACTTTAGTAT TCTAT----- -AAAAAATCT TTATTTTATT TTGCAAACTA AAAAATACAA TAGTCAATAT
HapE                   -TTTCTAGAT ATAGAATACT TTTATAGAAT ACTAAAGTAT TCTAT----- -AAAAAATCT TTATTTTATT TTGCAAACTA AAAAATACAA TAGTCAATAT
HapAC                  -TTTCTAGAT ATAGAATACT TTTATAGAAT ACTAAAGTAT TCTAT----- -AAAAAATCT TTATTTTATT TTGCAAACTA AAAAATACAA TAGTCAATAT
HapF                   -TTTCTAGAT ATAGAATACT TTTATAGAAT ACTAAAGTAT TCTAT----- -AAAAAATCT TTATTTTATT TTGCAAACTA AAAAATACAA TAGTCAATAT
HapH                   -TTTCTAGAT ATAGAATACT TTTATAGAAT ACTAAAGTAT TCTAT----- -AAAAAATCT TTATTTTATT TTGCAAACTA AAAAATACAA TAGTCAATAT
HapG                   -TTTCTAGAT ATAGAATACT TTTATAGAAT ACTAAAGTAT TCTAT----- -AAAAAATCT TTATTTTATT TTGCAAACTA AAAAATACAA TAGTCAATAT
HapAB                  -TTTCTAGAT ATAGAATACT TTTATAGAAT ACTAAAGTAT TCTAT----- -AAAAAATCT TTATTTTATT TTGCAAACTA AAAAATACAA TAGTCAATAT
HapZ                   -TTTCTAGAT ATAGAATACT TTTATAGAAT ACTAAAGTAT TCTATAAAAA AAAAAAATCT TTATTTTATT TTGCAAACTA AAAAATACAA TAGTCAATAT
HapD                   -TTTCTAGAT ATAGAATACT TTTATAGAAT ACTAAAGTAT TCTAT----- -AAAAAATCT TTATTTTATT TTGCAAACTA AAAAATACAA TAGTCAATAT
HapA                   -TTTCTAGAT ATAGAATACT TTTATAGAAT ACTAAAGTAT TCTAT----- -AAAAAATCT TTATTTTATT TTGCAAACTA AAAAATACAA TAGTCAATAT
HapB                   -TTTCTAGAT ATAGAATACT TTTATAGAAT ACTAAAGTAT TCTAT----- -AAAAAATCT TTATTTTATT TTGCAAACTA AAAAATACAA TAGTCAATAT
HapC                   -TTTCTAGAT ATAGAATACT TTTATAGAAT ACTAAAGTAT TCTAT----- -AAAAAATCT TTATTTTATT TTGCAAACTA AAAAATACAA TAGTCAATAT
HapY                   -TTTCTAGAT ATAGAATACT TTTATAGAAT ACTAAAGTAT TCTAT----- -AAAAAATCT TTATTTTATT TTGCAAACTA AAAAATACAA TAGTCAATAT
HapW                   TTTTCTAGAT ATAGAATACT TTTCTAGAAT ACTAAAGTAT TCTAG----- -AAAAAATCT TTATTTTATT TTGCAAACTA AAAAATACAA TAGTCAATAT
HapX                   TTTTCTAGAT ATAGAATACT TTTCTAGAAT ACTAAAGTAT TCTAT----- -AAAAAATCT TTATTTTATT TTGCAAACTA AAAAATACAA TAGTCAATAT
HapS                   -TTTCTAGAT ATAGAATACT TTTATAGAAT ACTAAAGTAT TCTAT----- -AAAAAATCT TTATTTTATT TTGCAAACTA AAAAATACAA TAGTCAATAT
HaKonecNC_025235       -TTTCTAGAT ATAGAATACT TTTATAGAAT ACTTTAGTAT TCTAT----- -AAAAAATCT TTATTTTATT TTGCAAACTA AAAAATACAA TAGTCAATAT
Monparadoxtrnt         -----TAGAT ATAGC----- --------AT ACTAGAGTAT TCTAT----- -AAAAA---- -TTCTTTATT TTGCAAACTA AAAAATACAA TAGTCAATAT

                       ....|....| ....|....| ....|....| ....|....| ....|....| ....|....| ....|....| ....|....| ....|.
                          1905       1915       1925       1935       1945       1955       1965       1975       1985
HapM                   TC-------- ---------- ---CTTATAA TAGATATACT TAATTATATC ATAAGATTCT TATGATATAT TTCGAATAGA TAGAAA
HapM1                  TC-------- ---------- ---CTTATAA TAGATATACT TAATTATATC ATAAGATTCT TATGATATAT TTCGAATAGA TAGAAA
FU12.1                 TC-------- ---------- ---CTTATAA TAGATATACT TAATTATATC ATAAGATTCT TATGATATAT TTCGAATAGA TAGAAA
FU10.1                 TC-------- ---------- ---CTTATAA TAGATATACT TAATTATATC ATAAGATTCT TATGATATAT TTCGAATAGA TAGAAA
FU1.1                  TC-------- ---------- ---CTTATAA TAGATATACT TAATTATATC ATAAGATTCT TATGATATAT TTCGAATAGA TAGAAA
FU2.1                  TC-------- ---------- ---CTTATAA TAGATATACT TAATTATATC ATAAGATTCT TATGATATAT TTCGAATAGA TAGAAA
FU11.1                 TC-------- ---------- ---CTTATAA TAGATATACT TAATTATATC ATAAGATTCT TATGATATAT TTCGAATAGA TAGAAA
CO4.1                  TC-------- ---------- ---CTTATAA TAGATATACT TAATTATATC ATAAGATTCT TATGATATAT TTCGAATAGA TAGAAA
CO11.1                 TC-------- ---------- ---CTTATAA TAGATATACT TAATTATATC ATAAGATTCT TATGATATAT TTCGAATAGA TAGAAA
TR1.1                  TC-------- ---------- ---CTTATAA TAGATATACT TAATTATATC ATAAGATTCT TATGATATAT TTCGAATAGA TAGAAA
TR3.1                  TC-------- ---------- ---CTTATAA TAGATATACT TAATTATATC ATAAGATTCT TATGATATAT TTCGAATAGA TAGAAA
TR10.1                 TC-------- ---------- ---CTTATAA TAGATATACT TAATTATATC ATAAGATTCT TATGATATAT TTCGAATAGA TAGAAA
TR11.1                 TC-------- ---------- ---CTTATAA TAGATATACT TAATTATATC ATAAGATTCT TATGATATAT TTCGAATAGA TAGAAA
Vi16.1                 TC-------- ---------- ---CTTATAA TAGATATACT TAATTATATC ATAAGATTCT TATGATATAT TTCGAATAGA TAGAAA
VI1.1                  TC-------- ---------- ---CTTATAA TAGATATACT TAATTATATC ATAAGATTCT TATGATATAT TTCGAATAGA TAGAAA
Vi3.1                  TC-------- ---------- ---CTTATAA TAGATATACT TAATTATATC ATAAGATTCT TATGATATAT TTCGAATAGA TAGAAA
CH1.1                  TC-------- ---------- ---CTTATAA TAGATATACT TAATTATATC ATAAGATTCT TATGATATAT TTCGAATAGA TAGAAA
CH4.1                  TC-------- ---------- ---CTTATAA TAGATATACT TAATTATATC ATAAGATTCT TATGATATAT TTCGAATAGA TAGAAA
CH6.1                  TC-------- ---------- ---CTTATAA TAGATATACT TAATTATATC ATAAGATTCT TATGATATAT TTCGAATAGA TAGAAA
CH2.2                  TC-------- ---------- ---CTTATAA TAGATATACT TAATTATATC ATAAGATTCT TATGATATAT TTCGAATAGA TAGAAA
CH2.1                  TC-------- ---------- ---CTTATAA TAGATATACT TAATTATATC ATAAGATTCT TATGATATAT TTCGAATAGA TAGAAA
HapAD                  TC-------- ---------- ---CTTATAA TAGATATACT TAATTATATC ATAAGATTCT TATGATATAT TTCGAATAGA TAGAAA
HapO                   TC-------- ---------- ---CTTATAA TAGATATACT TAATTATATC ATAAGATTCT TATGATATAT TTCGAATAGA TAGAAA
HapN                   TC-------- ---------- ---CTTATAA TAGATATACT TAATTATATC ATAAGATTCT TATGATATAT TTCGAATAGA TAGAAA
HapK                   TC-------- ---------- ---CTTATAA TAGATATACT TAATTATATC ATAAGATTCT TATGATATAT TTCGAATAGA TAGAAA
HapJ                   TC-------- ---------- ---CTTATAA TAGATATACT TAATTATATC ATAAGATTCT TATGATATAT TTCGAATAGA TAGAAA
HapL                   TC-------- ---------- ---CTTATAA TAGATATACT TAATTATATC ATAAGATTCT TATGATATAT TTCGAATAGA TAGAAA
HapQ                   TC-------- ---------- ---CTTATAA TAGATATACT TAATTATATC ATAAGATTCT TATGATATAT TTCGAATAGA TAGAAA
HapV                   TC-------- ---------- ---CTTATAA TAGATATACT TAATTATATC ATAAGATTCT TATGATATAT TTCGAATAGA TAGAAA
HapR                   TC-------- ---------- ---CTTATAA TAGATATACT TAATTATATC ATAAGATTCT TATGATATAT TTCGAATAGA TAGAAA
HapP                   TC-------- ---------- ---CTTATAA TAGATATACT TAATTATATC ATAAGATTCT TATGATATAT TTCGAATAGA TAGAAA
HapU                   TC-------- ---------- ---CTTATAA TAGATATACT TAATTATATC ATAAGATTCT TATGATATAT TTCGAATAGA TAGAAA
HapT                   TCCTTAATAC AATAGTCAAT ATTCTTATAA TAGATATACT TAATTATATC ATAAGATTCT TATGATATAT TTCGAATAGA TAGAAA
HapAA                  TC-------- ---------- ---CTTATAA TAGATATACT TAATTATATC ATAAGATTCT TATGATATAT TTCGAATAGA TAGAAA
HapH30                 TC-------- ---------- ---CTTATAA TAGATATACT TAATTATATC ATAAGATTCT TATGATATAT TTCGAATAGA TAGAAA
HapI                   TC-------- ---------- ---CTTATAA TAGATATACT TAATTATATC ATAAGATTCT TATGATATAT TTCGAATAGA TAGAAA
HapE                   TC-------- ---------- ---CTTATAA TAGATATACT TAATTATATC ATAAGATTCT TATGATATAT TTCGAATAGA TAGAAA
HapAC                  TC-------- ---------- ---CTTATAA TAGATATACT TAATTATATC ATAAGATTCT TATGATATAT TTCGAATAGA TAGAAA
HapF                   TC-------- ---------- ---CTTATAA TAGATATACT TAATTATATC ATAAGATTCT TATGATATAT TTCGAATAGA TAGAAA
HapH                   TC-------- ---------- ---CTTATAA TAGATATACT TAATTATATC ATAAGATTCT TATGATATAT TTCGAATAGA TAGAAA
HapG                   TC-------- ---------- ---CTTATAA TAGATATACT TAATTATATC ATAAGATTCT TATGATATAT TTCGAATAGA TAGAAA
HapAB                  TC-------- ---------- ---CTTATAA TAGATATACT TAATTATATC ATAAGATTCT TATGATATAT TTCGAATAGA TAGAAA
HapZ                   TC-------- ---------- ---CTTATAA TAGATATACT TAATTATATC ATAAGATTCT TATGATATAT TTCGAATAGA TAGAAA
HapD                   TC-------- ---------- ---CTTATAA TAGATATACT TAATTATATC ATAAGATTCT TATGATATAT TTCGAATAGA TAGAAA
HapA                   TC-------- ---------- ---CTTATAA TAGATATACT TAATTATATC ATAAGATTCT TATGATATAT TTCGAATAGA TAGAAA
HapB                   TC-------- ---------- ---CTTATAA TAGATATACT TAATTATATC ATAAGATTCT TATGATATAT TTCGAATAGA TAGAAA
HapC                   TC-------- ---------- ---CTTATAA TAGATATACT TAATTATATC ATAAGATTCT TATGATATAT TTCGAATAGA TAGAAA
HapY                   TC-------- ---------- ---CTTATAA TAGATATACT TAATTATATC ATAAGATTCT TATGATATAT TTCGAATAGA TAGAAA
HapW                   TC-------- ---------- ---CTTATAA TAGATATACT TAATTATATC ATAAGATTCT TATGATATAT TTCGAATAGA TAGAAA
HapX                   TC-------- ---------- ---CTTATAA TAGATATACT TAATTATATC ATAAGATTCT TATGATATAT TTCGAATAGA TAGAAA
HapS                   TC-------- ---------- ---CTTATAA TAGATATACT TAATTATATC ATAAGATTCT TATGATATAT TTCGAATAGA TAGAAA
HaKonecNC_025235       TC-------- ---------- ---CTTATAA TAGATATACT TAATTATATC ATAAGAATCT TAAGATATAT TTCGAATAGA TAGAAA
Monparadoxtrnt         TC-------- ---------- ---TTTATAA TAGATATACT TAATTATATC ATAAGAATCT TAAGATATAT TTCGAATAGA TAGAAA
```
