# Supplementary material for: AFLP Approach Reveals Variability in Phragmites australis: Implications for Its Die-Back and Evidence for Genotoxic Effects
Source: Front Plant Sci. 2018 Mar 26;9:386. doi: 10.3389/fpls.2018.00386 (PMC5879127; doi:10.3389/fpls.2018.00386)
Supplement: Supplementary file 3 [file Table_3.docx]

Supplementary Table S3. Primers name, primers sequence, selective extension and fluorescent labelling.

| **Primer ID** | **Primer sequence** | **Selective extension** | **Primer modification** |
| --- | --- | --- | --- |
| PEcoRI | **GACTGCGTACCAATTC** | CTA | 5’ Hexachloro-Fluorescein (HEX) |
| PEcoRI | **GACTGCGTACCAATTC** | TAC | 5’ 6-fluorescein amidite (FAM) |
| PMseI | **GATGAGTCCTGAGTAA** | TTA |  |
| PMseI | **GATGAGTCCTGAGTAA** | GAT |  |
| PMseI | **GATGAGTCCTGAGTAA** | CTT |  |
| PMseI | **GATGAGTCCTGAGTAA** | CTC |  |
| PMseI | **GATGAGTCCTGAGTAA** | CTA |  |
| PMseI | **GATGAGTCCTGAGTAA** | ATG |  |
| PMseI | **GATGAGTCCTGAGTAA** | TTA |  |
| PMseI | **GATGAGTCCTGAGTAA** | GAT |  |
